# Liquid-Assisted Grinding Enables Efficient Ni-Catalyzed, Mn-Mediated Denitrogenative Cross-Electrophile Coupling of Benzotriazinones with Benzyl Chlorides

**DOI:** 10.3390/molecules30051060

**Published:** 2025-02-26

**Authors:** Xuanxuan Zhang, Yingying Hong, Gang Zou

**Affiliations:** School of Chemistry and Molecular Engineering, East China University of Science and Technology, 130 Meilong Rd, Shanghai 200237, China; y30220291@mail.ecust.edu.cn (X.Z.); y30220303@mail.ecust.edu.cn (Y.H.)

**Keywords:** diarylmethane, benzotriazinone, cross-electrophile coupling, nickel catalysis, benzylation

## Abstract

An efficient and air-tolerant Ni-catalyzed denitrogenative cross-electrophile coupling of benzotriazinones with benzyl chlorides has been developed via liquid-assisted grinding by using Mn powders as reductant and DMF as assisting liquid in the presence of anhydrous calcium chloride. Scope and limitations of the protocol to access diarylmethanes have been demonstrated with more than 20 examples, showing acceptable tolerance to functional group and steric hindrance. Although electron-withdrawing substituents on benzotriazinone or benzyl counterparts decrease the yields significantly, a series of N-alkyl-2-benzylbenzamides, diarylmethanes bearing an ortho-carbamoyl aryl group, could be obtained in modest to good yields.

## 1. Introduction

Construction of diarylmethanes, an important structural motif in pharmaceuticals, natural products and supramolecular architectures, has attracted intense interest [1,2]. The most traditional access to diarylmethanes, Friedel–Crafts alkylation of benzyl compounds, suffers from over-alkylation, low regioselectivity, and/or limited functional group compatibility [3,4]. Transition metal catalyzed cross-couplings have proven to be effective in circumventing these problems. However, the cross-couplings of organometallic nucleophiles are often hampered by their preparation, which is often tedious or expensive and sometimes requires harsh conditions. Benefiting from the great advance in transition metal-catalyzed cross-electrophile coupling (XEC) [5,6,7,8,9,10] that eliminates the preparation and handling of organometallics, XEC-based benzylation has been recently developed for an efficient access to functional diarylmethanes. In fact, Lipshutz and co-workers reported an effective palladium-catalyzed zinc-mediated cross-coupling between benzyl and aryl halides ‘on water’ for synthesis of diarylmethanes as early as 2010 [11]. Weix and co-workers reported a Ni/Co co-catalyzed XEC of in situ-formed benzyl mesylates with aryl halides via a single-electron transfer or radical pathway in 2015 [12]. Thereafter, benzyl chlorides [13,14,15,16,17], alcohols [18,19] and other benzylic derivatives, e.g., oxalates [20], acetals [21], ammonium salts [22,23] and Katritzky salts [24] etc., have been applied in nickel-catalyzed benzylation of aryl (pseudo)halides.

Compared with conventional solution chemistry, mechanochemistry via ball-milling features solvent-free conditions, thus no solubility limitation, and efficient solid material mixing [25,26,27,28,29,30] as well as mechanical metal activation [31]. In fact, mechanochemical metal activation has been successfully applied in Reformatsky (Zn) reaction [32], Grignard-type (Mg/Ca) reaction [33,34,35], Negishi coupling (Zn) [36], XEC of aryl/alkyl halides (Zn, Mn) [37,38], Chan−Lam C−N coupling (Cu) [39] and metallic Li- or Mn-based reduction [40,41]. However, solvent-free ball milling performs ineffectively in many cases of soft organic materials. Fortunately, a small amount of proper liquid additive has proven to be very helpful in the grinding process, that is, the so-called liquid-assisted grinding (LAG) [29].

We have recently reported an efficient Ni(bpy)Cl_2_-catalyzed, Mn-mediated denitrogenative XEC of benzotriazinones, an easily accessible anthranilic acid derivative [42], with non-activated primary/secondary alkyl halides to afford ortho-alkylated secondary benzamides both in DMA solution [43] and under mechanoredox LAG conditions [44]. Given the importance of diarylmethanes in particular, the limited protocols to those bearing an ortho-carbamoyl aryl group, i.e., ortho-benzylbenzamides [45], we further report herein a liquid-assisted grinding (LAG)-enabled efficient denitrogenative benzylation of benzotriazinones via nickel-catalyzed, manganese-mediated XEC with benzyl chlorides for efficient access to ortho-benzylated N-alkylbenzamides.

## 2. Results and Discussion

**Scheme 1:**
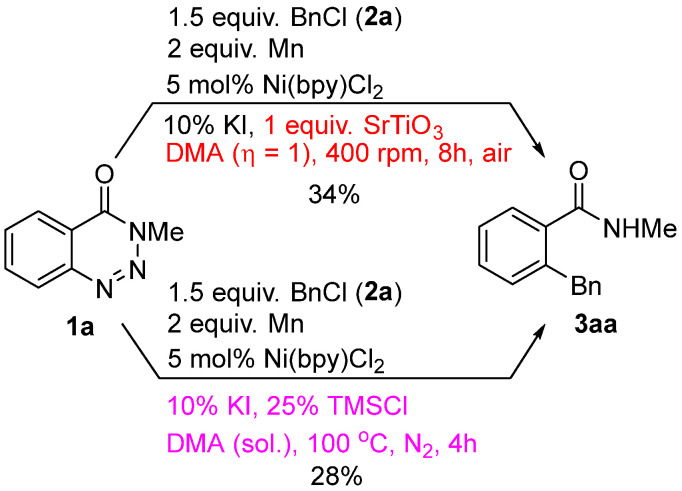
Preliminary results of denitrogenative XEC-based benzylation of benzotriazinones.

Initially, the Ni(bpy)Cl_2_/Mn systems we have developed for denitrogenative XEC of benzotriazinones with non-activated alkyl halides were adopted for the model reaction of N-methylbenzotriazinone (**1a**) with benzyl chloride (**2a**). However, the reaction proceeded sluggishly both in DMA solution and under mechanoredox LAG conditions. More than half of benzotriazinone **1a** could not be converted, giving 28–34% yields for **3aa** with some proto-denitrogenation side-product, while benzyl chloride **2a** was completely consumed (Scheme 1).

The low conversion of benzotriazinone **1a** vs. the complete consumption of 1.5 equiv. benzyl chloride **2a** implied that their reactivities should mismatch, possibly due to the high reactivity of benzyl chloride but the relatively low reactivity of benzyl radical in the Ni-catalyzed process. Considering the easier formation of benzyl radicals compared to non-activated alkyl ones, the piezoelectric SrTiO_3_ used to facilitate generation of the alkyl radicals should be not necessary, if not deleterious, for benzyl chloride. In addition, in situ formation of labile benzyl iodide in the presence of iodide co-catalyst may also partly contribute to the fast consumption of benzyl chloride. Therefore, we investigated the model reaction under LAG conditions without any piezoelectric and iodide additives (Table 1). Although disappointing results were obtained with DMA, CH_3_CN, THF or dioxane as assisting liquid, the desired XEC product **3aa** could be isolated in 26% yield using 1 μL/mg (η = 1) DMF (Table 1, entries 1–5), implying that DMF should not only serve as the assisting liquid for grinding but also facilitate the catalytic process. Given that strict anhydrous conditions are often required in Ni-catalyzed, metal-mediated XEC processes, a series of inorganic salts were tested as desiccant to remove the possible trace water in the system. To our delight, a couple of common salts, e.g., MgSO_4_ (50%), MgCl_2_ (65%), ZnCl_2_ (58%), LiCl (66%) and CaCl_2_ (75%), worked well to produce **3aa** in remarkably higher yields, although NaCl (27%), Na_2_SO_4_ (30%) and BaCl_2_ (36%) showed few effects (Table 1, entries 6–14). However, the yields could not be further increased by increasing the doses of CaCl_2_ (1.5 equiv.) or LiCl (2 equiv.) (Table 1, entries 9, 11, 15 and 17).

Encouraged by the above results, the XEC-based denitrogenative benzylation of benzotriazinones with benzyl chlorides was further optimized with respect to nickel catalysts, metallic reductants and substrate ratios under LAG conditions with DMF as the assisting liquid in the presence of 1 equiv. CaCl_2_ (Table 2). Nickel bromide Ni(bpy)Br_2_ performed similarly to its cheaper chloride analog. The yield decreased significantly to 49% with 3 mol% Ni(bpy)Cl_2_. In fact, almost no reaction took place with 1 mol% catalyst loading (Table 2, entries 1–3). Direct combination of nickel salts NiCl_2_, Ni(OAc)_2_ or Ni(acac)_2_ with 2,2′-bipyridine (bpy) to in situ form the bpy-supported nickel catalysts also worked effectively. However, replacement of bpy ligand by 4,4′-di(tert-butyl)-2,2′-bipyridine (dtbbpy, 42%), 1,10-phenanthroline (phen, 30%) or terpyridine (tpy, trace) led to remarkable decreases in **3aa** yields (Table 2, entries 4–9). Almost no reaction occurred without assisting liquid DMF. The optimal ratio of DMF to solid materials (η) appeared to be 1 μL/mg (η = 1) since the lower ratio, e.g., η = 0.5, led to a very low yield (8%), while the higher ratios, e.g., η = 1.5–2.0, could not increase the yields (Table 2, entries 11–13). The other commonly used metallic reductant Zn dust gave a low yield (18%) while Al powders did not work at all. An increase in BnCl stoichiometry from 1.5 to 2.0 equiv. resulted in a cream-like mixture and incomplete conversion of benzotriazinone **1a**, which could be circumvented by increasing DMF dose at the same time. In fact, **3aa** yields could be increased to 84–85% using 2.0–2.5 equiv. BnCl with slightly higher DMF ratios (η = 1.5–2.0) (Table 2, entries 19–23). Higher catalyst loading, e.g., 10 mol%, made the model reaction proceed faster (5 h), but failed to increase the product yield.

Input energy and mixing efficiency as reflected by rotational speed of ball-milling, ball number and filling degree of jar, were also briefly investigated and showed effects to some extent. The model reaction could not complete in 8 h with 8 grinding balls (ø = 7 mm) at 400 rpm or 16 balls but at 300 rpm, giving **3aa** in 61% and 73% yields, respectively (Table 2, entries 25 and 26).

**Figure 1:**
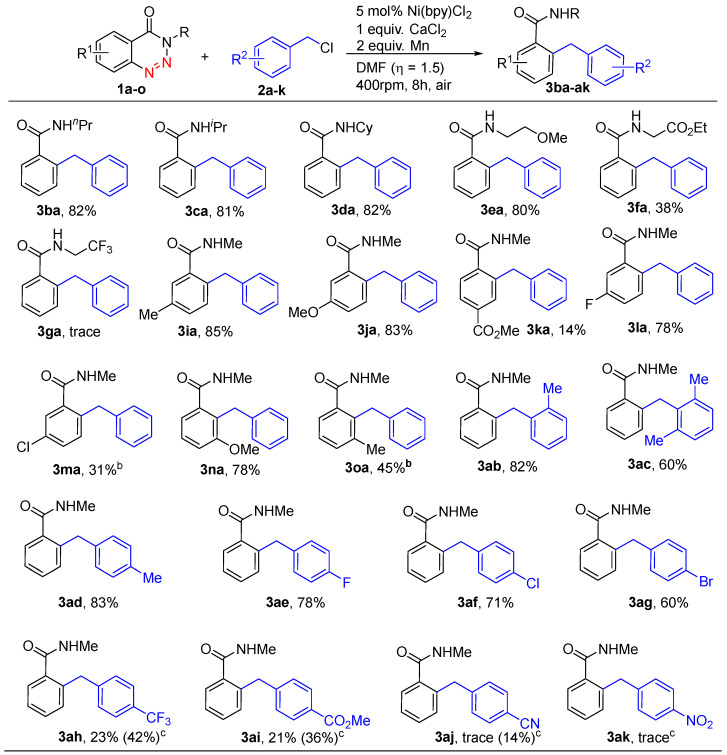
Optimization of the XEC-based denitrogenative benzylation of benzotriazinones ^a^. ^a^ Reaction run in air at 2 mmol scale with respect to **1a**-**o** using 2.0 equiv. **2a**-**k**, 5 mol% Ni(bpy)Cl_2_, 2.0 equiv. Mn powders, 1.0 equiv. CaCl_2_, 16 stainless-steel balls (ø = 7 mm) in 50 mL jar at 400 rpm in the presence of DMF (η = 1.5) assisting liquid and isolated yields reported for **3**. ^b^ More proto-denitrogenation observed. ^c^ With 10 mol% catalyst.

Based on these experiments, the optimal parameters for the Ni-catalyzed, Mn-mediated mechanochemical denitrogenative XEC-based benzylation of benzotriazinones were set as 2.0 equiv. benzyl chloride, 5 mol % of Ni(bpy)Cl_2_ catalyst, 2.0 equiv. Mn powders as reductant in the presence of 1.0 equiv. CaCl_2_ and 1.5 μL/mg (η = 1.5) DMF as assisting liquid under ball-milling at 400 rpm with 16 stainless-steel balls (ø = 7 mm) in each 50 mL jar. Scope and limitations of the protocol to access diarylmethanes bearing an ortho-carbamoyl aryl group were explored briefly under the optimal conditions (Figure 1).

**Scheme 2:**
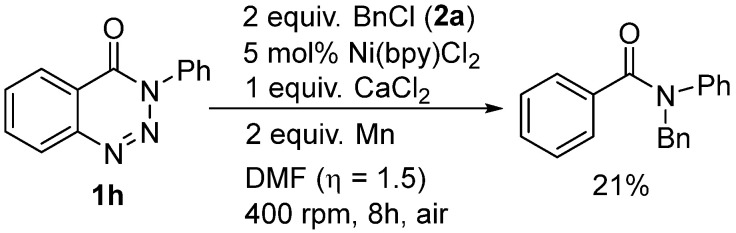
N-Benzylation after proto-denitrogenation of N-phenyl benzotriazinone **1h**.

Electron-deficiency of N-substituents of benzotriazinones decreased the product (**3fa** and **3ga**) yields remarkably while their steric hindrance appeared to be negligible. For example, N-propyl (**1b**, 82%), iso-propyl (**1c**, 81%), cyclohexyl (**1d**, 82%) and N-2-methoxyethyl (**1e**, 80%) benzotriazinones reacted similarly to N-methyl one (**1a,** 84%) while N-(2-ethoxyoxoethyl) benzotriazinone (**1f**) gave a low yield (38%). Further, no desired XEC products were isolated from the reaction of N-(2,2,2-trifluoroethyl) (**1g**) or N-phenyl (**1h**) benzotriazinones. After proto-denitrogenation took place, even N-benzylation produced N-benzyl-N-phenylbenzamide in 21% yield in the case of **1h** (Scheme 2). These electronic effects of N-substituents are consistent with those previously reported on stability/reactivity of benzotriazinones [42,43,46,47].

Similarly, electronic factors of the substituents on benzotriazinone core also remarkably affected the denitrogenative XEC with benzyl chloride. For example, 6-methyl (**1i**, 85%) and 6-methoxyl (**1j**, 83%) N-methylbenzotriazinones reacted smoothly while 7-methoxycarbonyl (**1k**) substituent led to a complex reaction, from which the desired product (**3ka**) was isolated in only 14% yield. 6-Fluoro-N-methylbenzotriazinone (**1l**, 78%) reacted smoothly while its chloride analog (**1m**) gave a low yield (31%). Steric hindrance of the benzotriazinone core could be tolerated to some extent. For example, 3, 8-dimethyl benzotriazinone (**1o**) could still react to give **3oa** in 45% yield. Surprisingly, a good yield (78%) could be obtained from 8-methoxyl-N-methylbenzotriazinone (**1n**), possibly due to the intramolecular O to Ni coordination, which could stabilize the key intermediates, azanickelacycles, in the catalytic cycle (vide infra).

On the benzyl counterpart, steric hindrance from aryl ring could be well tolerated although no desired XEC was observed for sec-benzyl chloride (1-chloroethyl)benzene. For example, 2-methyl (**2b**) and 4-methyl (**2d**) benzyl chlorides reacted comparably to give **3ab** (82%) and **3ad** (83%) in good yields, respectively. 2,6-Dimethyl benzyl chloride (**2c**) could still gave 60% yield for **3ac**. Surprisingly, the C_Ar_-Br group in benzyl chloride could survive in the nickel catalysis since 2-(4-bromobenzyl)-N-methylbenzamide **3ag** could be isolated in 60% yield from the reaction of *p*-bromobenzyl chloride **2g** with **1a**, just slightly lower than its fluoride (**3ae**, 78%) and chloride (**3af**, 71%) analogs. However, electron-withdrawing substituents led to sluggish reaction. In fact, benzyl chlorides bearing a para- electron-withdrawing group gave very low yields, e.g., *p*-CF_3_ (**3ah**, 23%), *p*-CO_2_Me (**3ai**, 21%) and *p*-CN (**3aj**, trace), which could be slightly increased to 14–42% by using 10 mol% catalyst loading. The nickel catalyst system appeared to be incompatible with NO_2_ group since no reaction was observed for *p*-nitrobenzyl chloride **2k** even at 10 mol% catalyst loading.

**Scheme 3:**
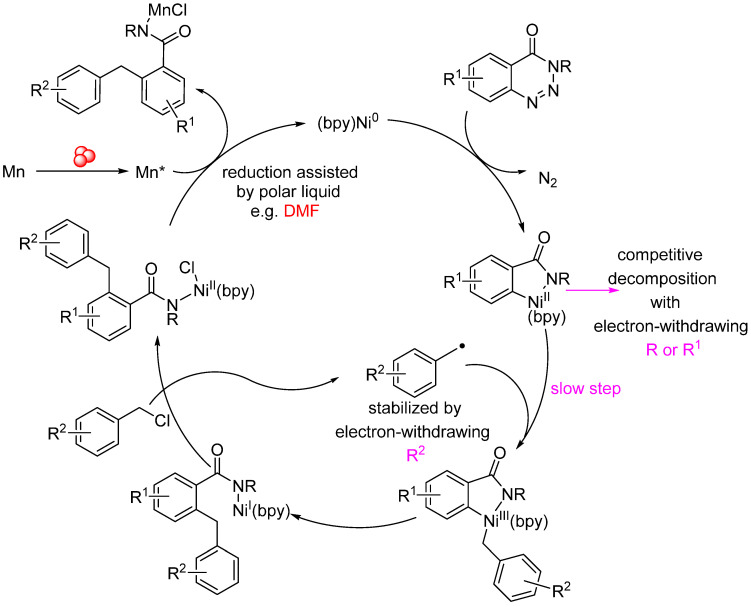
An explanation for the substrate structural effects based on the previously proposed catalytic cycle. * denotes activated Mn powders.

It should be reasonable for the XEC-based denitrogenative benzylation of benzotriazinones to follow the previously proposed catalytic cycle for non-activated halides [43], which contains a Ni(II→III→I→II→0) sequence: (1) denitrogenative oxidative addition of Ni(0) to benzotriazinone affording a five-membered Ni(II) azanickelacycle, (2) single-electron oxidation of the Ni(II) azanickelacycle by benzyl radical generated from chloride giving benzyl Ni(III) azanickelacycle, (3) subsequent reductive elimination forming the diarylmethane moiety in resulted Ni(I) amido intermediate, (4) single-electron oxidation of the Ni(I) species by benzyl chloride producing Ni(II) amido intermediate and benzyl radical, and finally, (5) reduction of the amido Ni(II) by mechanochemically activated Mn(0) to generate the catalytically active Ni(0) species and manganese salt of the product, which will hydrolyze during workup to give the final product (Scheme 3). The observed structural effects from both benzotriazinones and benzyl chlorides could be attributed to the lower reactivity of benzyl radicals than that of non-activated alkyl ones in the key single-electron oxidation of Ni(II) to BnNi(III) intermediates. On one hand, the low reactivity of benzyl radicals, in particular those bearing electron-deficient aryl group, would lead to the slow single-electron oxidation of Ni(II) to BnNi(III) azanickelacycles. On the other hand, an electron-withdrawing substituent, whether on the core or at N atom, would decrease the stability of benzotriazinones and/or the subsequent azanickelacycles. The fact that the coordinative 8-methoxyl substituted N-methylbenzotriazinone **1n** gave a higher yield (78%) than the 8-methyl substituted **1o** (45%) supports the importance of the stability of the azanickelacycle intermediates in the nickel-catalyzed process.

## 3. Materials and Methods

### 3.1. General Information

All operations were carried out under air unless otherwise stated. Ball-milling was carried out using a planetary ball mill (QM-3SP2, Nanjing Nanda Instrument Co., Ltd., Nanjing, China) equipped with four (50 mL) stainless steel jars and 8 or 16 stainless steel balls (ø = 7 mm) at given rpms. Chemicals obtained from commercial sources were used as received. Nickel complex Ni(bpy)Cl_2_ and benzotriazinones were prepared according to previous procedures [43]. Column chromatography was performed on 300–400 mesh silica gel. ^1^H and ^13^C{H}NMR spectra were recorded in CDCl_3_ at ambient temperature on a Bruker DPX-400 spectrometer (Bruker BioSpin GmbH, Rheinstetten, Germany). Chemical shifts (δ) in NMR are reported in ppm, relative to the internal standard of tetramethylsilane (TMS) or residues of the deuterated solvents. Coupling constants J are reported in Hz. Proton coupling patterns are described as singlet (s), doublet (d), triplet (t), quartet (q), and multiple (m). All new compounds were further characterized by high-resolution mass spectra (HRMS) using an Agilent mass spectrometer (HR-TOF-MS, ESI).

### 3.2. General Procedure for Nickel-Catalyzed Manganese-Mediated Denitrogenative Cross-Electrophile Coupling of Benzotriazinones with Benzyl Chlorides Under LAG Condition

To a 50 mL stainless steel milling jar were added benzotriazinone (2.0 mmol, 1.0 equiv.), benzyl chloride (4 mmol, 2 equiv.), Ni(bpy)Cl_2_ (28.6 mg, 0.1 mmol, 5 mol%), Mn powers (220 mg, 4.0 mmol, 2.0 equiv.), CaCl_2_ (221mg, 2 mmol, 1.0 equiv.) and DMF (η = 1.5). The mixture was grinded on a planetary ball mill at 400 rpm, with 16 stainless-steel balls (ø = 7 mm) for the given time. Then, samples were taken for TLC analysis and the mixture was transferred from the milling jar into a flask with the aid of CH_2_Cl_2_ (3 × 10 mL). The organic solution was washed with brine and dried over anhydrous Na_2_SO_4_. After filtration, solvents were removed by rotavapor to give the crude product, which was purified by flash column chromatography on silica gel using a gradient of ethyl acetate in petroleum ether.

*2-Benzyl-N-methylbenzamide (***3aa***)* [48]: White solid (376.1 mg, 84%); m.p.: 108–110 °C. ^1^H NMR (400 MHz, CDCl_3_) δ (ppm): 7.34–7.30 (m, 2H), 7.25–7.14 (m, 7H), 5.65 (s, 1H), 4.15 (s, 2H), 2.81 (d, *J* = 4.8 Hz, 3H). ^13^C NMR (100 MHz, CDCl_3_) δ (ppm): 170.8, 140.9, 138.9, 136.9, 130.9, 129.9, 129.0, 128.5, 127.2, 126.4, 126.2, 39.0, 26.6.

*2-Benzyl-N-propylbenzamide (***3ba***)*: White solid (415.5 mg, 82%); m.p.: 81–82 °C. ^1^H NMR (400 MHz, CDCl_3_) δ (ppm): 7.38–7.32 (m, 2H), 7.28–7.16 (m, 7H), 5.68 (s, 1H), 4.19 (s, 2H), 3.27 (q, *J* =6.4 Hz, 2H), 1.51–1.42 (m, 2H), 0.87 (t, *J* = 7.6 Hz, 3H). ^13^C NMR (100 MHz, CDCl_3_) δ (ppm): 170.1, 140.9, 138.7, 137.1, 131.0, 129.9, 129.0, 128.5, 127.2, 126.4, 126.1, 41.6, 38.9, 22.7, 11.4. HRMS (ESI) *m*/*z*: [M + H]^+^ calcd for C_17_H_20_NO 254.1545; found 254.1543.

*2-Benzyl-N-isopropylbenzamide (***3ca***)*: White solid (411.4 mg, 81%); m.p.: 118–119 °C. ^1^H NMR (400 MHz, CDCl_3_) δ (ppm): 7.36–7.31 (m, 2H), 7.27–7.14 (m, 7H), 5.45 (s, 1H), 4.18 (s, 2H), 4.15–4.09 (m, 1H), 1.07 (d, *J* = 6.8 Hz, 6H). ^13^C NMR (100 MHz, CDCl_3_) δ (ppm): 169.2, 141.0, 138.5, 137.2, 131.1, 129.9, 128.9, 128.5, 127.3, 126.4, 126.1, 41.7, 38.9, 22.6. HRMS (ESI) *m*/*z*: [M + Na]^+^ calcd for C_17_H_19_NONa 276.1364; found 276.1365.

*2-Benzyl-N-cyclohexylbenzamide (***3da***)* [49]: White solid (480.5 mg, 82%); m.p.: 148–149 °C. ^1^H NMR (400 MHz, CDCl_3_) δ (ppm): 7.37–7.31 (m, 2H), 7.27–7.14 (m, 7H), 5.51 (d, *J* = 7.2 Hz, 1H), 4.18 (s, 2H), 3.90–3.80 (m, 1H), 1.88–1.85 (m, 2H), 1.66–1.57 (m, 3H), 1.41–1.31 (m, 2H), 1.16–1.10 (m, 1H), 1.03–0.97 (m, 2H). ^13^C NMR (100 MHz, CDCl_3_) δ (ppm): 169.1, 141.0, 138.5, 137.3, 131.0, 129.9, 129.0, 128.5, 127.3, 126.4, 126.1, 48.5, 38.9, 32.9, 25.6, 24.8.

*2-Benzyl-N-(2-methoxyethyl)benzamide (***3ea***)*: White solid (430.9 mg, 80%); m.p.: 63–64 °C. ^1^H NMR (400 MHz, CDCl_3_) δ (ppm): 7.39–7.31 (m, 2H), 7.27–7.15 (m, 7H), 6.03 (s, 1H), 4.19 (s, 2H), 3.52–3.48 (m, 2H), 3.38 (t, *J* = 5.0 Hz, 2H), 3.26 (s, 3H). ^13^C NMR (100 MHz, CDCl_3_) δ (ppm): 170.0, 140.9, 139.0, 136.7, 131.0, 130.0, 129.0, 128.4, 127.2, 126.4, 126.1, 71.0, 58.7, 39.5, 38.9. HRMS (ESI) *m*/*z*: [M + Na]^+^ calcd for C_17_H_19_NO_2_Na 292.1313; found 292.1312.

*Ethyl (2-benzylbenzoyl)glycinate (***3fa***)*: White solid (226.1 mg, 38%); m.p.: 85–87 °C. ^1^H NMR (400 MHz, CDCl_3_) δ (ppm): 7.43 (d, *J* = 7.6 Hz, 1H), 7.36–7.32 (m, 1H), 7.27–7.21 (m, 4H), 7.18–7.16 (m, 3H), 6.20 (s, 1H), 4.24–4.20 (m, 4H), 4.08 (d, *J* = 5.2 Hz, 2H), 1.28 (t, *J* = 7.2 Hz, 3H). ^13^C NMR (100 MHz, CDCl_3_) δ (ppm): 170.0, 169.8, 140.8, 139.4, 135.9, 131.0, 130.4, 129.1, 128.4, 127.3, 126.4, 126.1, 61.6, 41.7, 38.8, 14.2. HRMS (ESI) *m*/*z*: [M + Na]^+^ calcd for C_18_H_19_NO_3_Na 320.1263; found 320.1264.

*2-Benzyl-N,5-dimethylbenzamide (***3ia***)*: White solid (407.3 mg, 85%); m.p.: 145–147 °C. ^1^H NMR (400 MHz, CDCl_3_) δ (ppm): 7.26–7.22 (m, 2H), 7.16–7.13 (m, 5H), 7.10–7.09 (m, 1H), 5.58 (s, 1H), 4.10 (s, 2H), 2.82 (d, *J* = 5.2 Hz, 3H), 2.31 (s, 3H). ^13^C NMR (100 MHz, CDCl_3_) δ (ppm): 170.9, 141.2, 136.7, 136.0, 135.7, 130.8, 130.6, 128.9, 128.4, 127.8, 126.0, 38.5, 26.5, 20.9. HRMS (ESI) *m*/*z*: [M + H]^+^ calcd for C_16_H_18_NO 240.1388; found 240.1389.

*2-Benzyl-5-methoxy-N-methylbenzamide (***3ja***)*: White solid (423.8 mg, 83%); m.p.: 82–83 °C. ^1^H NMR (400 MHz, CDCl_3_) δ (ppm): 7.26–7.23 (m, 2H), 7.17–7.11 (m, 4H), 6.90–6.85 (m, 2H), 5.63 (s, 1H), 4.07 (s, 2H), 3.77 (s, 3H), 2.81 (d, *J* = 4.8 Hz, 3H). ^13^C NMR (100 MHz, CDCl_3_) δ (ppm): 170.5, 157.9, 141.3, 137.7, 132.1, 130.6, 128.8, 128.4, 126.1, 115.5, 112.7, 55.4, 38.2, 26.5. HRMS (ESI) *m*/*z*: [M + Na]^+^ calcd for C_16_H_17_NO_2_Na 278.1157; found 278.1158.

*Methyl 4-benzyl-3-(methylcarbamoyl)benzoate (***3ka***)*: White solid (79.3 mg, 14%); m.p.: 135–137 °C. ^1^H NMR (400 MHz, CDCl_3_) δ (ppm): 7.86 (s, 1H), 7.79 (d, *J* = 8.0 Hz, 1H), 7.31 (d, *J* = 8.0 Hz, 1H), 7.22 (t, *J* = 7.2 Hz, 2H), 7.16–7.11 (m, 3H), 6.02 (s, 1H), 4.12 (s, 2H), 3.85 (s, 3H), 2.77 (d, *J* = 4.8 Hz, 3H). ^13^C NMR (100 MHz, CDCl_3_) δ (ppm): 169.9, 166.4, 140.9, 140.2, 139.3, 131.8, 131.2, 128.9, 128.5, 127.5, 127.2, 126.3, 52.3, 38.8, 26.5. HRMS (ESI) *m*/*z*: [M + H]^+^ calcd for C_17_H_18_NO_3_ 284.1287; found 284.1285.

*2-Benzyl-5-fluoro-N-methylbenzamide (***3la***)*: White solid (379.5 mg, 78%); m.p.: 111–113 °C. ^1^H NMR (400 MHz, CDCl_3_) δ (ppm): 7.27–7.24 (m, 2H), 7.19–7.12 (m, 4H), 7.06–7.00 (m, 2H), 5.68 (s, 1H), 4.10 (s, 2H), 2.81 (d, *J* = 4.8 Hz, 3H). ^13^C NMR (100 MHz, CDCl_3_) δ (ppm): 169.4 (d, *J* = 2.0 Hz), 160.9(d, *J* = 245.0 Hz), 140.6, 138.2 (d, *J* = 6.3 Hz), 134.7 (d, *J* = 3.5 Hz), 132.6 (d, *J* = 7.7 Hz), 128.9, 128.5, 126.3, 116.7 (d, *J* = 20.7 Hz), 114.3 (d, *J* = 22.7 Hz), 38.2, 26.6. HRMS (ESI) *m*/*z*: [M + Na]^+^ calcd for C_15_H_14_FNONa 266.0957; found 266.0956.

*2-Benzyl-5-chloro-N-methylbenzamide (***3ma***)*: White solid (161.0 mg, 31%); m.p.: 124–126 °C. ^1^H NMR (400 MHz, CDCl_3_) δ (ppm): 7.31–7.23 (m, 4H), 7.19–7.11 (m, 4H), 5.74 (s, 1H), 4.09 (s, 2H), 2.81 (d, *J* = 4.8 Hz, 3H). ^13^C NMR (100 MHz, CDCl_3_) δ (ppm): 169.3, 140.4, 138.2, 137.6, 132.3, 132.1, 130.0, 129.0, 128.6, 127.3, 126.4, 38.4, 26.6. HRMS (ESI) *m*/*z*: [M + H]^+^ calcd for C_15_H_14_ClNONa 282.0662; found 282.0661.

*2-Benzyl-3-methoxy-N-methylbenzamide (***3na***)*: White solid (398.3 mg, 78%); m.p.: 115–116 °C. ^1^H NMR (400 MHz, CDCl_3_) δ (ppm): 7.24–7.08 (m, 6H), 6.92 (t, *J* = 7.7 Hz, 2H), 5.65 (s, 1H), 4.15 (s, 2H), 3.78 (s, 3H), 2.76 (d, *J* = 4.0 Hz, 3H). ^13^C NMR (100 MHz, CDCl_3_) δ (ppm): 170.7, 157.8, 141.3, 138.5, 128.5, 128.1, 127.6, 127.1, 125.6, 119.3, 112.1, 55.7, 31.9, 26.4. HRMS (ESI) *m*/*z*: [M + Na]^+^ calcd for C_16_H_17_NO_2_Na 278.1157; found 278.1158.

*2-Benzyl-N,3-dimethylbenzamide (***3oa***)*: White solid (215.4 mg, 45%); m.p.: 117–118 °C. ^1^H NMR (400 MHz, CDCl_3_) δ (ppm): 7.25–7.12 (m, 6H), 7.05 (d, *J* = 7.6 Hz, 2H), 5.63 (s, 1H), 4.17 (s, 2H), 2.77 (d, *J* = 5.2 Hz, 3H), 2.23 (s, 3H). ^13^C NMR (100 MHz, CDCl_3_) δ (ppm): 171.3, 140.4, 138.4, 138.3, 136.0, 132.0, 128.5, 128.2, 126.6, 125.9, 125.0, 35.6, 26.6, 20.2. HRMS (ESI) *m*/*z*: [M + Na]^+^ calcd for C_16_H_17_NONa 262.1208; found 262.1209.

*N-Methyl-2-(2-methylbenzyl)benzamide (***3ab***)*: White solid (392.5 mg, 82%); m.p.: 138–139 °C; ^1^H NMR (400 MHz, CDCl_3_) δ (ppm): 7.36 (d, *J* = 7.6 Hz, 1H), 7.30–7.25 (m, 1H), 7.22–7.08 (m, 4H), 7.01 (d, *J* = 7.6 Hz, 1H), 6.95 (d, *J* = 7.2 Hz, 1H), 5.78 (s, 1H), 4.13 (s, 2H), 2.83 (d, *J* = 4.8 Hz, 3H), 2.23 (s, 3H). ^13^C NMR (100 MHz, CDCl_3_) δ (ppm): 170.8, 138.8, 138.3, 136.9, 136.8, 130.4, 130.3, 130.0, 129.7, 127.1, 126.5, 126.2, 126.1, 36.4, 26.6, 19.8. HRMS (ESI) *m*/*z*: [M + H]^+^ calcd for C_16_H_18_NO 240.1388; found 240.1389.

*2-(2,6-Dimethylbenzyl)-N-methylbenzamide (***3ac***)*: White solid (304.0 mg, 60%); m.p.: 166–167 °C. ^1^H NMR (400 MHz, CDCl_3_) δ (ppm): 7.36–7.34 (m, 1H), 7.19–7.15 (m, 2H), 7.11–7.04 (m, 3H), 6.65–6.63 (m, 1H), 5.99 (s, 1H), 4.21 (s, 2H), 3.01 (d, *J* = 4.8 Hz, 3H), 2.18 (s, 6H). ^13^C NMR (100 MHz, CDCl_3_) δ (ppm): 170.9, 138.1, 137.4, 136.6, 136.5, 130.1, 128.2, 127.7, 126.6, 126.5, 125.9, 32.2, 26.8, 20.2. HRMS (ESI) *m*/*z*: [M + H]^+^ calcd for C_17_H_20_NO 254.1545; found 254.1544.

*N-Methyl-2-(4-methylbenzyl)benzamide (***3ad***)*: White solid (397.3 mg, 83%); m.p.: 129–130 °C. ^1^H NMR (400 MHz, CDCl_3_) δ (ppm): 7.34–7.30 (m, 2H), 7.22–7.18 (m, 2H), 7.07–7.03 (m, 4H), 5.66 (s, 1H), 4.10 (s, 2H), 2.84 (d, *J* = 5.2 Hz, 3H), 2.29 (s, 3H). ^13^C NMR (100 MHz, CDCl_3_) δ (ppm): 170.8, 139.1, 137.8, 136.8, 135.6, 130.8, 129.9, 129.2, 128.9, 127.2, 126.3, 38.5, 26.5, 21.0. HRMS (ESI) *m*/*z*: [M + H]^+^ calcd for C_16_H_18_NO 240.1388; found 240.1389.

*2-(4-Fluorobenzyl)-N-methylbenzamide (***3ae***)*: White solid (379.5 mg, 78%); m.p.: 125–126 °C. ^1^H NMR (400 MHz, CDCl_3_) δ (ppm): 7.35–7.32 (m, 2H), 7.23–7.18 (m, 2H), 7.14–7.11 (m, 2H), 6.95–6.90 (m, 2H), 5.70 (s, 1H), 4.12 (s, 2H), 2.85 (d, *J* = 4.8 Hz, 3H). ^13^C NMR (100 MHz, CDCl_3_) δ (ppm): 170.7, 161.4 (d, *J* = 242.5 Hz), 139.1, 136.6, 136.6, 130.8, 130.5 (d, *J* = 7.8 Hz), 130.0, 127.1, 126.4, 115.1 (d, *J* = 21.0 Hz), 38.1, 26.6. HRMS (ESI) *m*/*z*: [M + H]^+^ calcd for C_15_H_15_FNO 244.1138; found 244.1137.

*2-(4-Chlorobenzyl)-N-methylbenzamide (***3af***)*: White solid (368.8 mg, 71%); m.p.: 156–157 °C. ^1^H NMR (400 MHz, CDCl_3_) δ (ppm): 7.35–7.32 (m, 2H), 7.24–7.18 (m, 4H), 7.11–7.09 (m, 2H), 5.69 (s, 1H), 4.12 (s, 2H), 2.85 (d, *J* = 4.8 Hz, 3H). ^13^C NMR (100 MHz, CDCl_3_) δ (ppm): 170.6, 139.5, 138.8, 136.6, 131.9, 130.9, 130.4, 130.1, 128.5, 127.1, 126.6, 38.3, 26.6. HRMS (ESI) *m*/*z*: [M + H]^+^ calcd for C_15_H_14_ClNONa 282.0662; found 282.0663.

*2-(4-Bromobenzyl)-N-methylbenzamide (***3ag***)*: White solid (366.6 mg, 60%); m.p.: 158–159 °C. ^1^H NMR (400 MHz, CDCl_3_) δ (ppm): 7.37–7.32 (m, 4H), 7.24–7.18 (m, 2H), 7.06–7.04 (m, 2H), 5.66 (s, 1H), 4.11 (s, 2H), 2.86 (d, *J* = 4.8 Hz, 3H). ^13^C NMR (100 MHz, CDCl_3_) δ (ppm): 170.6, 140.0, 138.7, 136.6, 131.5, 130.9, 130.9, 130.2, 127.1, 126.6, 120.0, 38.4, 26.6. HRMS (ESI) *m*/*z*: [M + H]^+^ calcd for C_15_H_15_BrNO 304.0337; found 304.0338.

*N-Methyl-2-(4-(trifluoromethyl)benzyl)benzamide (***3ah***)*: White solid (134.9 mg, 23%; 247.1 mg, 42% with 10% cat.); m.p.: 121–122 °C. ^1^H NMR (400 MHz, CDCl_3_) δ (ppm): 7.49 (d, *J* = 8.0 Hz, 2H), 7.37–7.33 (m, 2H), 7.29 (d, *J* = 8.0 Hz, 2H), 7.26–7.19 (m, 2H), 5.73 (s, 1H), 4.22 (s, 2H), 2.85 (d, *J* = 4.8 Hz, 3H). ^13^C NMR (100 MHz, CDCl_3_) δ (ppm): 170.6, 145.2, 138.4, 136.6, 131.0, 130.2, 129.4, 128.4 (q, *J* = 32.1 Hz), 127.1, 126.7, 125.2 (q, *J* = 3.7Hz),124.4 (q, *J* = 270.1 Hz),38.7, 26.6. HRMS (ESI) *m*/*z*: [M + H]^+^ calcd for C_16_H_15_F_3_NO 294.1106; found 294.1107.

*Methyl 4-(2-(methylcarbamoyl)benzyl)benzoate (***3ai***)*: White solid (119.0 mg, 21%; 203.8 mg, 36% with 10% cat.); m.p.: 139–141 °C. ^1^H NMR (400 MHz, CDCl_3_) δ (ppm): 7.91 (d, *J* = 8.3 Hz, 2H), 7.36–7.32 (m, 2H), 7.25–7.19 (m, 4H), 5.74 (s, 1H), 4.22 (s, 2H), 3.87 (s, 3H), 2.84 (d, *J* = 5.2 Hz, 3H). ^13^C NMR (100 MHz, CDCl_3_) δ (ppm): 170.6, 167.1, 146.4, 138.3, 136.6, 130.9, 130.1, 129.6, 129.1, 128.0, 127.1, 126.6, 52.0, 38.9, 26.6. HRMS (ESI) *m*/*z*: [M + Na]^+^ calcd for C_17_H_17_NO_3_Na 306.1106; found 306.1107.

*2-(4-Cyanobenzyl)-N-methylbenzamide (***3aj***)*: White solid (70.1 mg, 14% with 10 mol% cat.); m.p.: 141–143 °C. ^1^H NMR (400 MHz, CDCl_3_) δ (ppm): 7.51 (d, *J* = 8.0 Hz, 2H), 7.38–7.34 (m, 2H), 7.30–7.23 (m, 3H), 7.20–7.18 (m, 1H), 5.86 (s, 1H), 4.22 (s, 2H), 2.86 (d, *J* = 4.8 Hz, 3H). ^13^C NMR (100 MHz, CDCl_3_) δ (ppm): 170.3, 146.8, 137.9, 136.5, 132.1, 131.1, 130.3, 129.8, 127.2, 126.9, 119.1, 109.8, 39.0, 26.6. HRMS (ESI) *m*/*z*: [M + H]^+^ calcd for C_16_H_15_N_2_O 251.1184; found 251.1183.

## 4. Conclusions

In summary, an efficient nickel-catalyzed, manganese-mediated denitrogenative cross-electrophile coupling of benzotriazinones with benzyl chlorides has been effected by LAG ball-milling with 1.5 μL/mg DMF and 1.0 equiv. CaCl_2_ as additives. The liquid additive DMF appeared to serve as both the assisting liquid for grinding and promotor for the nickel-catalyzed, manganese-mediated process under mechanochemical conditions. Large electronic effects were observed from both benzotriazinone and benzyl counterparts while the steric hindrance could be tolerated to some extent, in particular with respect to N-alkyl group of benzotriazinones and aryl ring of benzyl chlorides, although no reaction was observed for sec-benzyl chlorides. An electron-withdrawing substituent on benzotriazinones or benzyl chlorides could retard the XEC reaction seriously. Based on the catalytic cycle, a tentative explanation for these substrate structural effects has been proposed. This protocol provides a new access to N-alkyl-2-benzylbenzamides, i.e., diarylmethanes bearing an ortho-carbamoyl aryl group, in good yields if not containing additional electron-withdrawing groups.

## Figures and Tables

**Table 1:** Effects of salt additives ^a^.

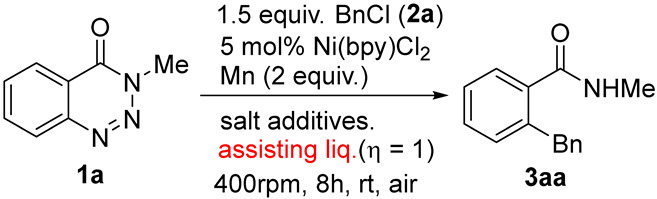			
Entry	Assisting Liquid	Salt (equiv.)	Yield (%) ^b^
1	DMA	/	14
2	DMF	/	26
3	CH_3_CN	/	trace
4	THF	/	trace
5	Diox.	/	trace
6	DMF	MgSO_4_ (1)	50
7	DMF	Na_2_SO_4_ (1)	30
8	DMF	NaCl (1)	27
9	DMF	LiCl (1)	66
10	DMF	LiBr (1)	28
11	DMF	CaCl_2_ (1)	75
12	DMF	MgCl_2_ (1)	65
13	DMF	ZnCl_2_ (1)	58
14	DMF	BaCl_2_ (1)	36
15	DMF	LiCl (2)	68
16	DMF	CaCl_2_ (0.5)	50
17	DMF	CaCl_2_ (1.5)	73

^a^ Reaction run in air at 2 mmol scale with respect to **1a** using 1.5 equiv. **2a**, 5 mol % Ni(bpy)Cl_2_, 2.0 equiv. Mn powders, 16 stainless-steel balls (ø = 7 mm) in 50 mL jar at 400 rpm in the presence of assisting liquid (η = 1) and salt additive. ^b^ Isolated yields.

**Table 2:** Optimization of the XEC-based denitrogenative benzylation of benzotriazinones ^a^.

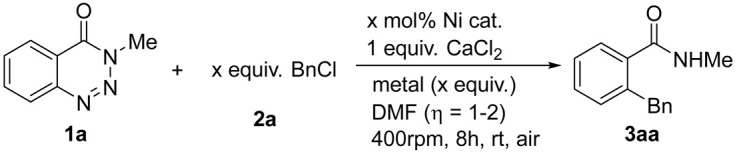					
Entry	Cat. (mol%)	η (DMF)	Red. (equiv.)	Equiv. (BnCl)	Yield (%) ^b^
1	Ni(bpy)Br_2_ (5)	1	Mn (2)	1.5	73
2	Ni(bpy)Cl_2_ (3)	1	Mn (2)	1.5	49
3	Ni(bpy)Cl_2_ (1)	1	Mn (2)	1.5	trace
4	NiCl_2_/bpy (5)	1	Mn (2)	1.5	68
5	Ni(acac)_2_/bpy (5)	1	Mn (2)	1.5	65
6	Ni(OAc)_2_/bpy (5)	1	Mn (2)	1.5	63
7	NiCl_2_/dtbbpy (5)	1	Mn (2)	1.5	42
8	NiCl_2_/phen (5)	1	Mn (2)	1.5	30
9	NiCl_2_/tpy (5)	1	Mn (2)	1.5	trace
10	Ni(bpy)Cl_2_ (5)	0	Mn (2)	1.5	trace
11	Ni(bpy)Cl_2_ (5)	0.5	Mn (2)	1.5	8
12	Ni(bpy)Cl_2_ (5)	1.5	Mn (2)	1.5	72
13	Ni(bpy)Cl_2_ (5)	2	Mn (2)	1.5	74
14	Ni(bpy)Cl_2_ (5)	1	Al (2)	1.5	NR
15	Ni(bpy)Cl_2_ (5)	1	Zn (2)	1.5	18
16	Ni(bpy)Cl_2_ (5)	1	Mn (1.5)	1.5	64
17	Ni(bpy)Cl_2_ (5)	1	Mn (2.5)	1.5	76
18	Ni(bpy)Cl_2_ (5)	1	Mn (2)	1.3	60
19	Ni(bpy)Cl_2_ (5)	1	Mn (2)	2.0	71 ^c^
20	Ni(bpy)Cl_2_ (5)	1.5	Mn (2)	2.0	84
21	Ni(bpy)Cl_2_ (5)	2	Mn (2)	2.0	82
22	Ni(bpy)Cl_2_ (5)	1.5	Mn (2)	2.5	85
23	Ni(bpy)Cl_2_ (5)	2	Mn (2)	2.5	84
24	Ni(bpy)Cl_2_ (10)	1.5	Mn (2)	2.0	82 ^d^
25	Ni(bpy)Cl_2_ (5)	1.5	Mn (2)	2.0	73 ^e^
26	Ni(bpy)Cl_2_ (5)	1.5	Mn (2)	2.0	61 ^f^

^a^ Reaction run in air at 2 mmol scale with respect to **1a** using 1.5–2.5 equiv. **2a**, 1–10 mol % Ni cat., 1.5–2.5 equiv. metal reductant, 1.0 equiv. CaCl_2_, 16 stainless-steel balls (ø = 7 mm) in 50 mL jar at 400 rpm in the presence of DMF (η = 1–2) assisting liquid. ^b^ Isolated yields. ^c^ About 20% **1a** recovered. ^d^ Completed in 5 h. ^e^ At 300 rpm. ^f^ With 8 stainless-steel balls (ø = 7 mm).

## Data Availability

Data are reported in the paper and Supplementary Materials.

## Supplementary Materials

The following supporting information can be downloaded at: https://www.mdpi.com/article/10.3390/molecules30051060/s1, Copies of ^1^H & ^13^C NMR spectra of products and HRMS for all new compounds.
