# Supplementary material for: Liquid-Assisted Grinding Enables Efficient Ni-Catalyzed, Mn-Mediated Denitrogenative Cross-Electrophile Coupling of Benzotriazinones with Benzyl Chlorides
Source: Molecules. 2025 Feb 26;30(5):1060. doi: 10.3390/molecules30051060 (PMC11901950; doi:10.3390/molecules30051060)
Supplement: Supplementary file 1 [file molecules-30-01060-s001.zip › molecules-3472962-supplementary.pdf]

Supplementary Materials

for

Liquid-assisted grinding enables efficient Ni-catalyzed, Mn-mediated denitrogenative cross-electrophile coupling of benzotriazinones with benzyl chlorides

Xuanxuan Zhang, Yingying Hong and Gang Zou*

2-benzyl-N-methylbenzamide(3aa)

^{1}H NMR (400 MHz, $CDCl_3$)

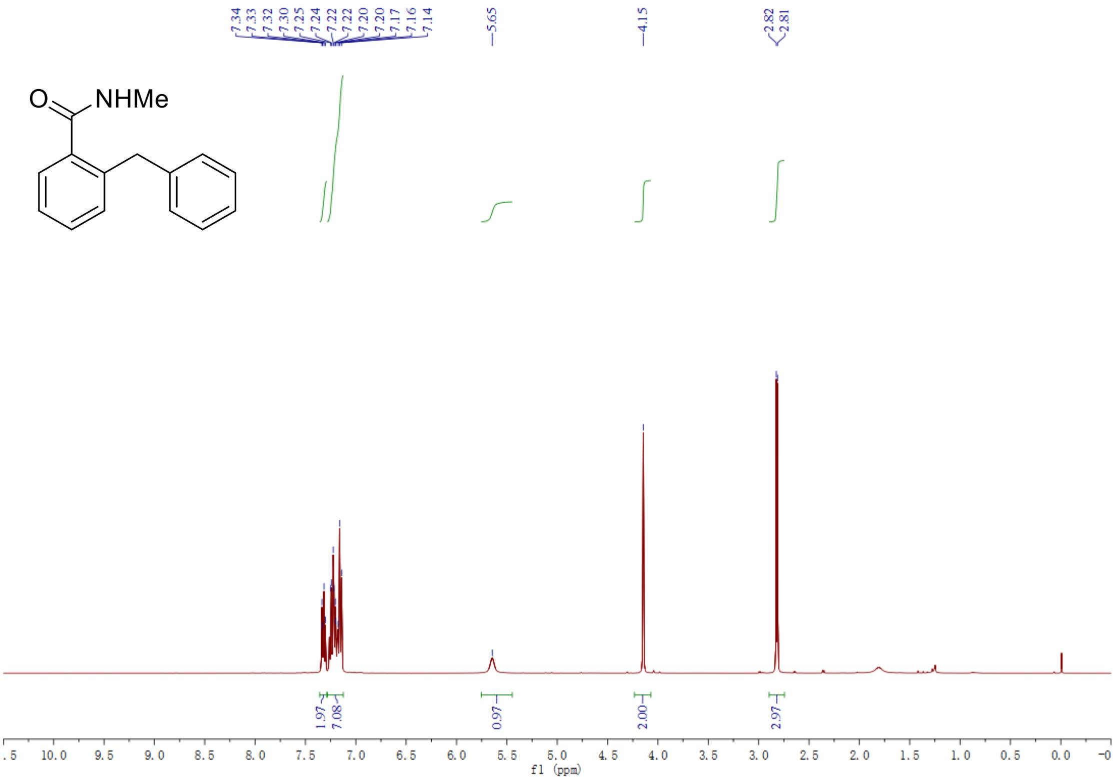

^{13}C NMR (100 MHz, $CDCl_3$)

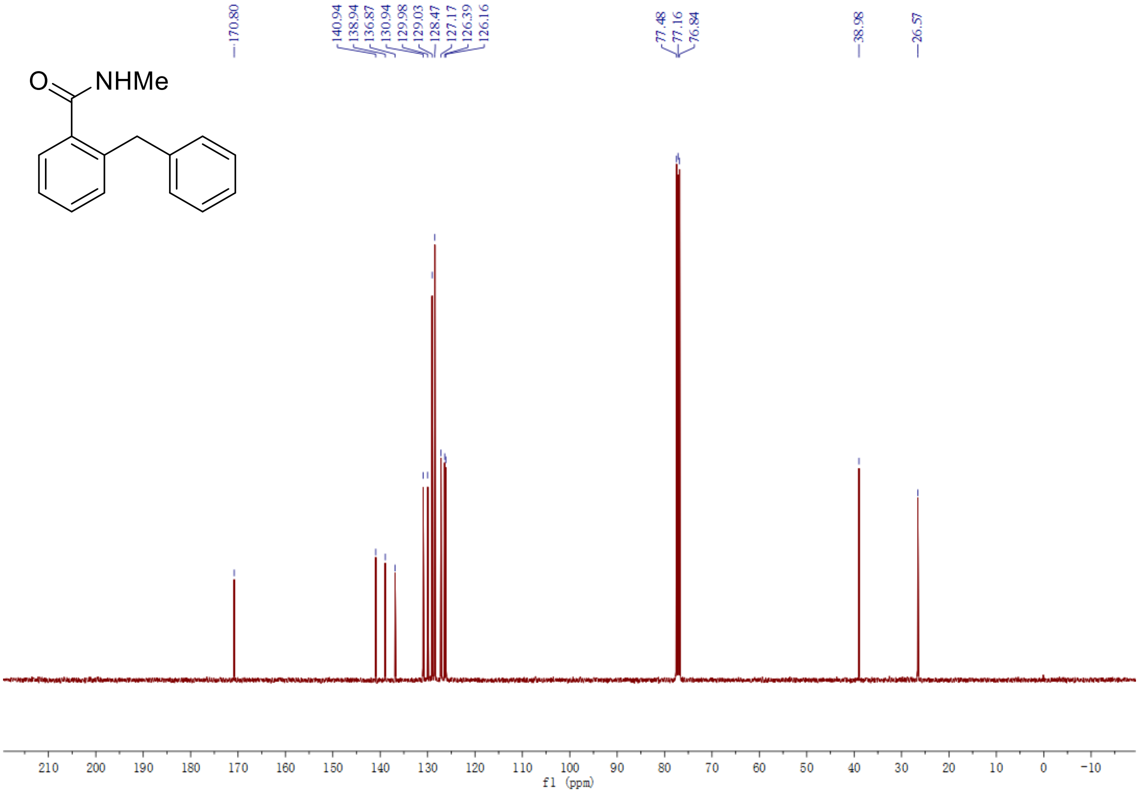

2-benzyl-N-propylbenzamide(3ba)

^{1}H NMR (400 MHz, $CDCl_3$)

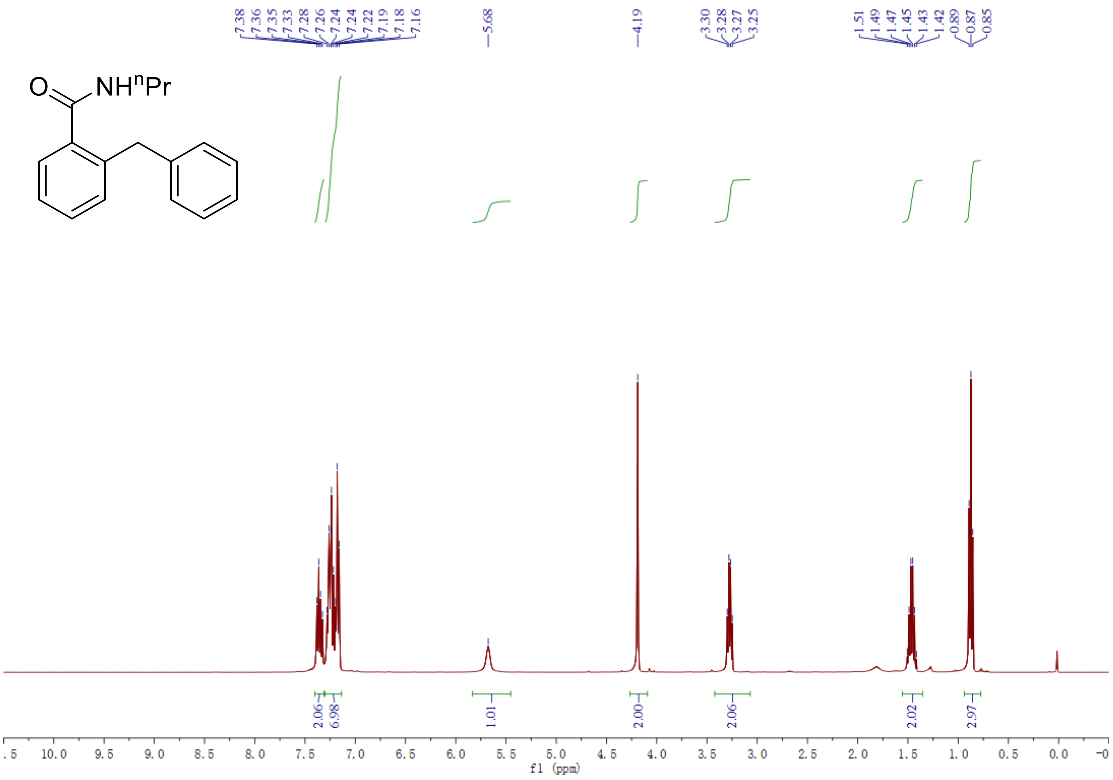

^{13}C NMR (100 MHz, $CDCl_3$)

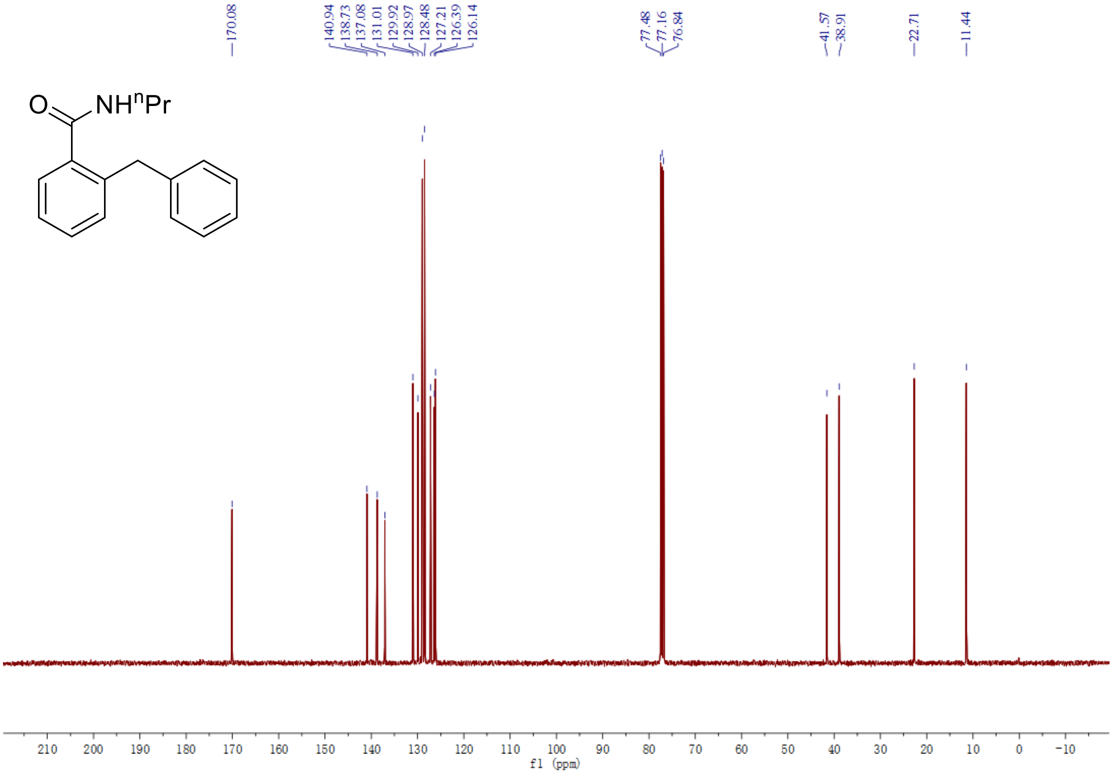

2-benzyl-N-isopropylbenzamide(3ca)

^{1}H NMR (400 MHz, $CDCl_3$)

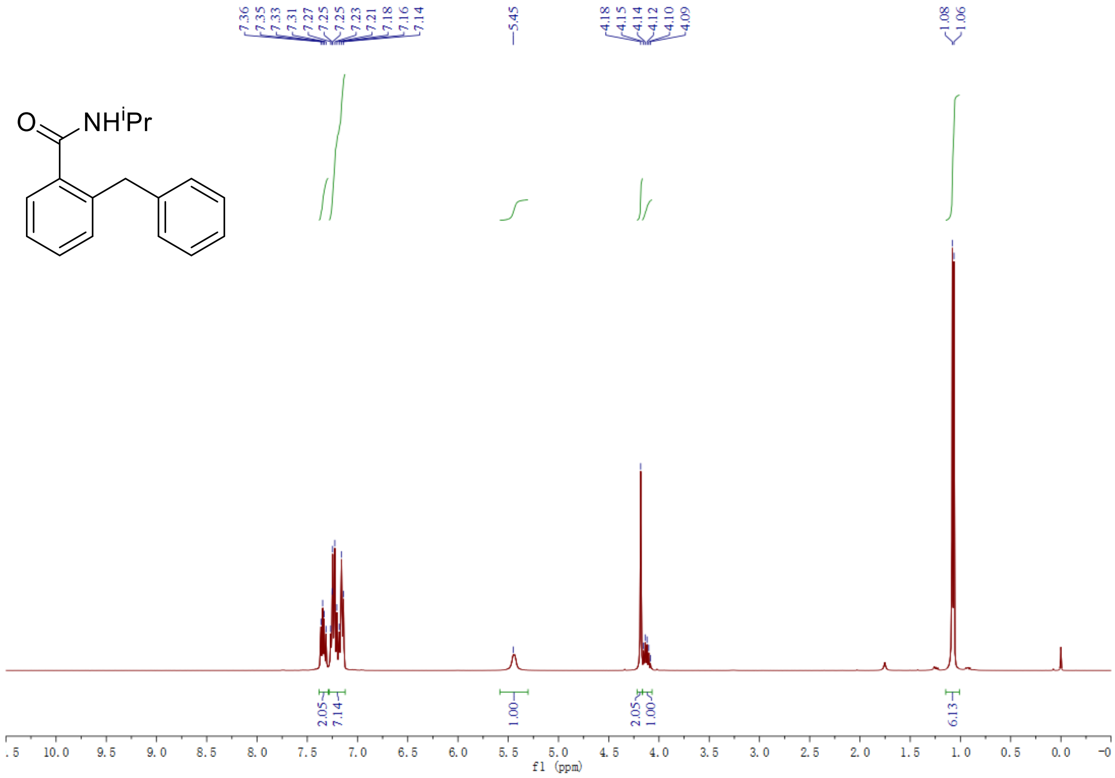

^{13}C NMR (100 MHz, $CDCl_3$)

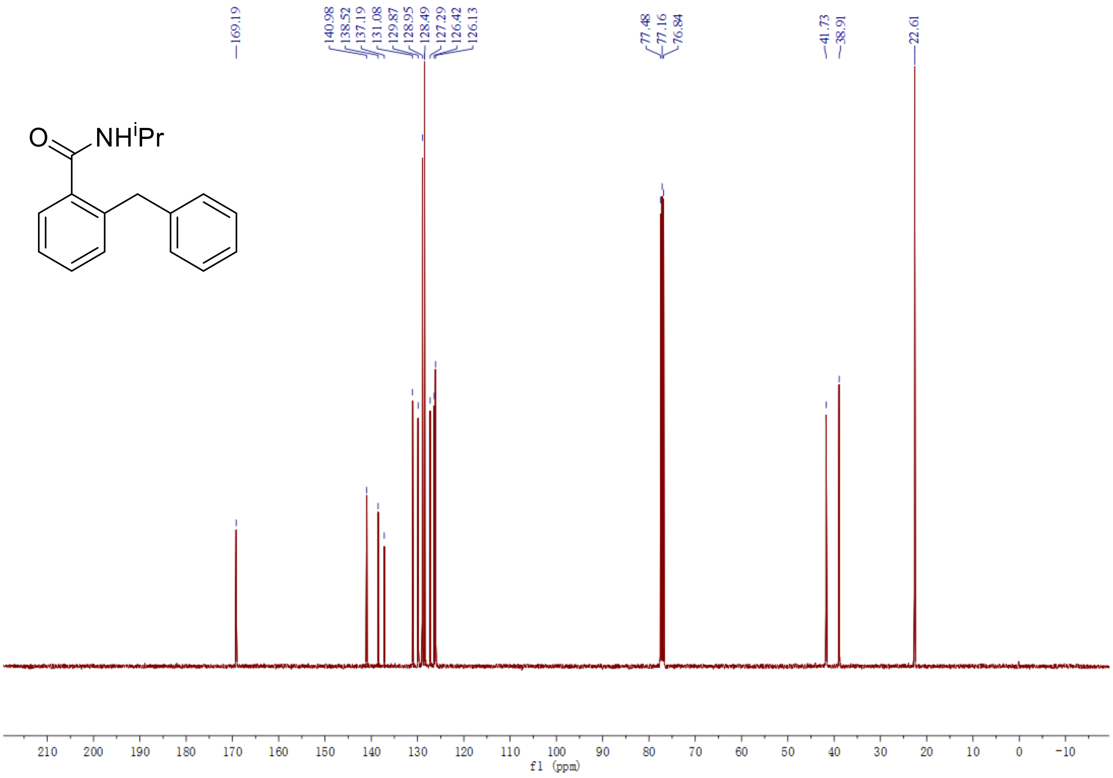

2-benzyl-N-cyclohexylbenzamide(3da)

^{1}H NMR (400 MHz, $CDCl_3$)

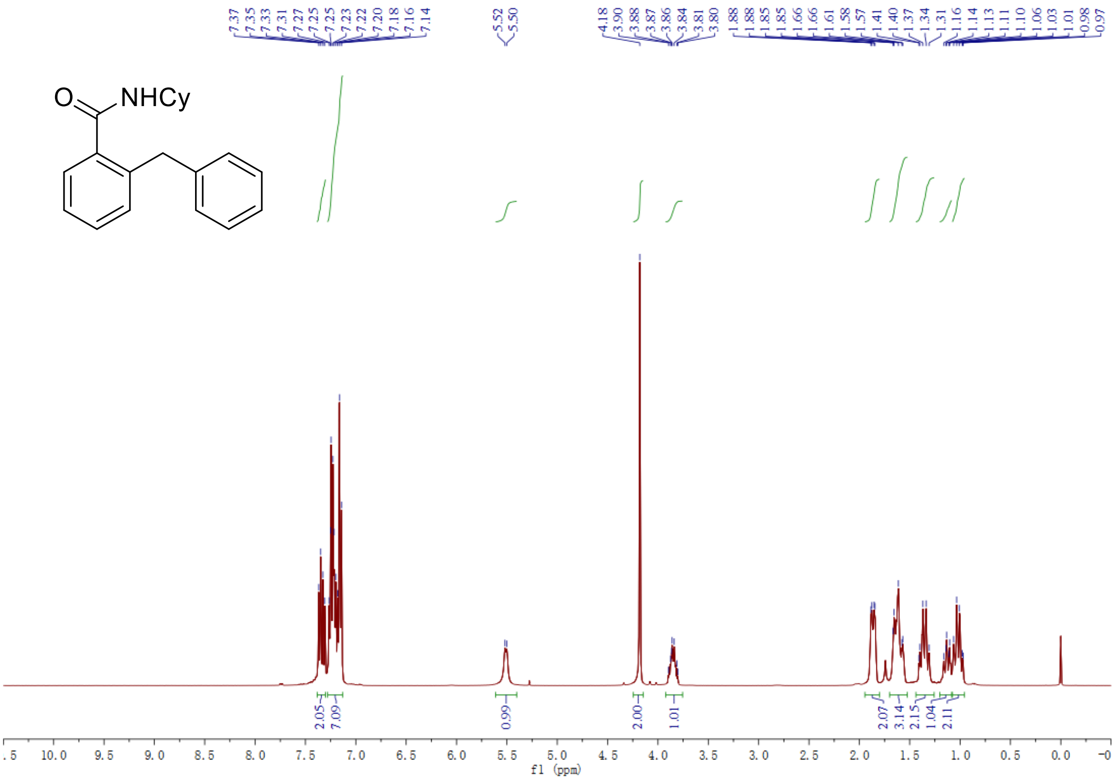

^{13}C NMR (100 MHz, $CDCl_3$)

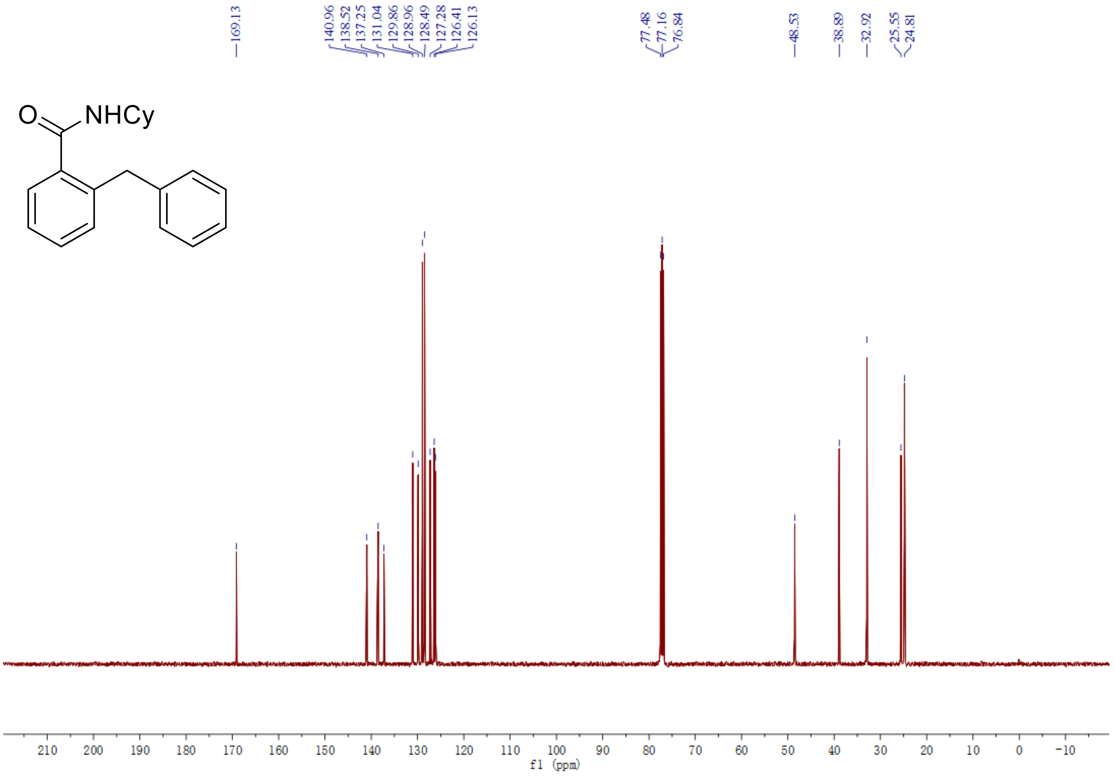

2-benzyl-N-(2-methoxyethyl)benzamide(3ea)

^{1}H NMR (400 MHz, $CDCl_3$)

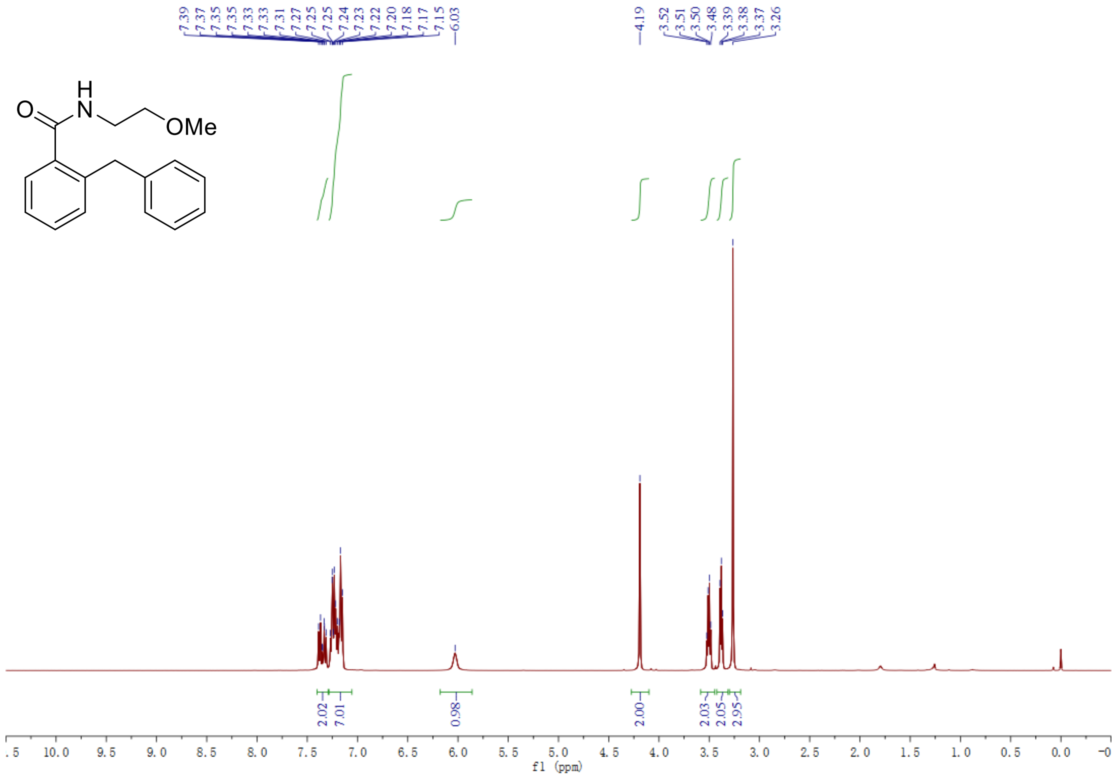

^{13}C NMR (100 MHz, $CDCl_3$)

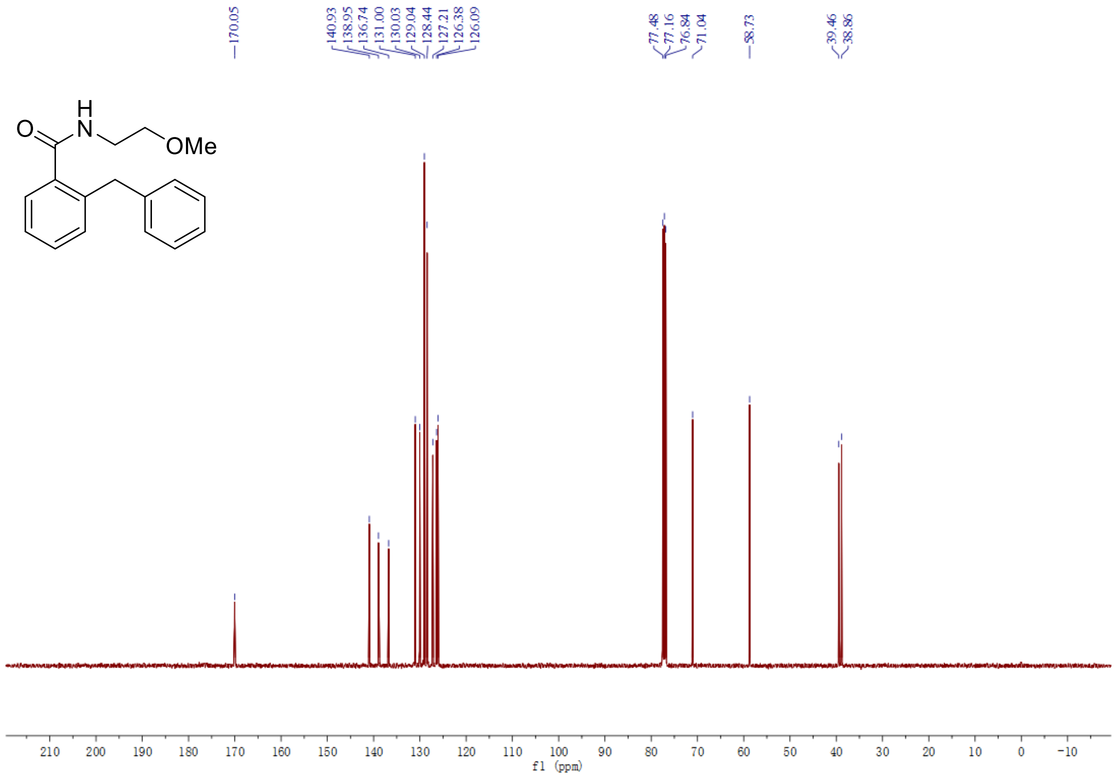

ethyl (2-benzylbenzoyl)glycinate(3fa)

^{1}H NMR (400 MHz, $CDCl_3$)

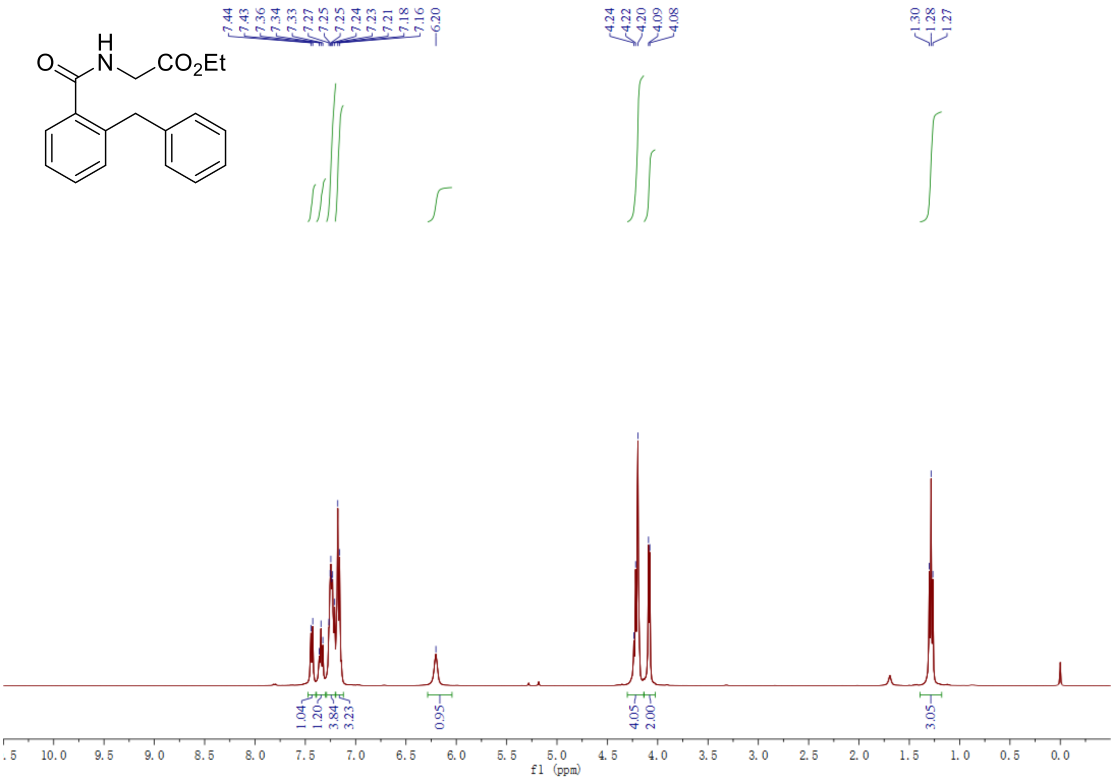

^{13}C NMR (100 MHz, $CDCl_3$)

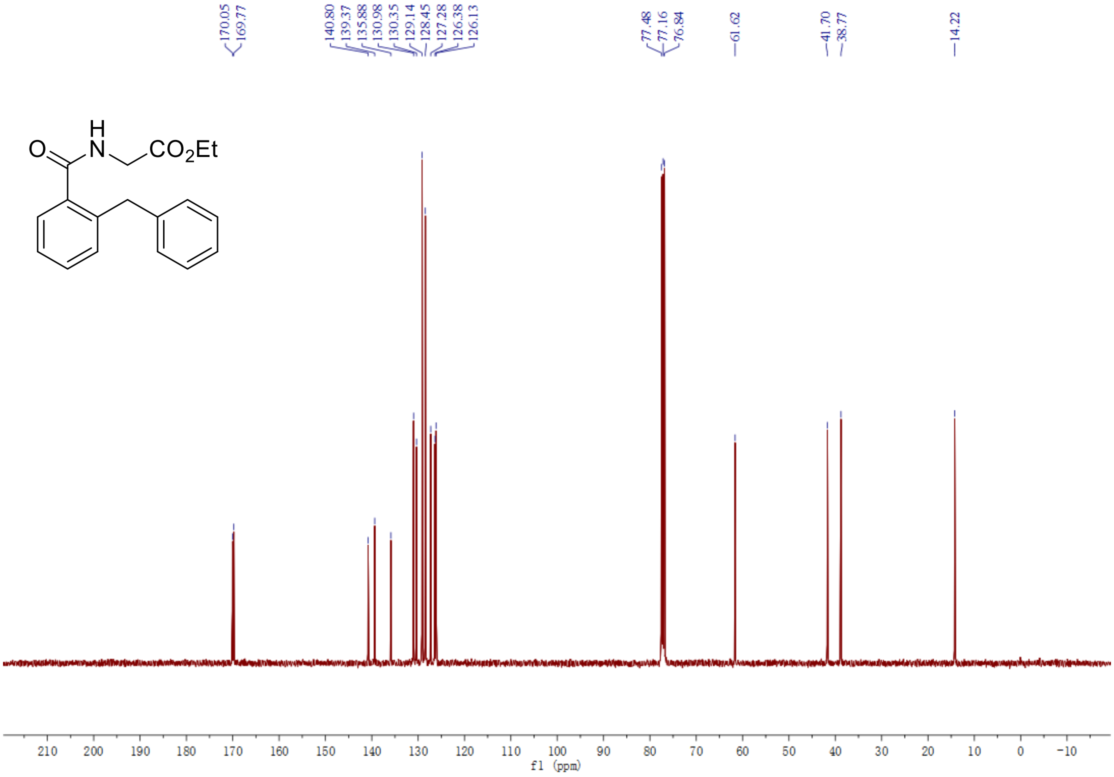

2-benzyl-N,5-dimethylbenzamide(3ia)

^{1}H NMR (400 MHz, $CDCl_3$)

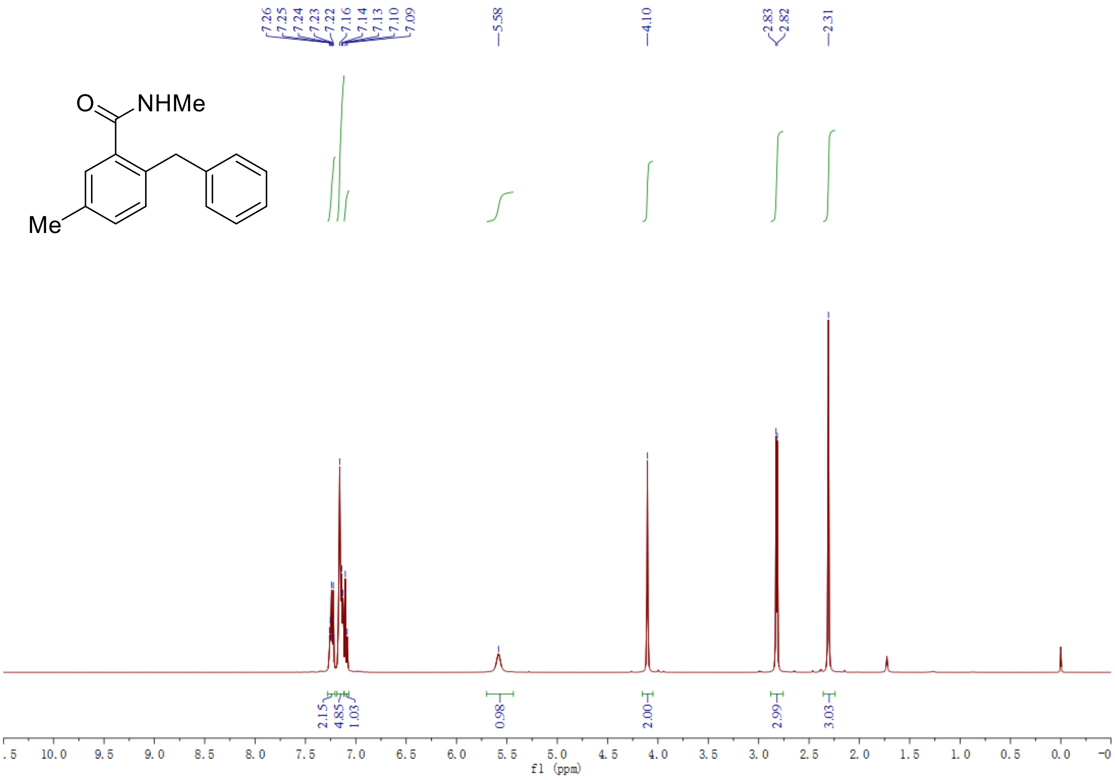

^{13}C NMR (100 MHz, $CDCl_3$)

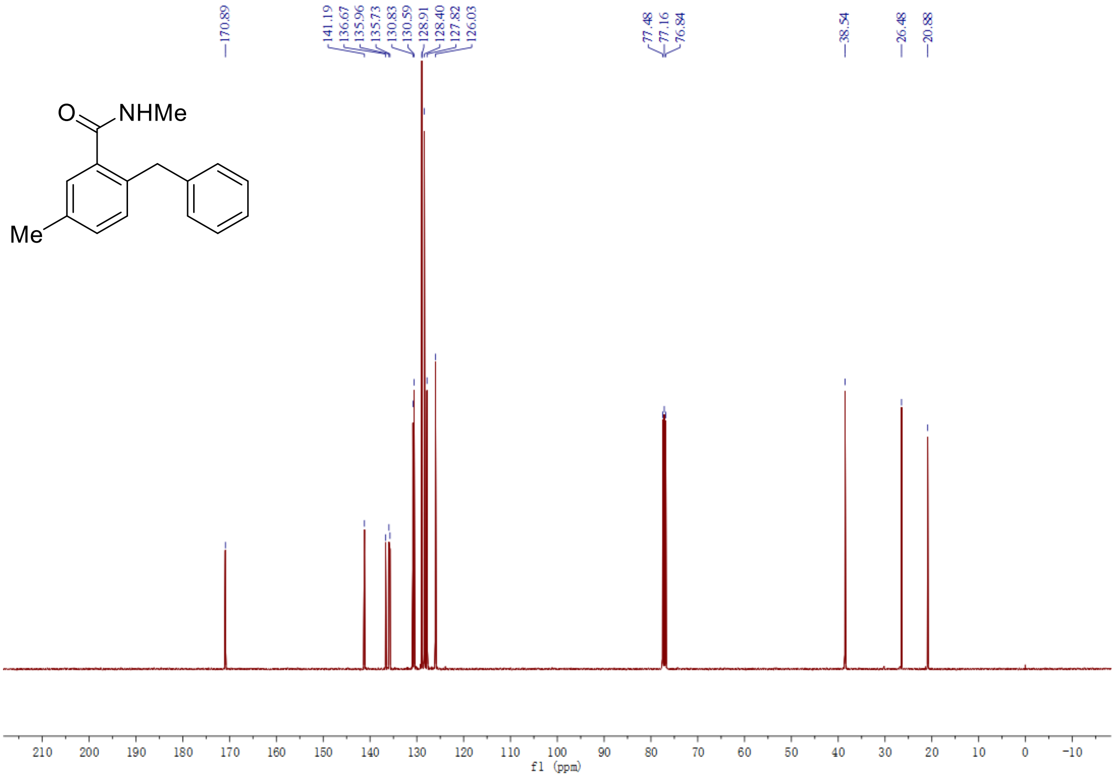

2-benzyl-5-methoxy-N-methylbenzamide(3ja)

^{1}H NMR (400 MHz, $CDCl_3$)

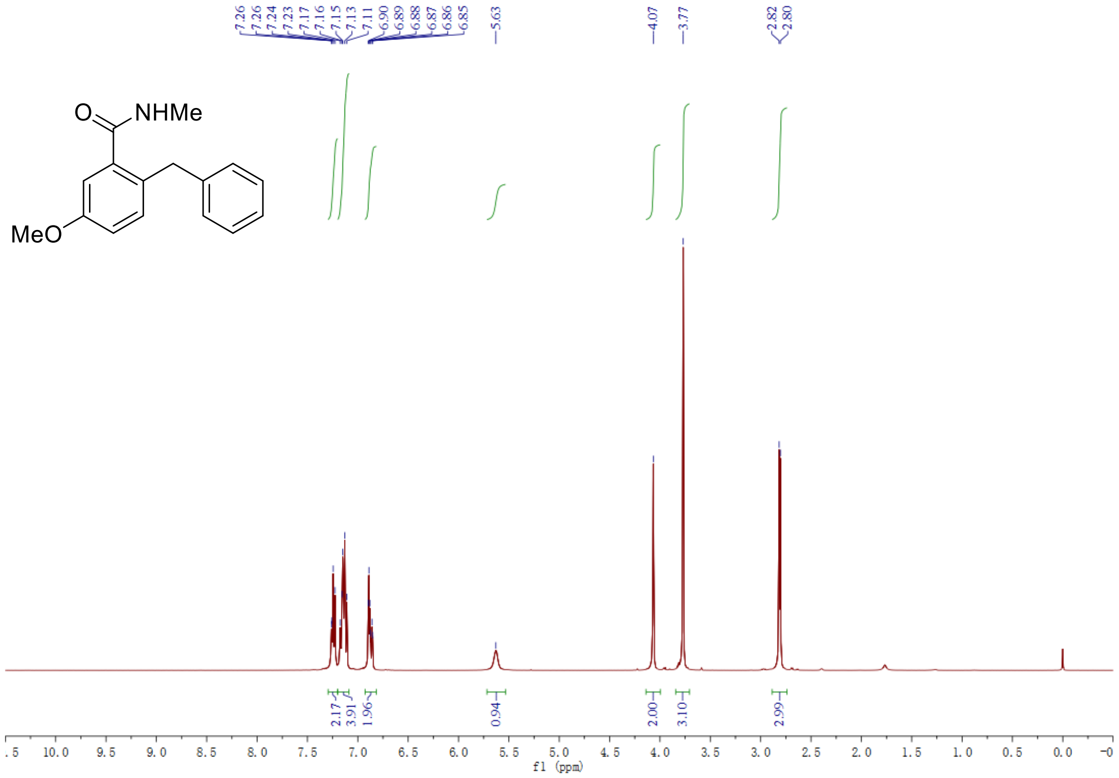

^{13}C NMR (100 MHz, $CDCl_3$)

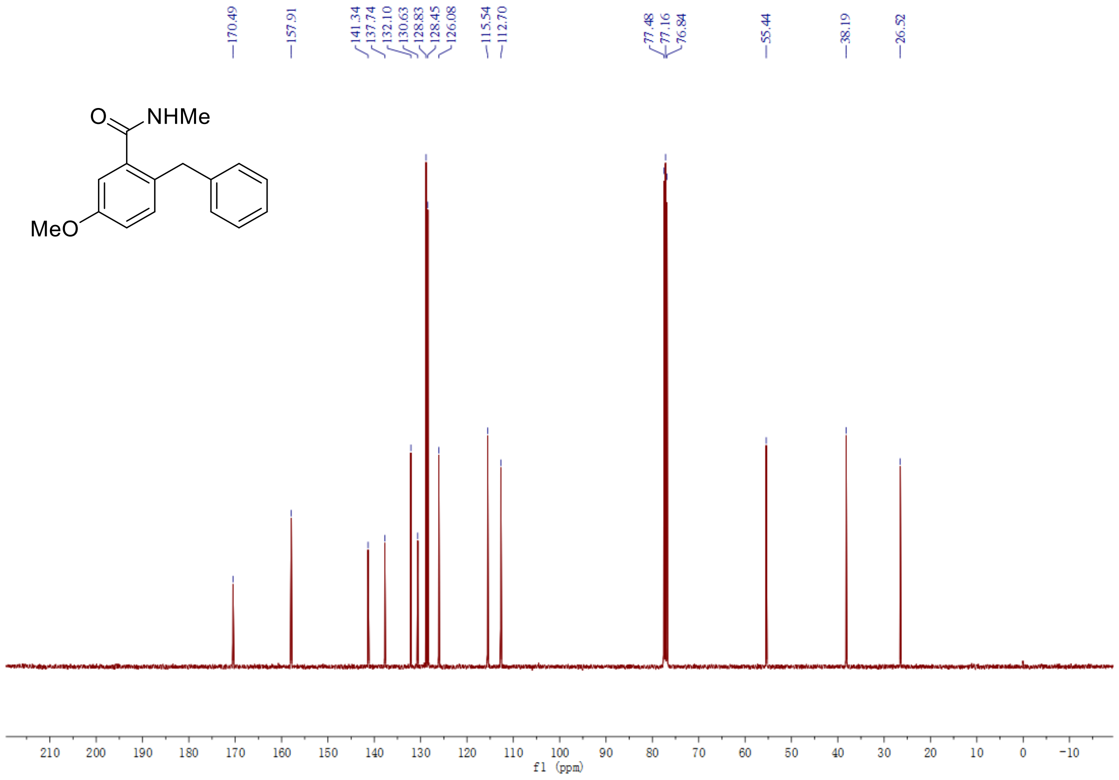

methyl 4-benzyl-3-(methylcarbamoyl)benzoate (3ka)

^{1}H NMR (400 MHz, $CDCl_3$)

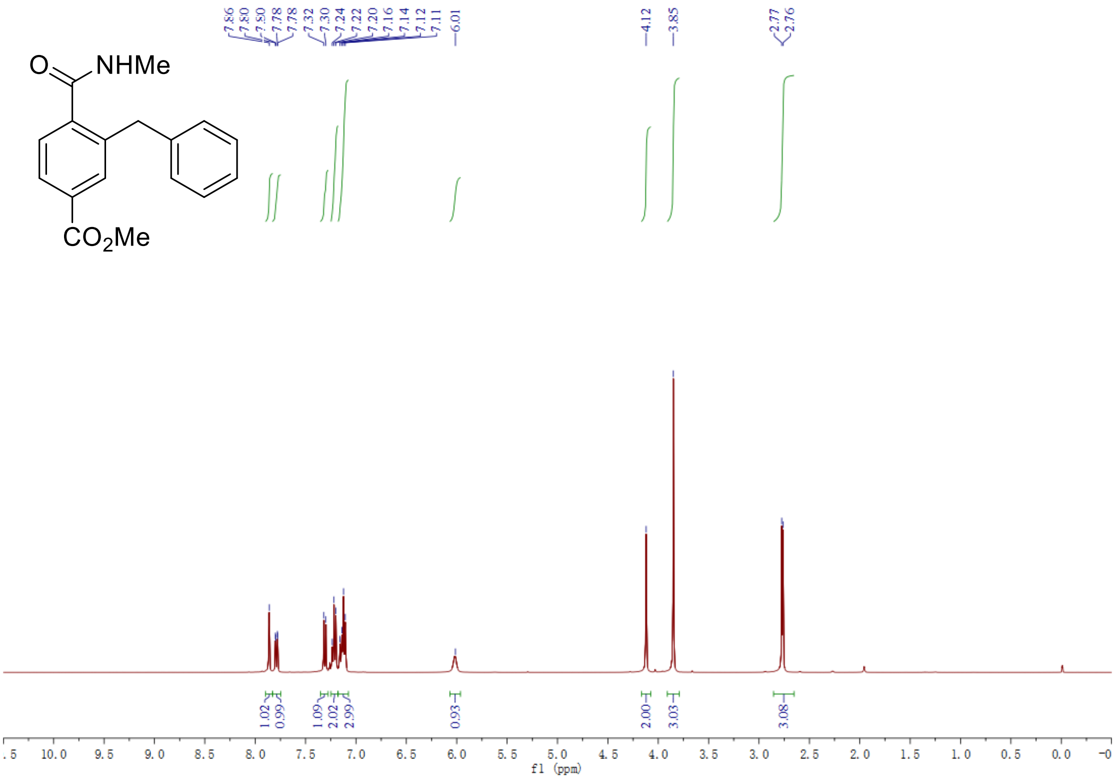

^{13}C NMR (100 MHz, $CDCl_3$)

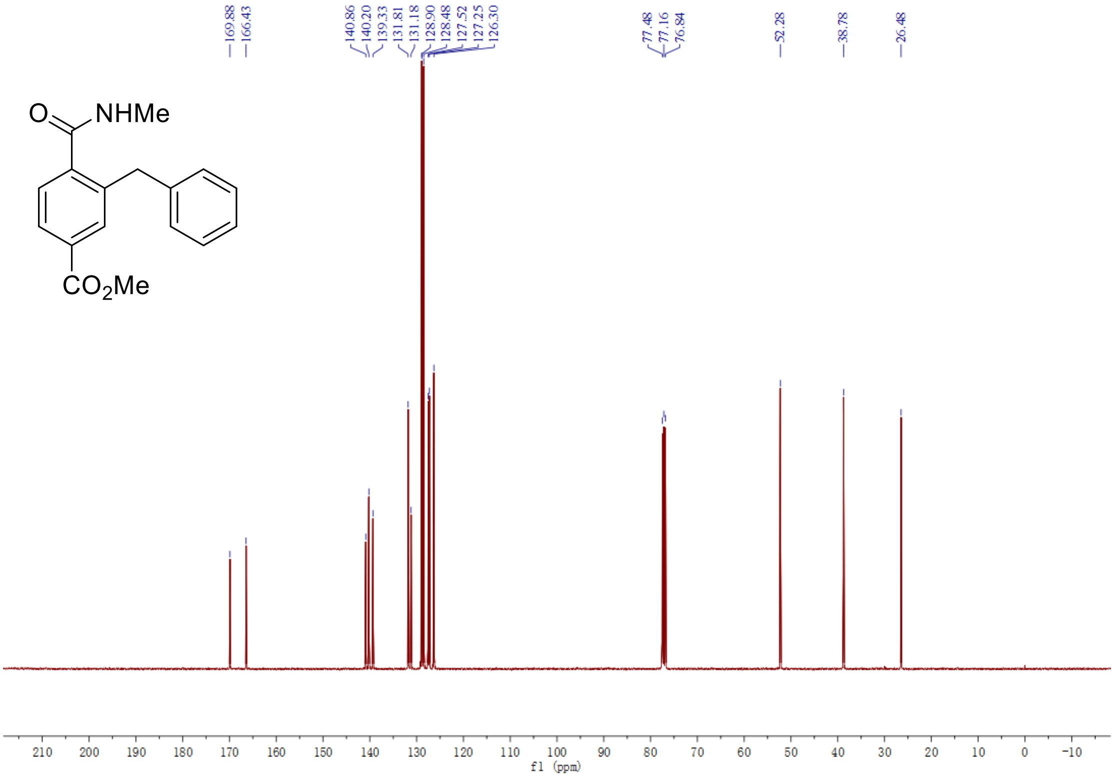

2-benzyl-5-fluoro-N-methylbenzamide(3la)

^{1}H NMR (400 MHz, $CDCl_3$)

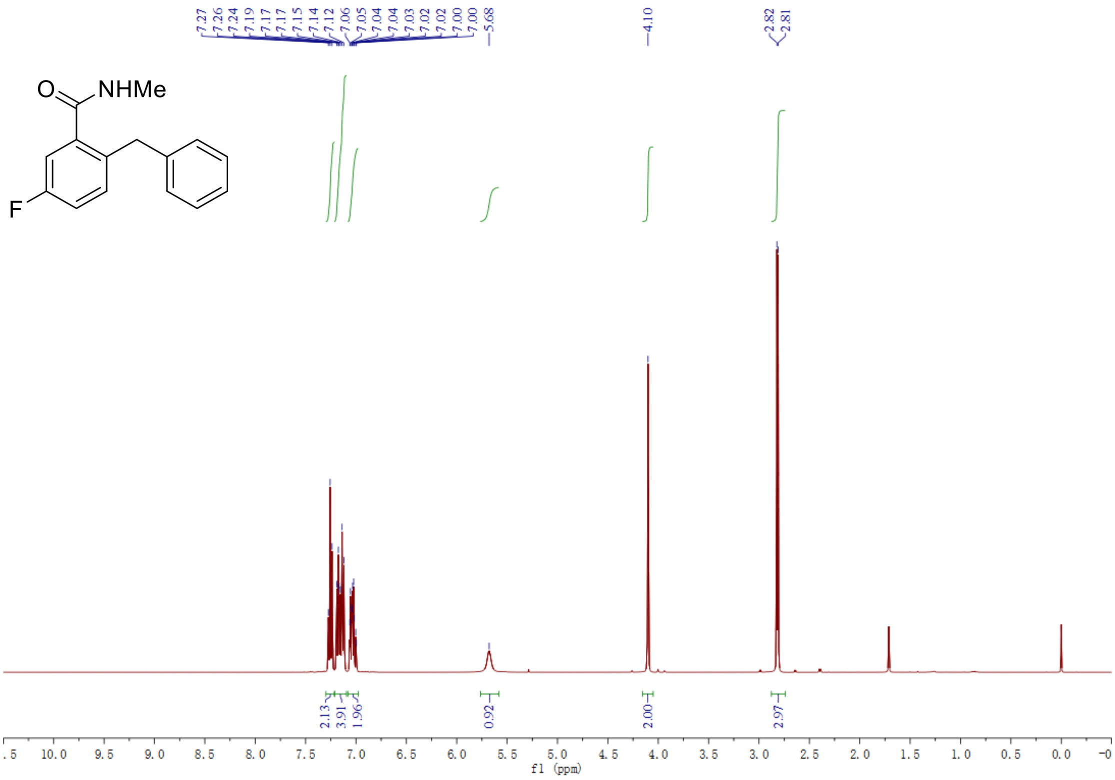

^{13}C NMR (100 MHz, $CDCl_3$)

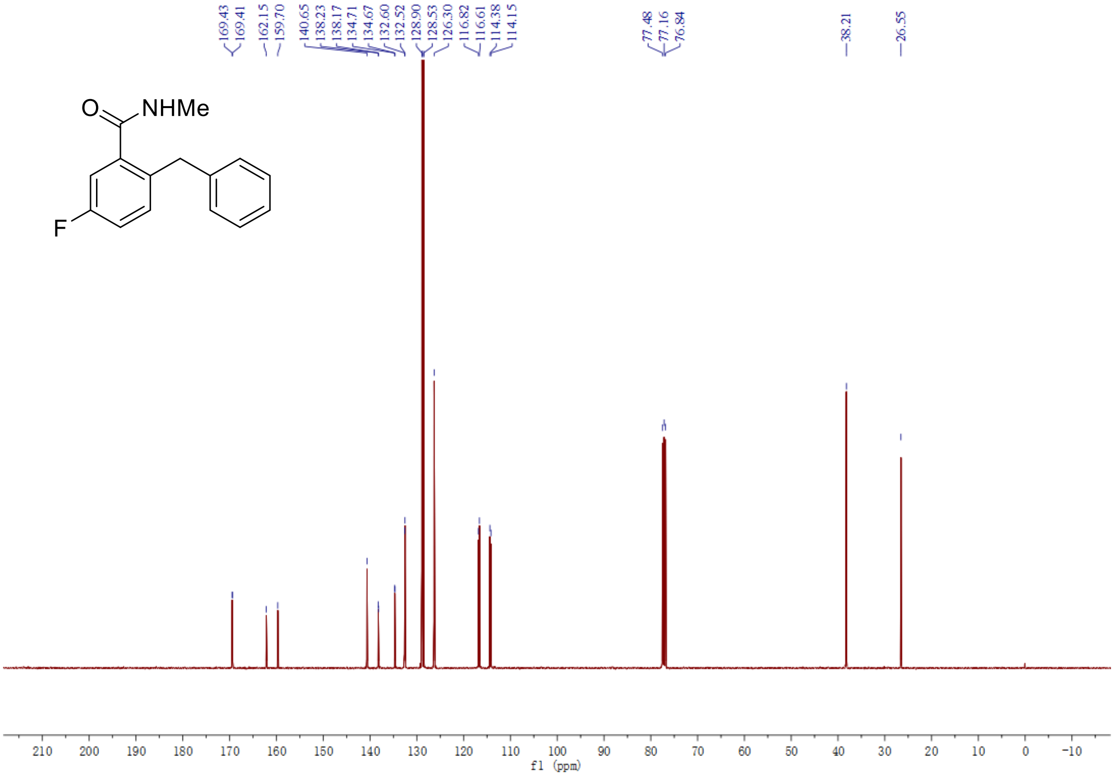

2-benzyl-5-chloro-N-methylbenzamide (3ma)

^{1}H NMR (400 MHz, $CDCl_3$)

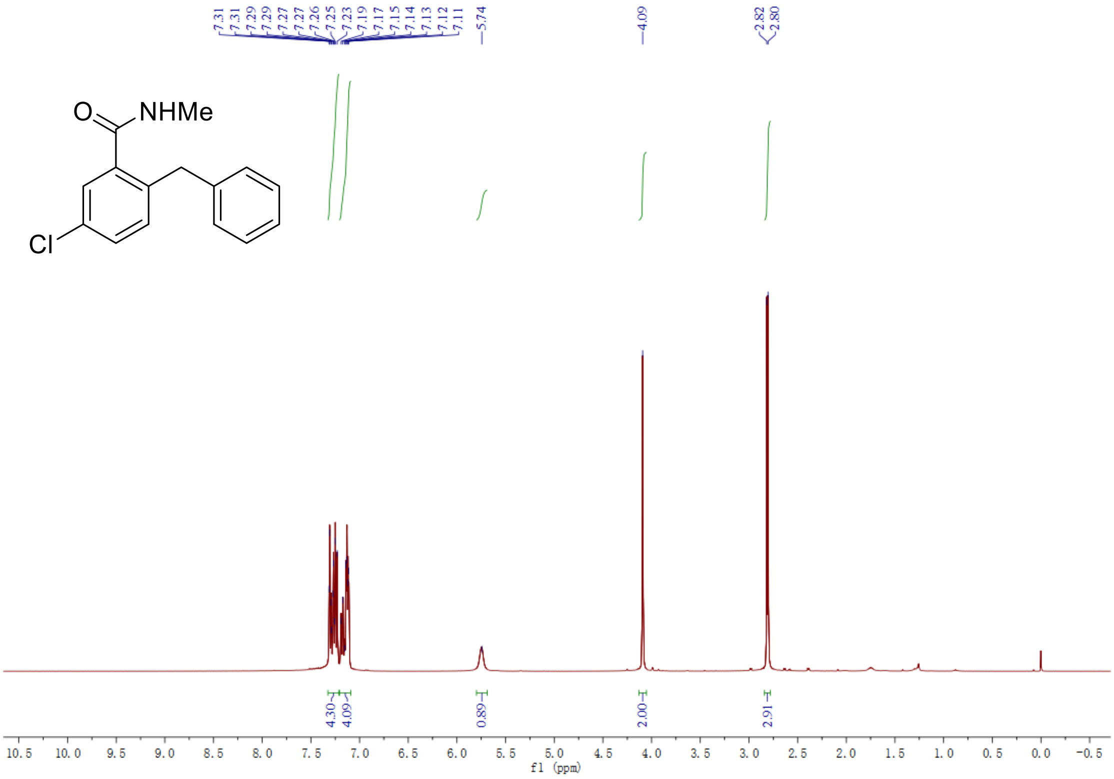

^{13}C NMR (100 MHz, $CDCl_3$)

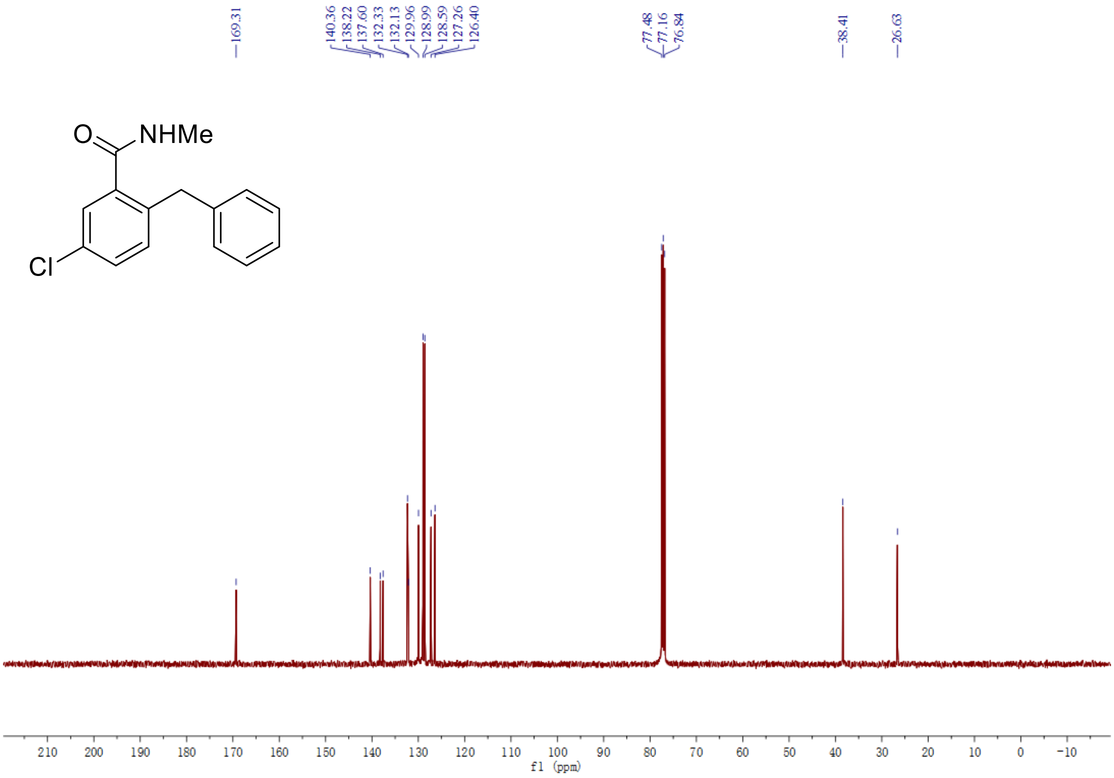

2-benzyl-3-methoxy-N-methylbenzamide (3na)

^{1}H NMR (400 MHz, $CDCl_3$)

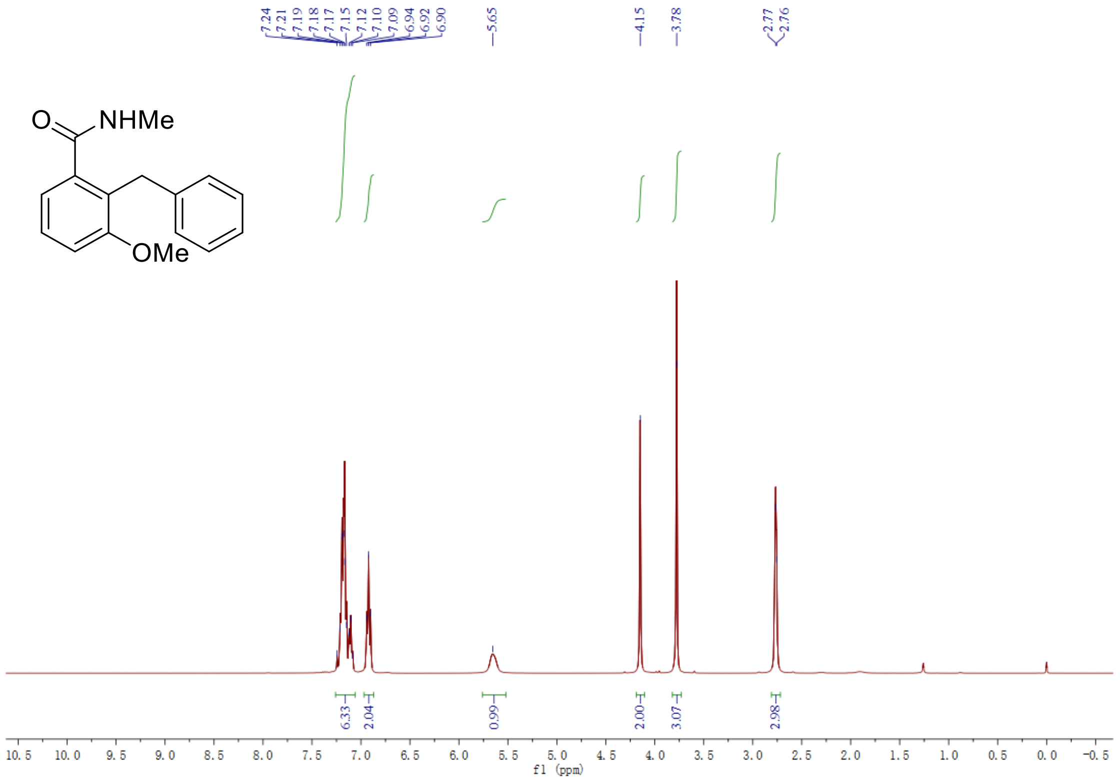

^{13}C NMR (100 MHz, $CDCl_3$)

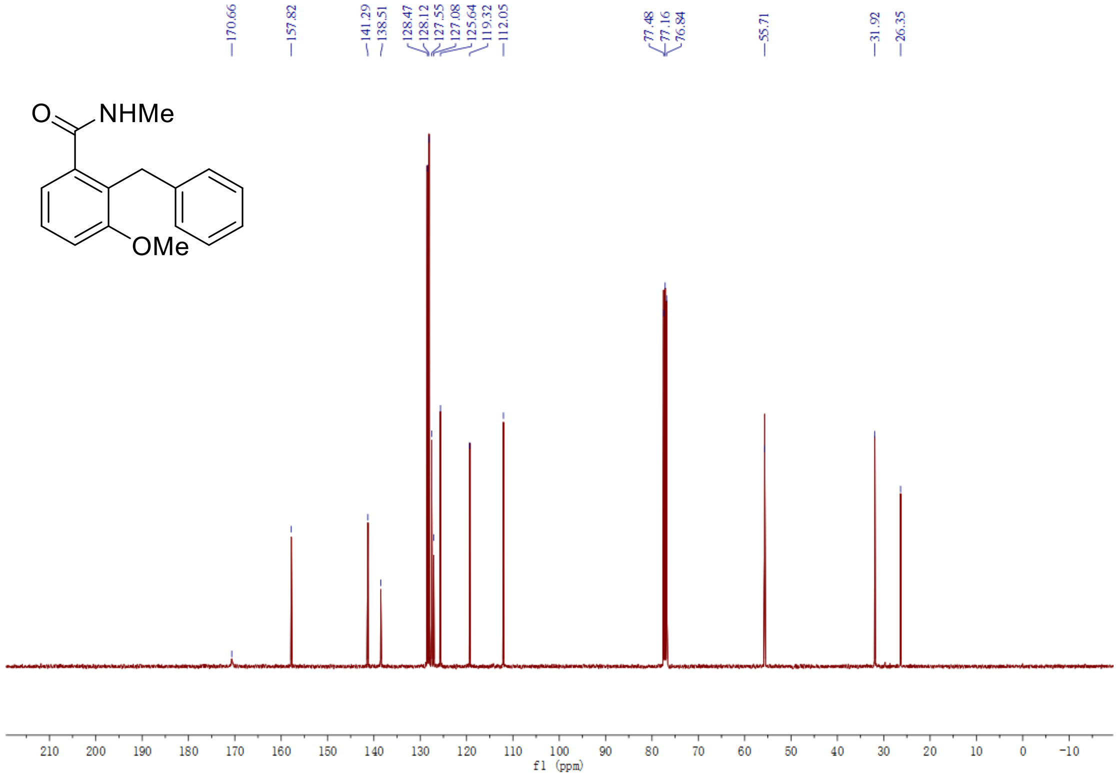

2-benzyl-N,3-dimethylbenzamide (3oa)

^{1}H NMR (400 MHz, $CDCl_3$)

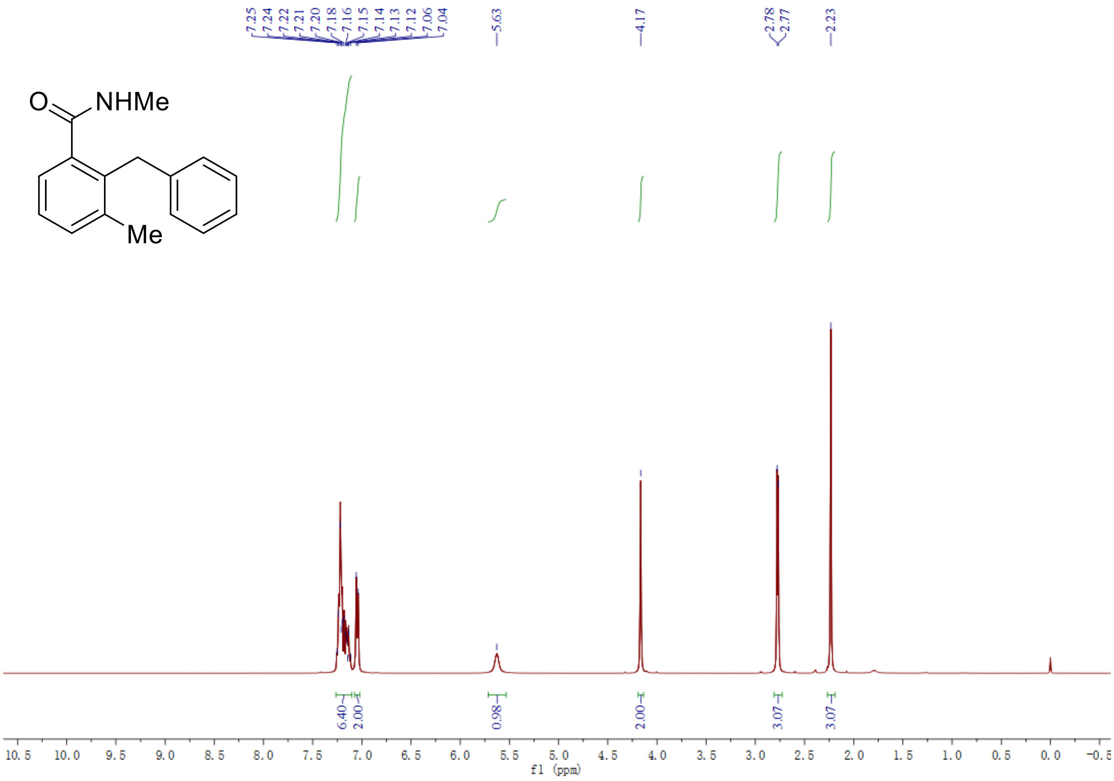

^{13}C NMR (100 MHz, $CDCl_3$)

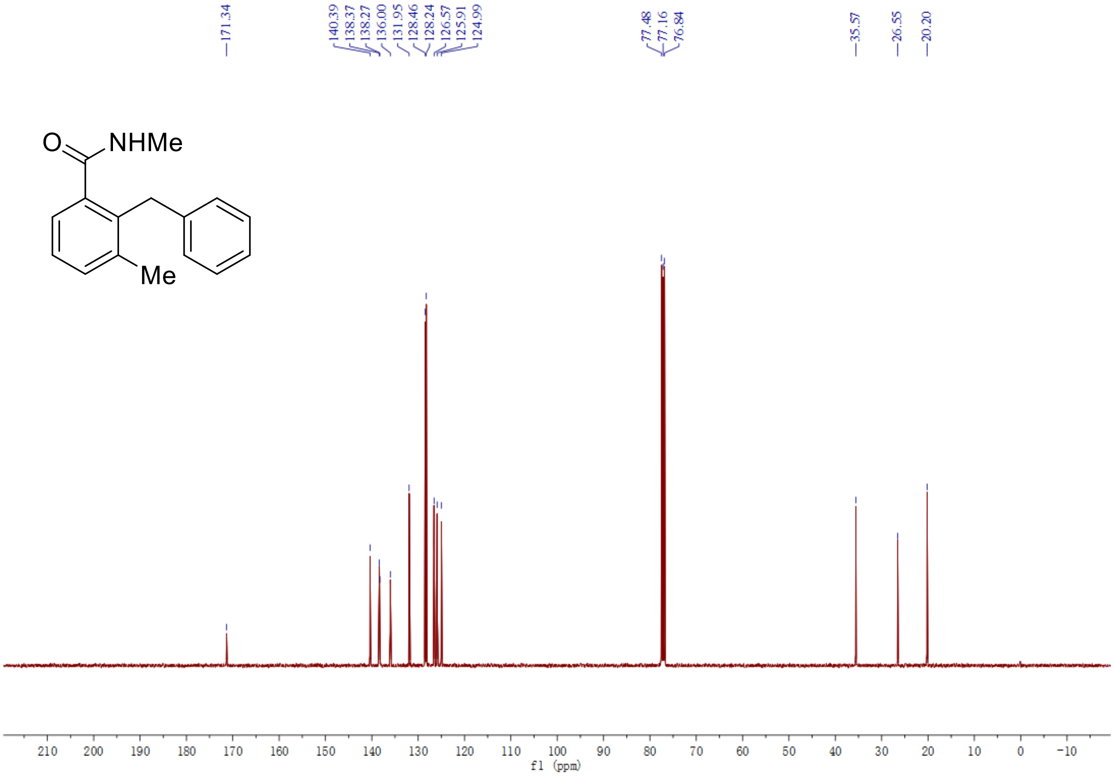

N-methyl-2-(2-methylbenzyl)benzamide (3ab)

^{1}H NMR (400 MHz, CDCl3)

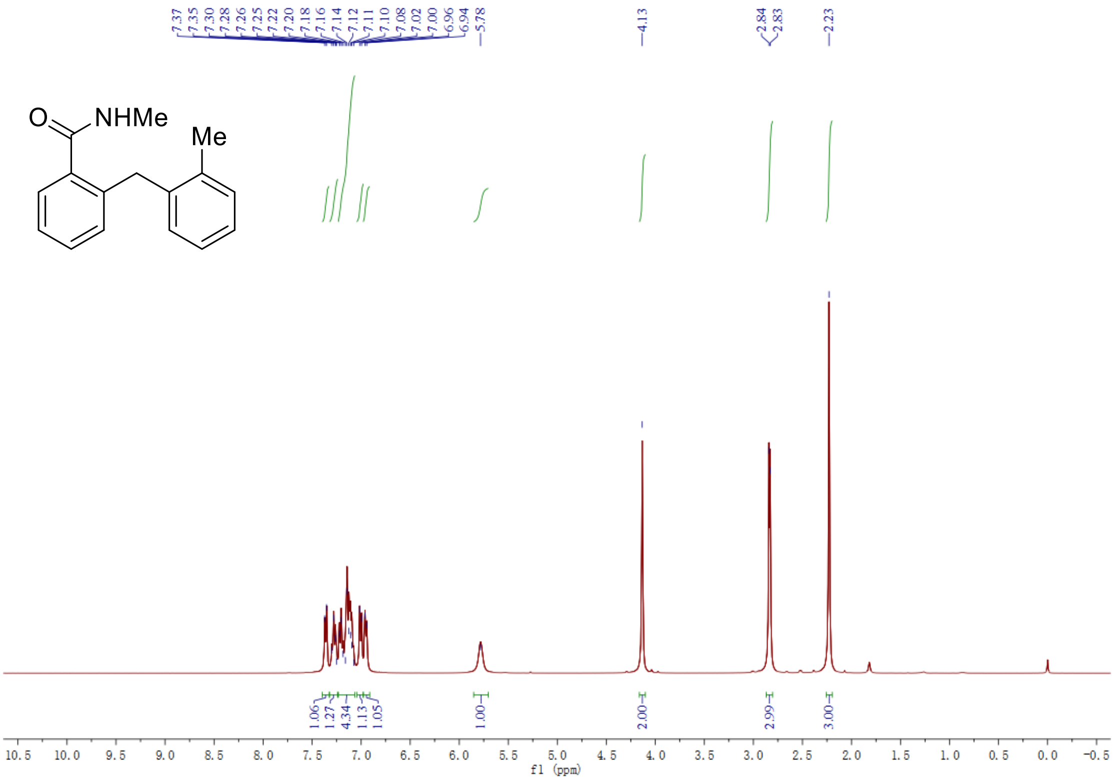

^{13}C NMR (100 MHz, CDCl3)

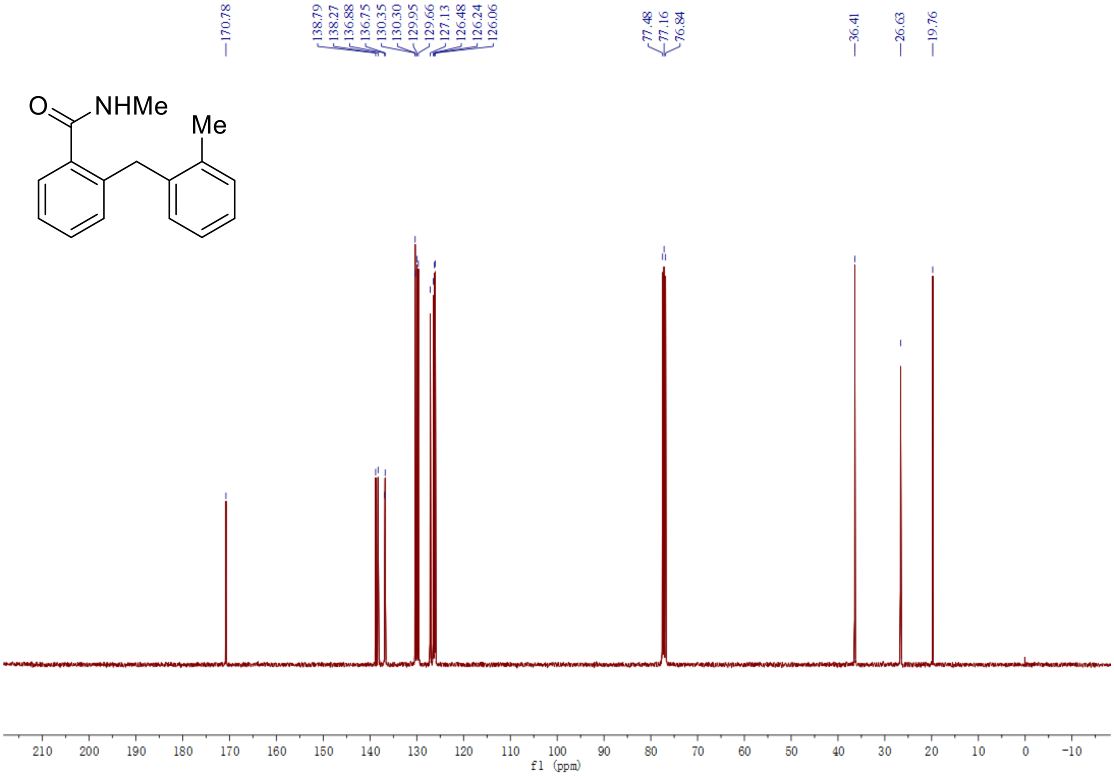

2-(2,6-dimethylbenzyl)-N-methylbenzamide (3ac)

^{1}H NMR (400 MHz, $CDCl_3$)

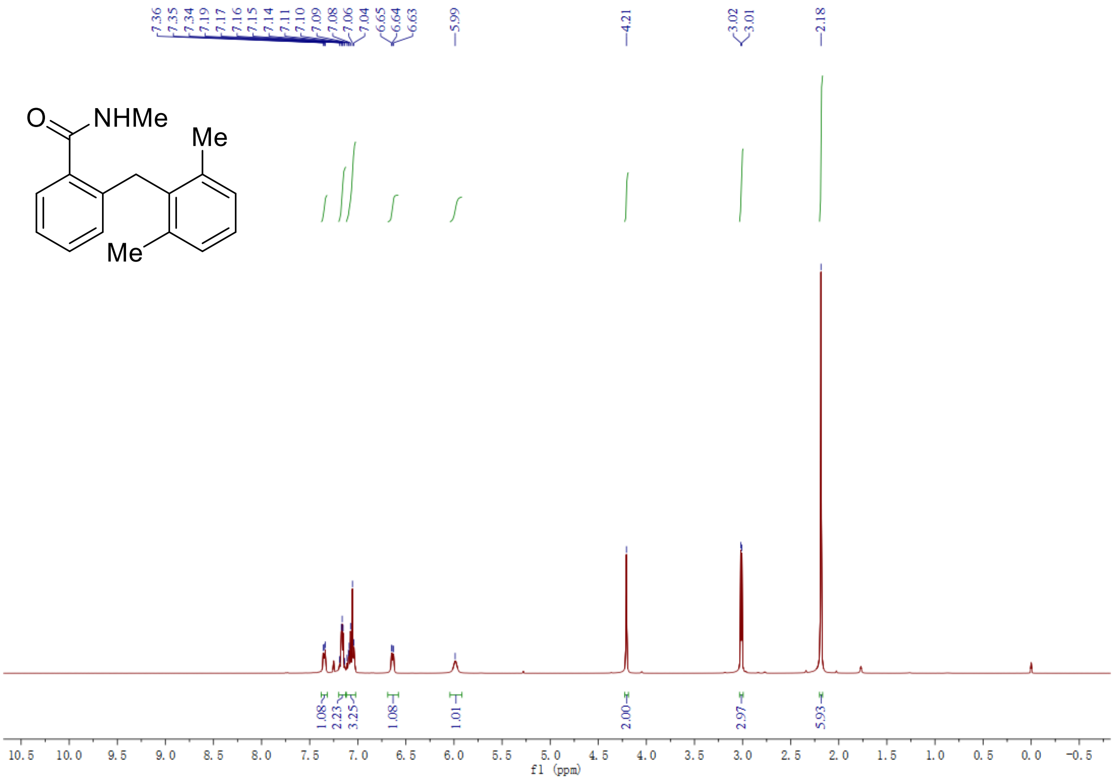

^{13}C NMR (100 MHz, $CDCl_3$)

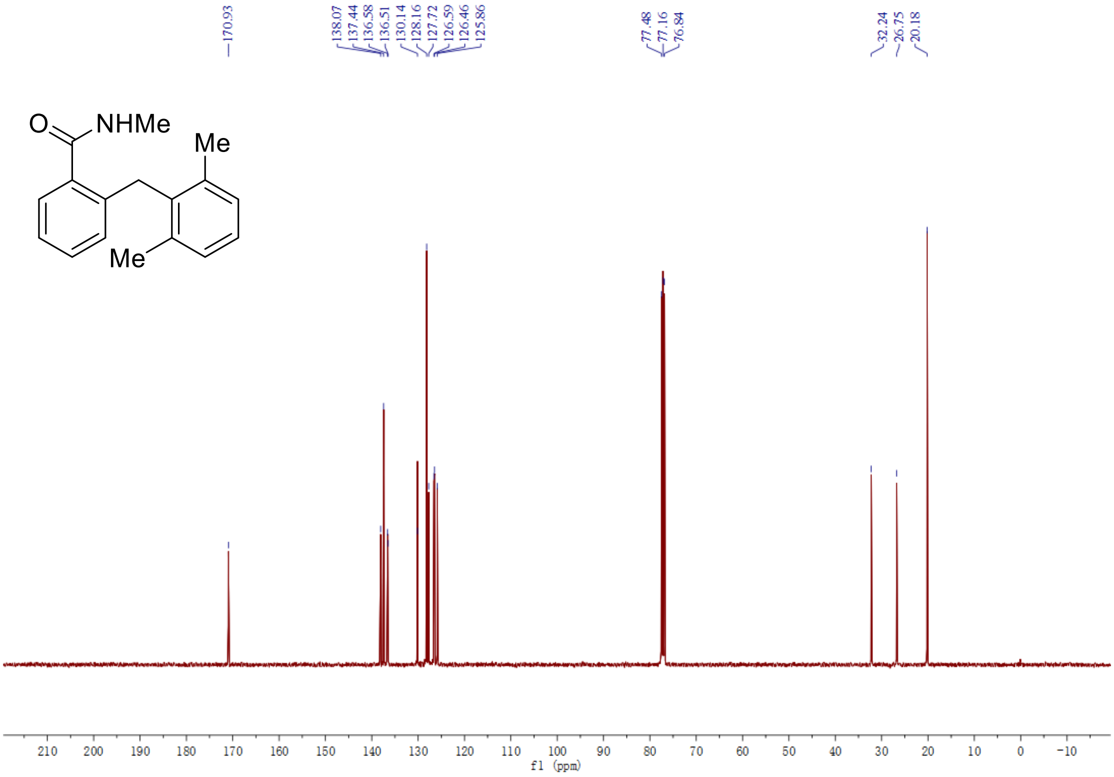

N-methyl-2-(4-methylbenzyl)benzamide(3ad)

^{1}H NMR (400 MHz, $CDCl_3$)

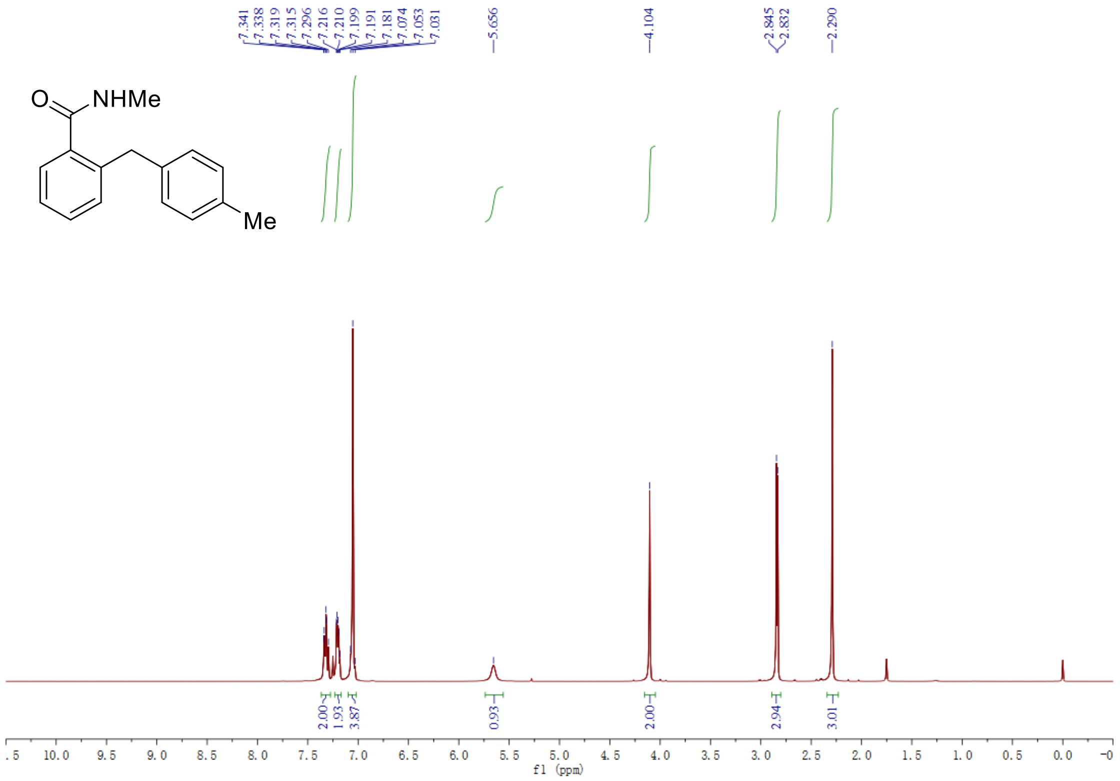

^{13}C NMR (100 MHz, $CDCl_3$)

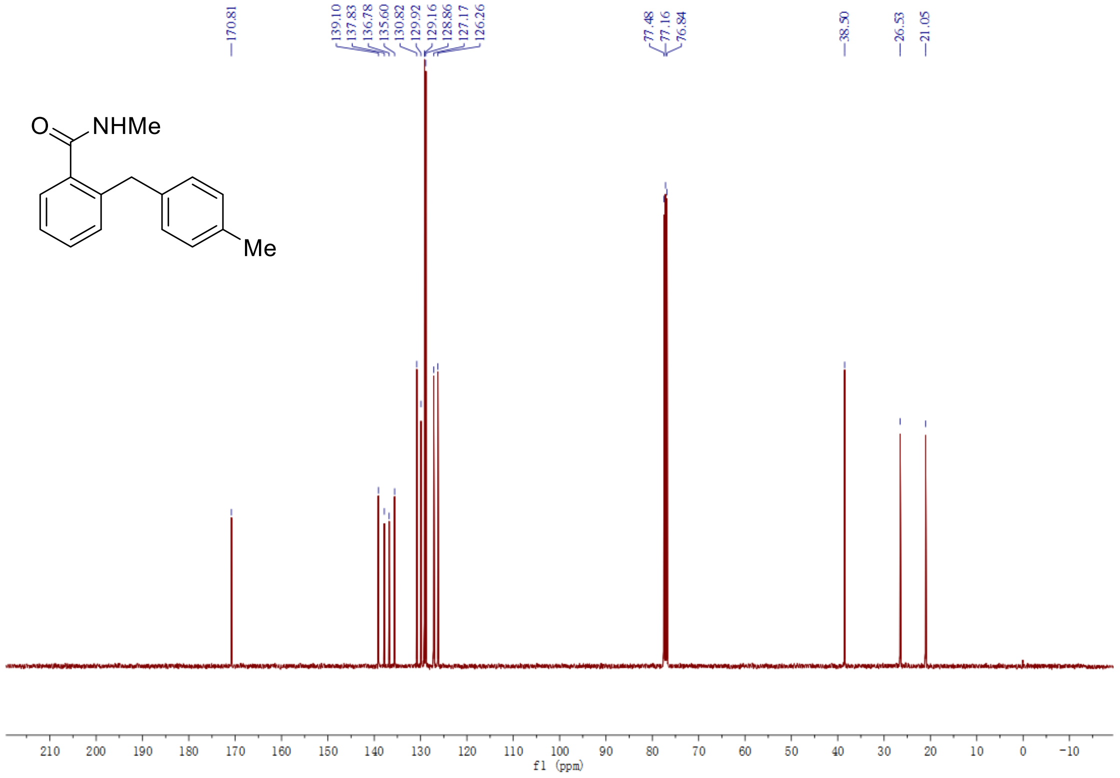

2-(4-fluorobenzyl)-N-methylbenzamide(3ae)

^{1}H NMR (400 MHz, $CDCl_3$)

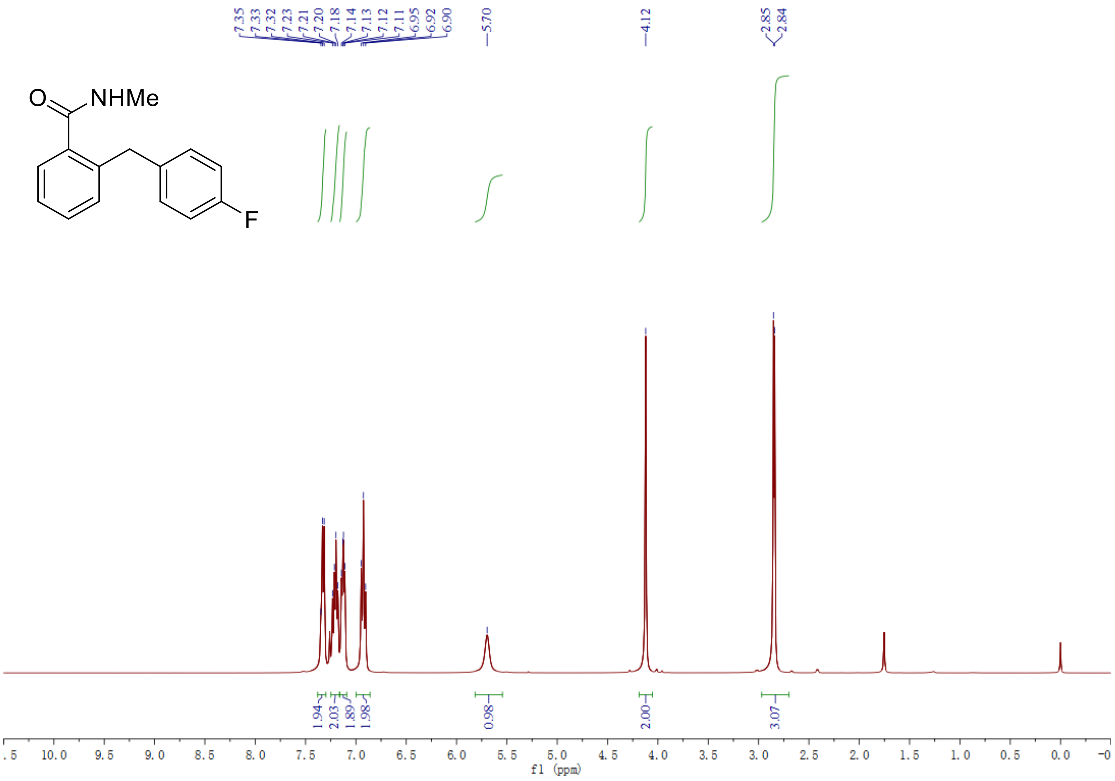

^{13}C NMR (100 MHz, $CDCl_3$)

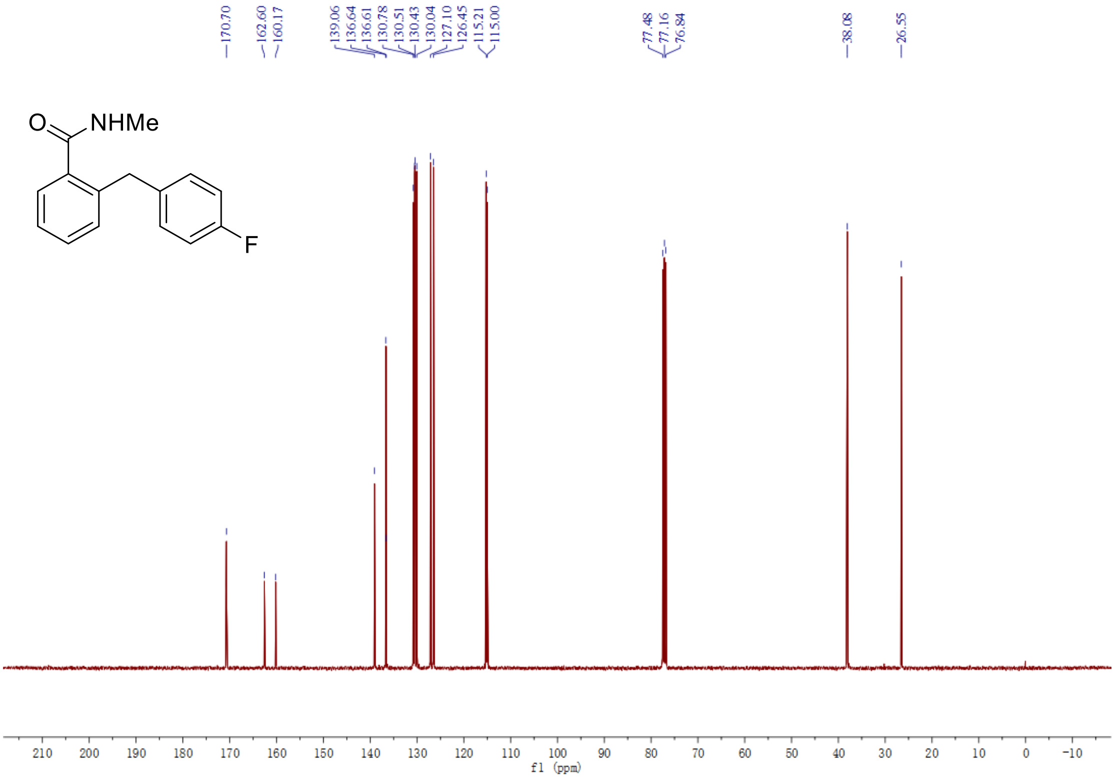

2-(4-chlorobenzyl)-N-methylbenzamide(3af)

^{1}H NMR (400 MHz, $CDCl_3$)

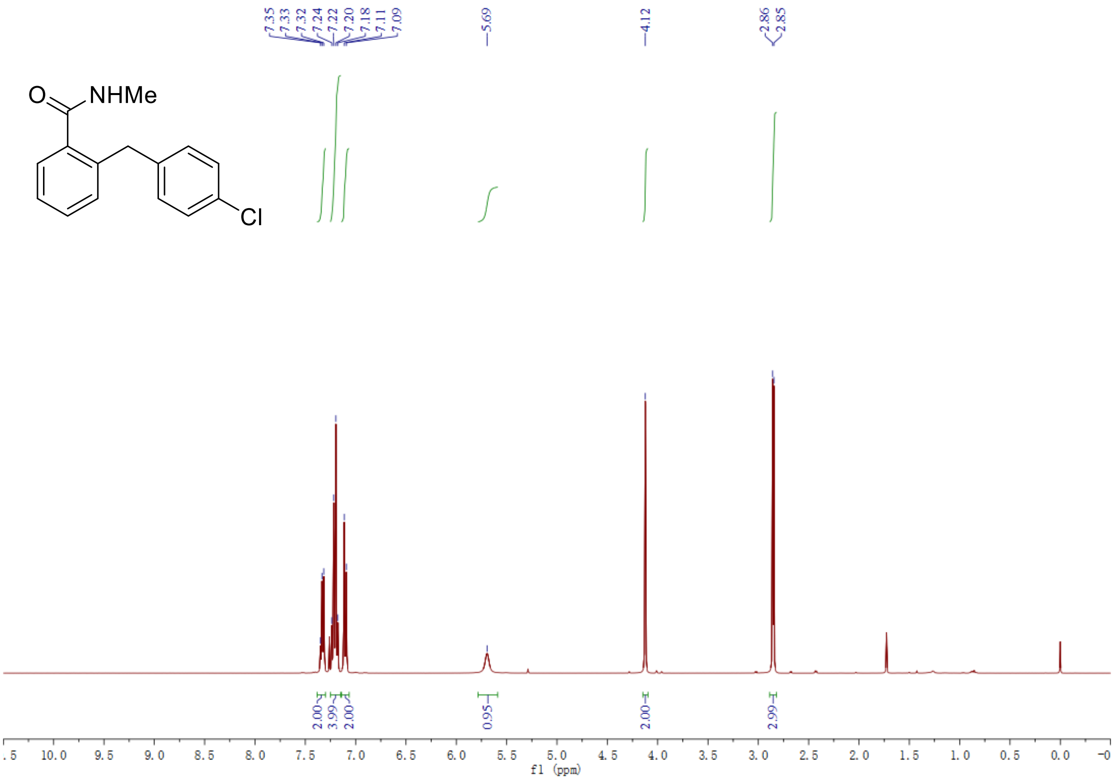

^{13}C NMR (100 MHz, $CDCl_3$)

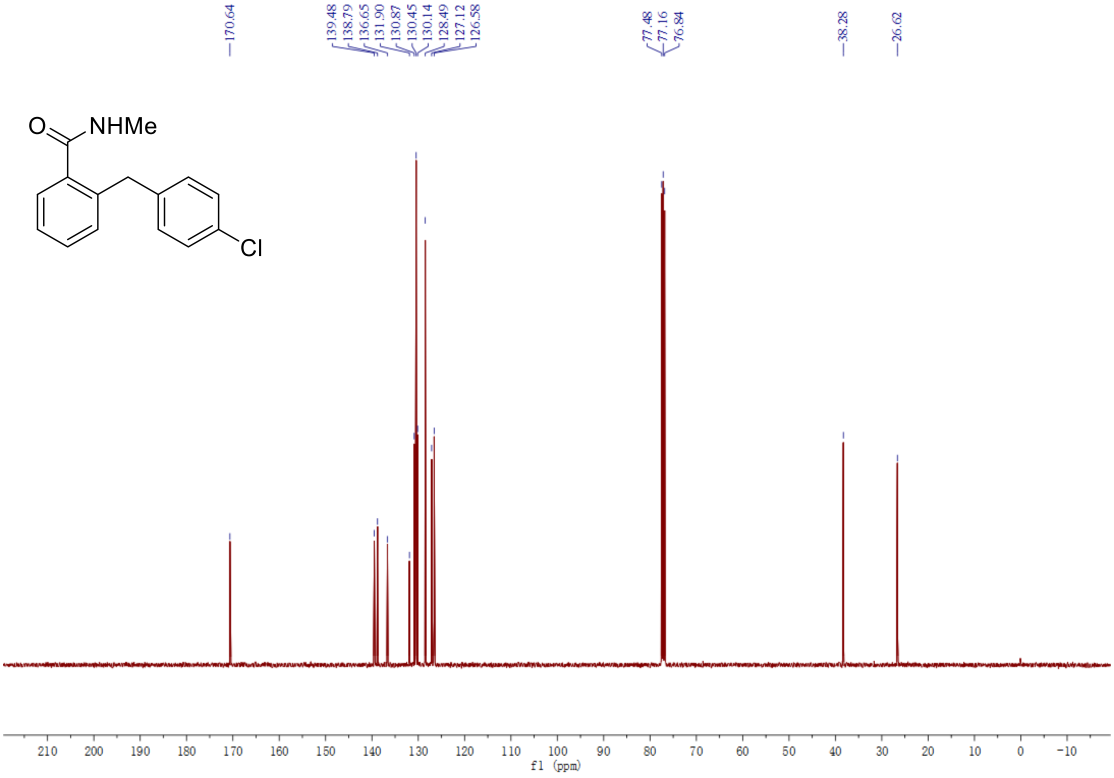

2-(4-bromobenzyl)-N-methylbenzamide(3ag)

^{1}H NMR (400 MHz, $CDCl_3$)

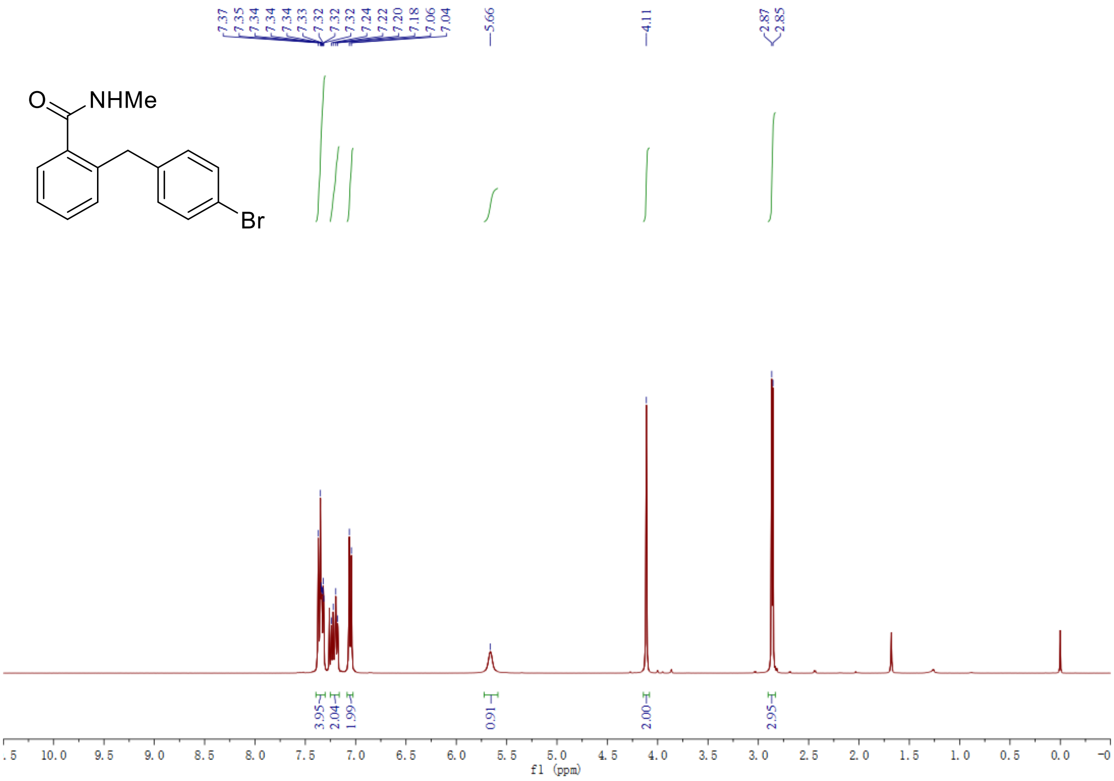

^{13}C NMR (100 MHz, $CDCl_3$)

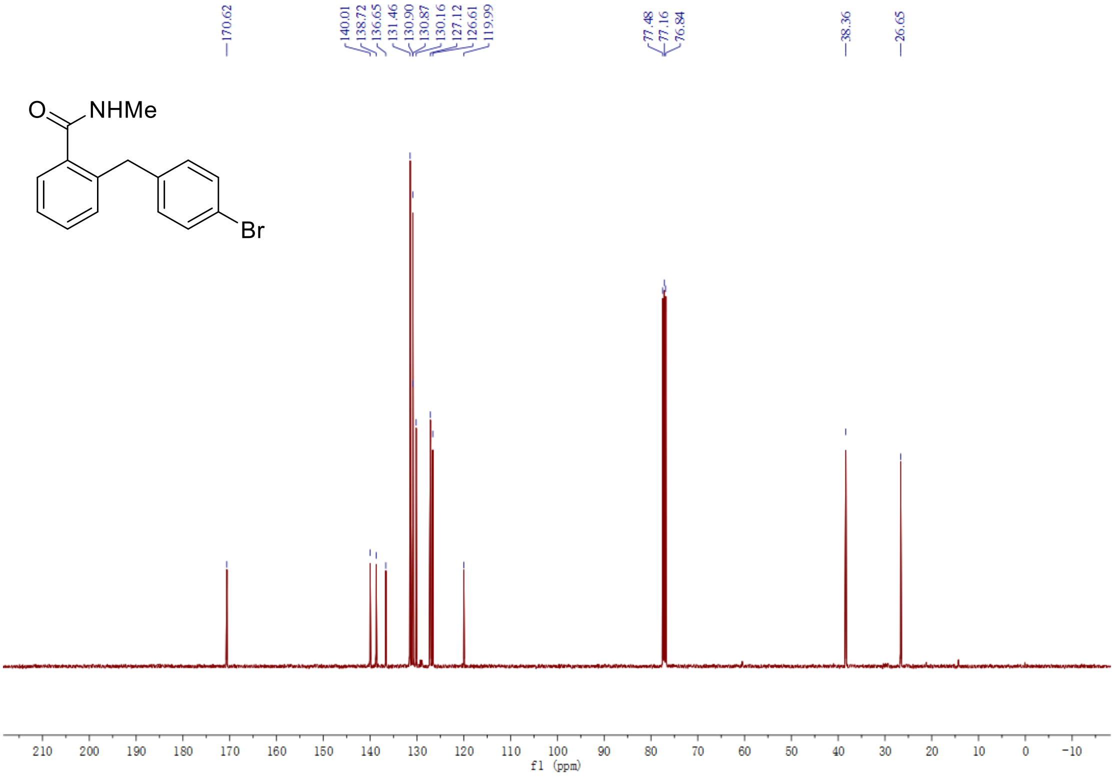

N-methyl-2-(4-(trifluoromethyl)benzyl)benzamide(3ah)

^{1}H NMR (400 MHz, $CDCl_3$)

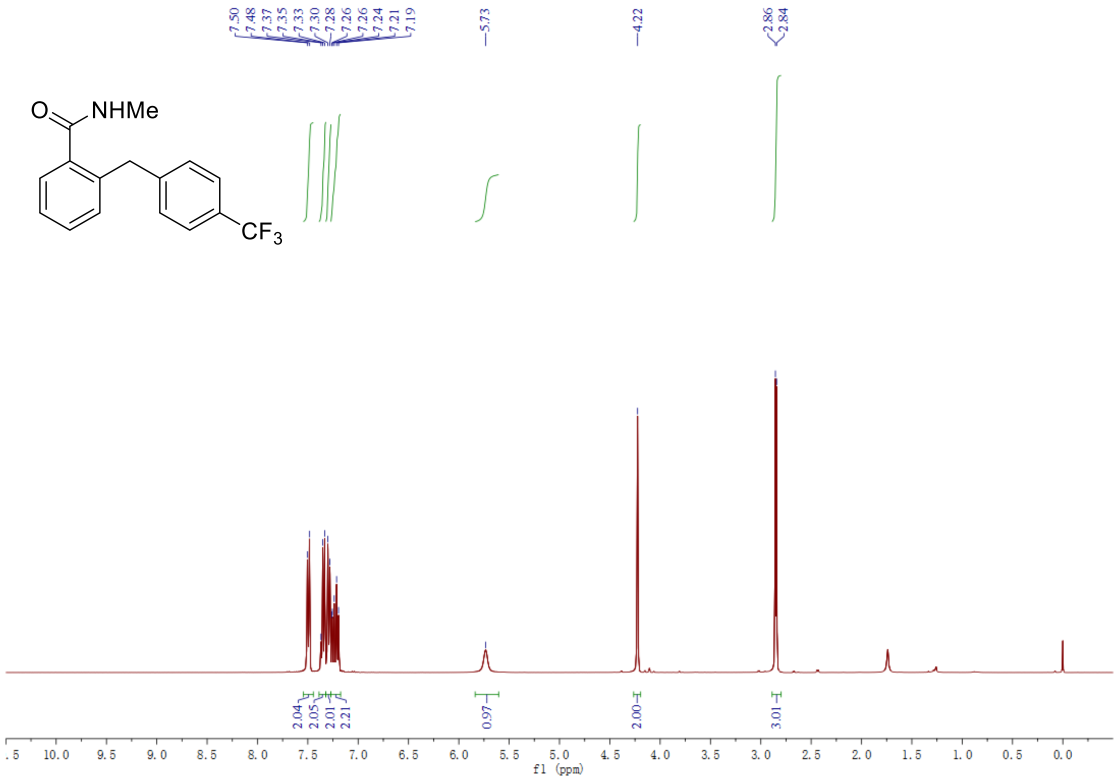

^{13}C NMR (100 MHz, $CDCl_3$)

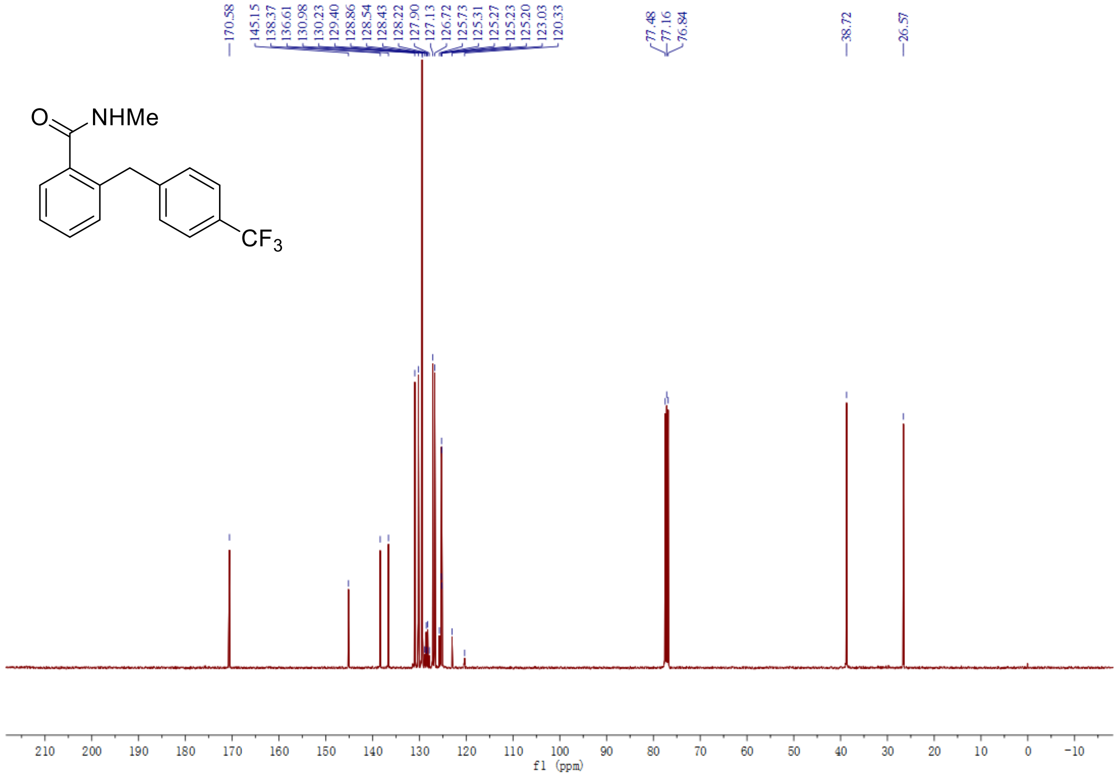

methyl 4-(2-(methylcarbamoyl)benzyl)benzoate (3ai)

^{1}H NMR (400 MHz, $CDCl_3$)

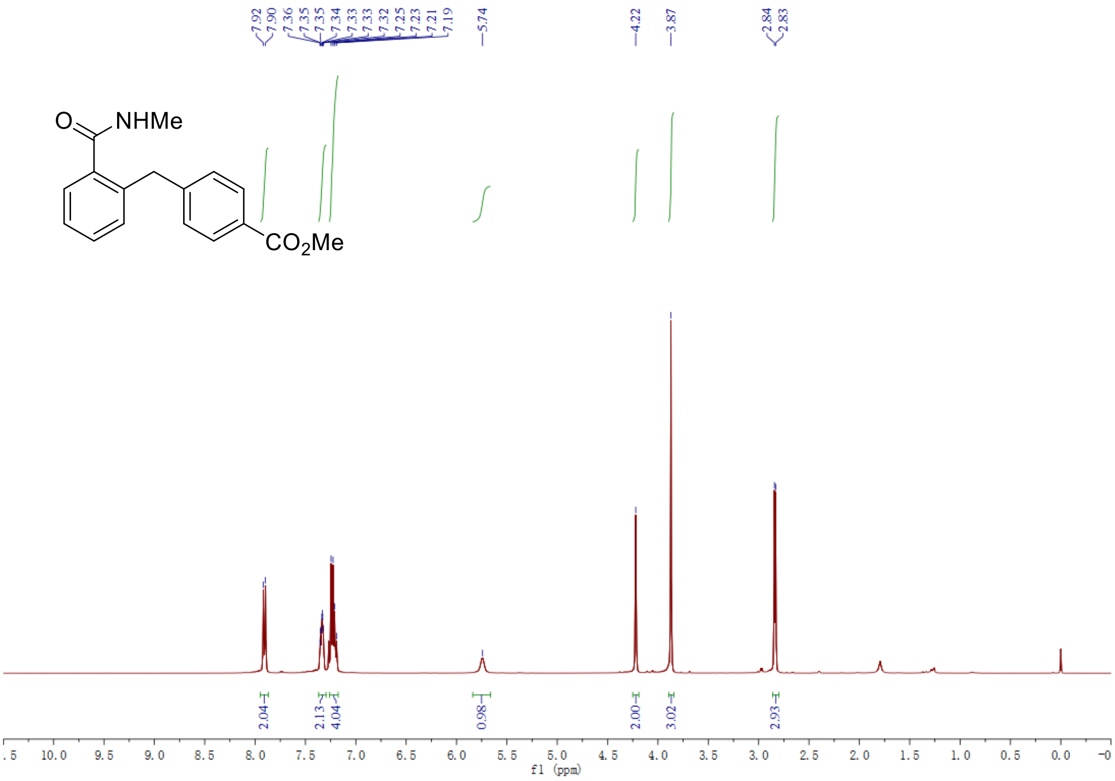

^{13}C NMR (100 MHz, $CDCl_3$)

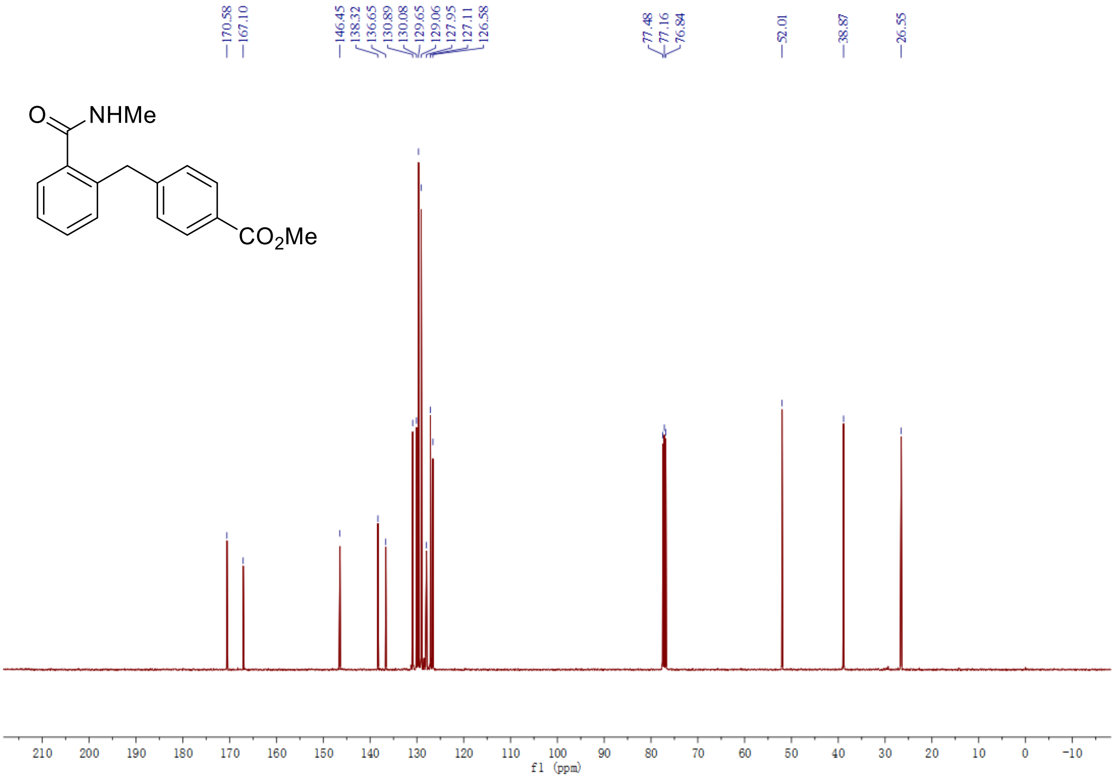

2-(4-cyanobenzyl)-N-methylbenzamide(3aj)

^{1}H NMR (400 MHz, $CDCl_3$)

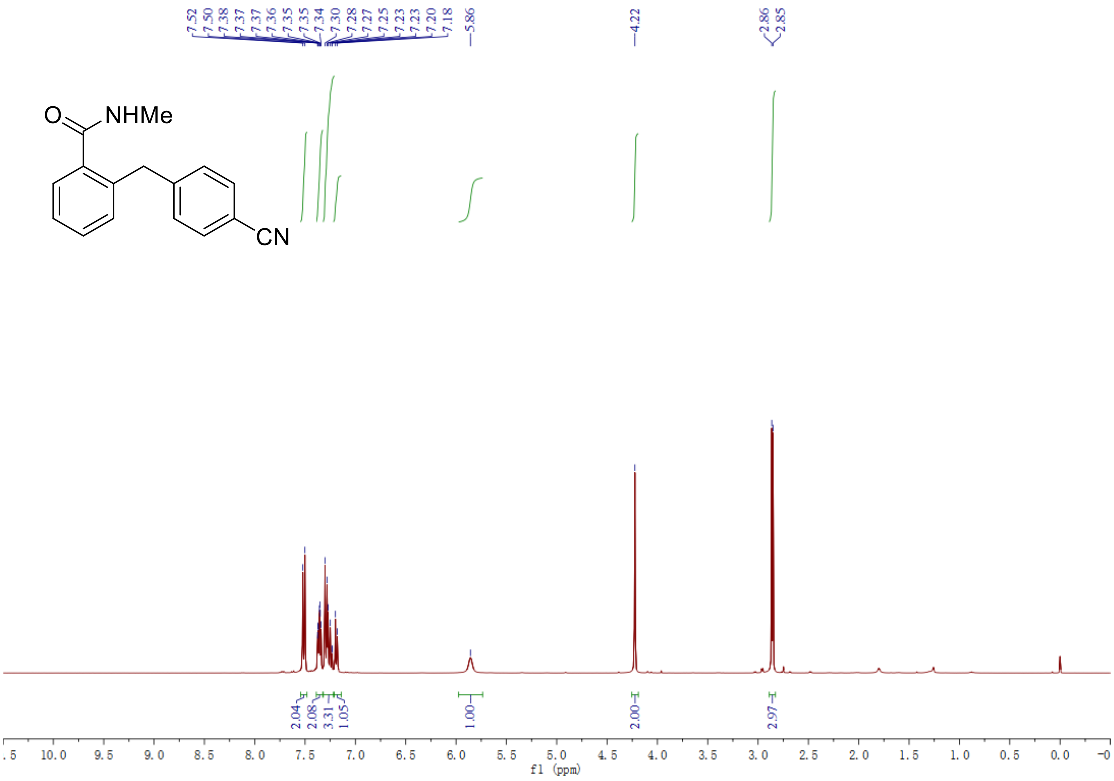

^{13}C NMR (100 MHz, $CDCl_3$)

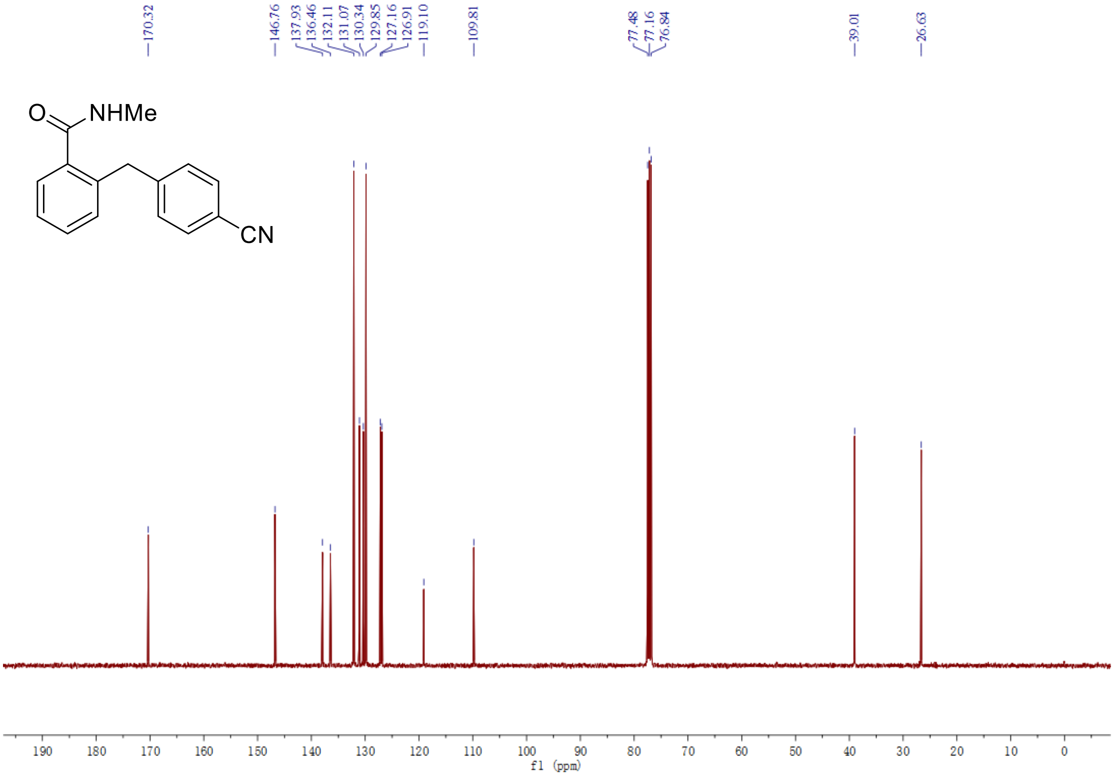

HRMS of new compounds

HRMS of 3ba

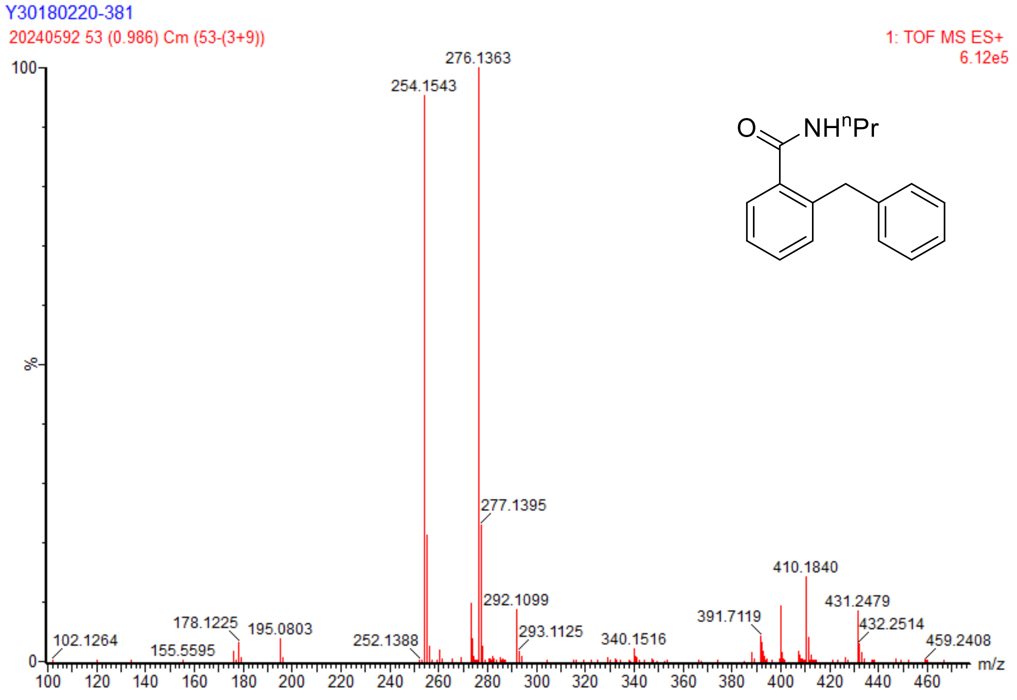

Elemental Composition Report

Multiple Mass Analysis: 2 mass(es) processed

Tolerance = 5.0 PPM / DBE: min = -1.5, max = 50.0

Element prediction: Off

Number of isotope peaks used for i-FIT = 3

Monoisotopic Mass, Even Electron Ions

7833 formula(e) evaluated with 6 results within limits (all results (up to 1000) for each mass)

Elements Used:

12C: 0-60 13C: 0-1 H: 0-60 N: 0-8 O: 0-8 Na: 0-1

Minimum: 15.00 -1.5

Maximum: 100.00 20.0 5.0 50.0

Mass	RA	Calc. Mass	mDa	PPM	DBE	i-FIT	Norm	Conf(%)	Formula
276.1363	100.00	276.1364	-0.1	-0.4	8.5	79.8	0.205	81.46	12C17 H19 N O Na

HRMS of 3ca

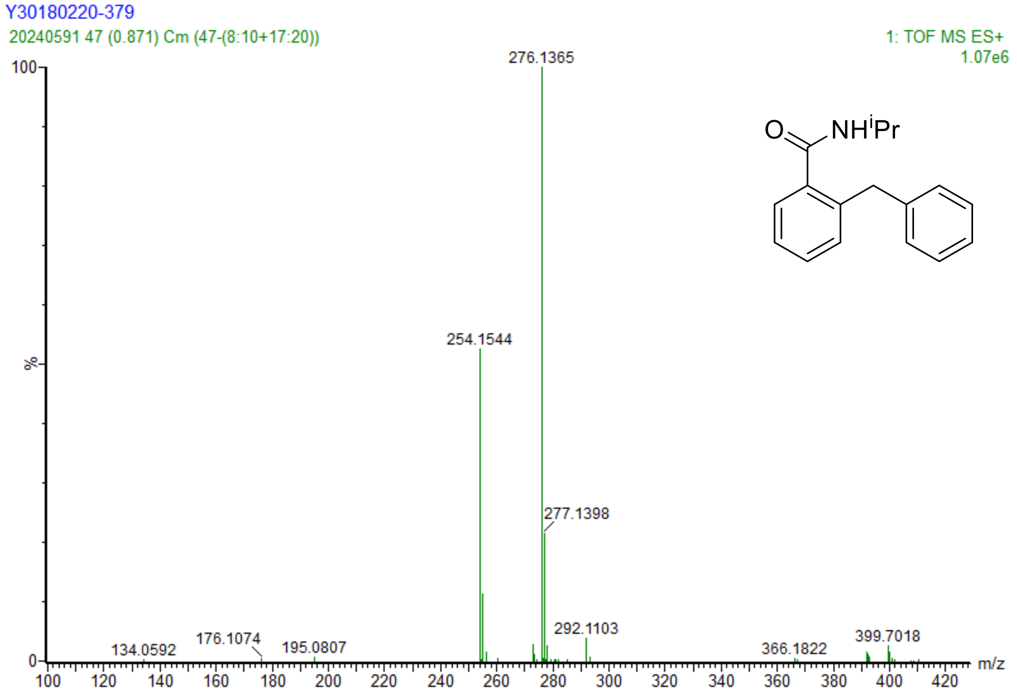

Elemental Composition Report

Multiple Mass Analysis: 2 mass(es) processed

Tolerance = 5.0 PPM / DBE: min = -1.5, max = 50.0

Element prediction: Off

Number of isotope peaks used for i-FIT = 3

Monoisotopic Mass, Even Electron Ions

7833 formula(e) evaluated with 6 results within limits (all results (up to 1000) for each mass)

Elements Used:

12C: 0-60 13C: 0-1 H: 0-60 N: 0-8 O: 0-8 Na: 0-1

Minimum: 15.00 -1.5

Maximum: 100.00 20.0 5.0 50.0

Mass	RA	Calc. Mass	mDa	PPM	DBE	i-FIT	Norm	Conf(%)	Formula
276.1365	100.00	276.1365	0.0	0.0	0.5	139.5	1.935	14.44	12C7 13C H20 N4 O5 Na

HRMS of 3ea

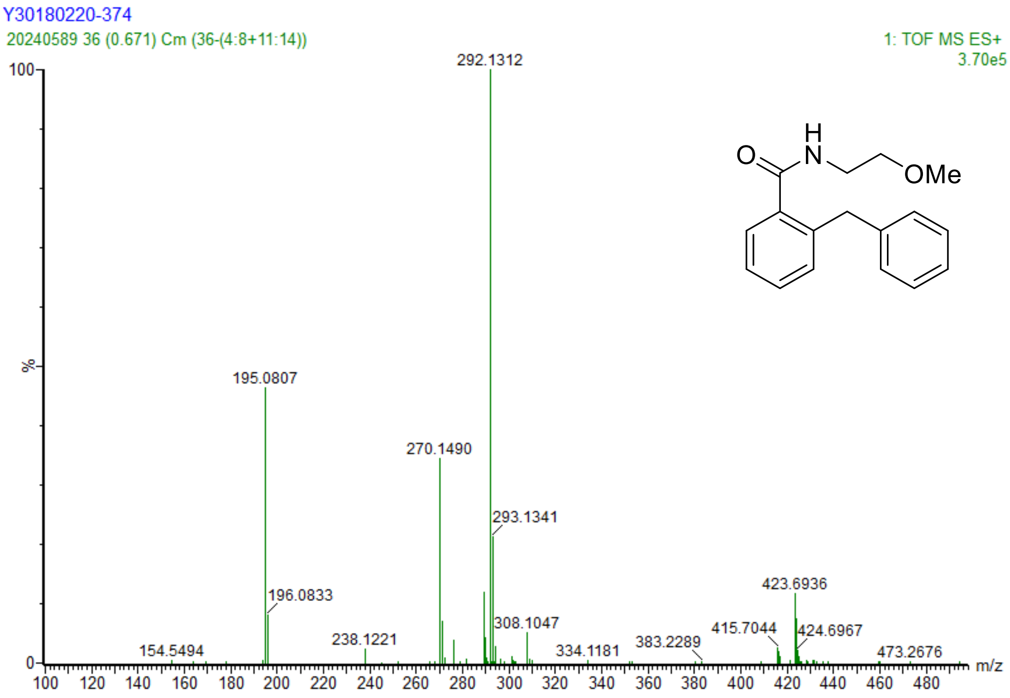

Elemental Composition Report

Multiple Mass Analysis: 2 mass(es) processed

Tolerance = 5.0 PPM / DBE: min = -1.5, max = 50.0

Element prediction: Off

Number of isotope peaks used for i-FIT = 3

Monoisotopic Mass, Even Electron Ions

8697 formula(e) evaluated with 10 results within limits (all results (up to 1000) for each mass)

Elements Used:

12C: 0-60 13C: 0-1 H: 0-60 N: 0-8 O: 0-8 Na: 0-1

Minimum:		15.00				-1.5			
Maximum:		100.00		20.0	5.0	50.0			
Mass	RA	Calc. Mass	mDa	PPM	DBE	i-FIT	Norm	Conf(%)	Formula
292.1312	100.00	292.1313	-0.1	-0.3	8.5	58.9	0.149	86.13	12C17 H19 N O2 Na

HRMS of 3fa

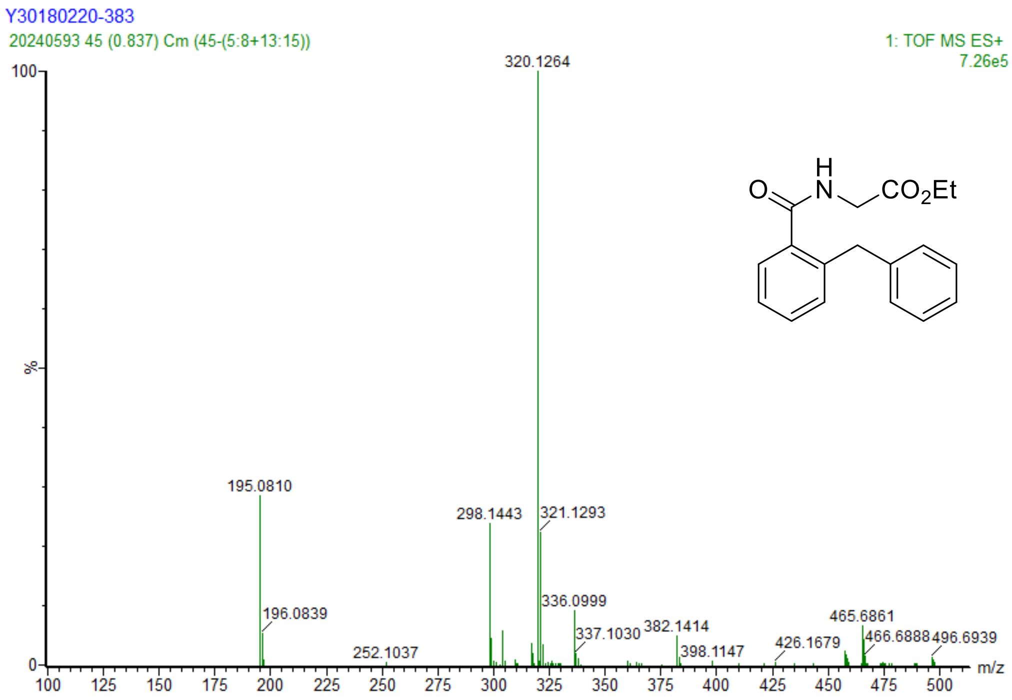

Elemental Composition Report

Multiple Mass Analysis: 2 mass(es) processed

Tolerance = 5.0 PPM / DBE: min = -1.5, max = 50.0

Element prediction: Off

Number of isotope peaks used for i-FIT = 3

Monoisotopic Mass, Even Electron Ions

10209 formula(e) evaluated with 11 results within limits (all results (up to 1000) for each mass)

Elements Used:

12C: 0-60 13C: 0-1 H: 0-60 N: 0-8 O: 0-8 Na: 0-1

Minimum: 15.00 -1.5

Maximum: 100.00 20.0 5.0 50.0

Mass	RA	Calc. Mass	mDa	PPM	DBE	i-FIT	Norm	Conf(%)	Formula
320.1264	100.00	320.1263	0.1	0.3	1.5	82.5	2.012	13.37	12C8 13C H20 N4 O7 Na

HRMS of 3ia

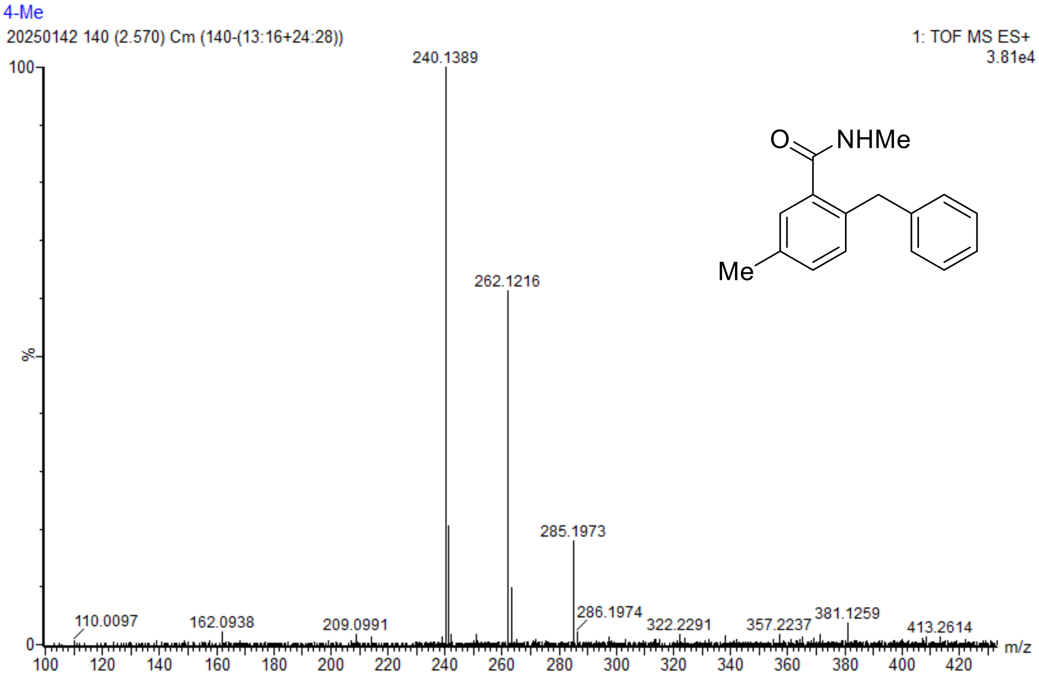

Elemental Composition Report

Multiple Mass Analysis: 2 mass(es) processed

Tolerance = 5.0 PPM / DBE: min = -1.5, max = 50.0

Element prediction: Off

Number of isotope peaks used for i-FIT = 3

Monoisotopic Mass, Even Electron Ions

4545 formula(e) evaluated with 5 results within limits (all results (up to 1000) for each mass)

Elements Used:

12C: 0-60 13C: 0-1 H: 0-60 N: 0-6 O: 0-6 Na: 0-1

Mass	RA	Calc. Mass	mDa	PPM	DBE	i-FIT	Norm	Conf(%)	Formula
Minimum:					-1.5				
Maximum:				20.0	5.0	50.0			
240.1389	100.00	240.1389	0.0	0.0	0.5	95.3	1.301	27.24	12C6 13C H19 N4 O5

HRMS of 3ja

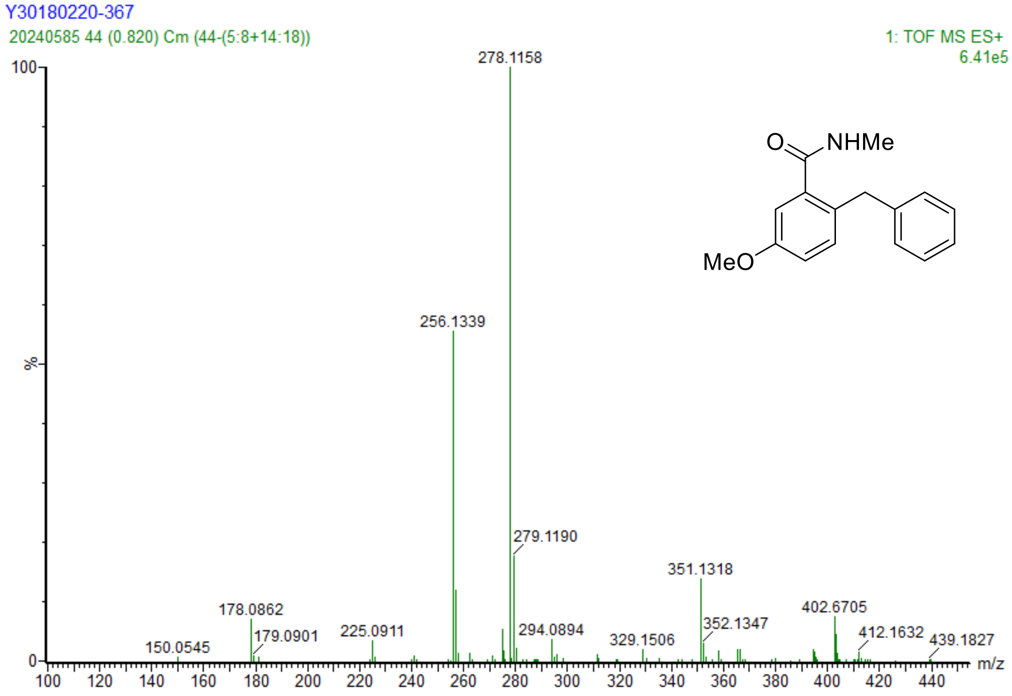

Elemental Composition Report

Multiple Mass Analysis: 2 mass(es) processed

Tolerance = 5.0 PPM / DBE: min = -1.5, max = 50.0

Element prediction: Off

Number of isotope peaks used for i-FIT = 3

Monoisotopic Mass, Even Electron Ions

7935 formula(e) evaluated with 8 results within limits (all results (up to 1000) for each mass)

Elements Used:

12C: 0-60 13C: 0-1 H: 0-60 N: 0-8 O: 0-8 Na: 0-1

Minimum:		15.00				-1.5	
Maximum:		100.00		20.0	5.0	50.0	

Mass	RA	Calc. Mass	mDa	PPM	DBE	i-FIT	Norm	Conf(%)	Formula
278.1158	100.00	278.1158	0.0	0.0	0.5	66.3	2.065	12.68	12C6 13C H18 N4 O6 Na

HRMS of 3ka

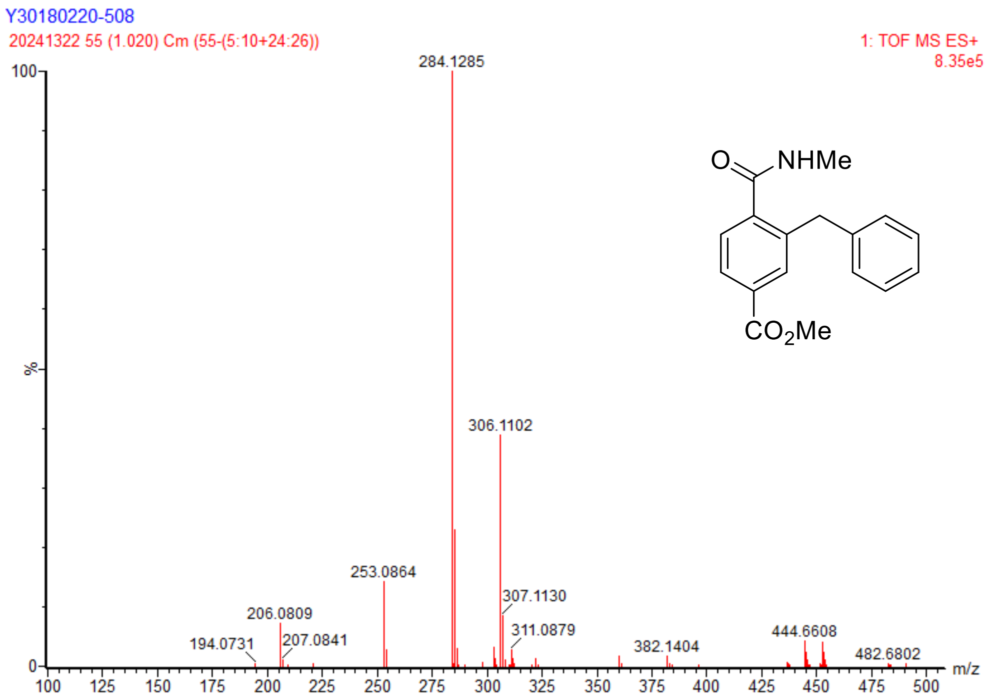

Elemental Composition Report

Multiple Mass Analysis: 2 mass(es) processed

Tolerance = 5.0 PPM / DBE: min = -1.5, max = 50.0

Element prediction: Off

Number of isotope peaks used for i-FIT = 3

Monoisotopic Mass, Even Electron Ions

8265 formula(e) evaluated with 9 results within limits (all results (up to 1000) for each mass)

Elements Used:

12C: 0-60 13C: 0-1 H: 0-60 N: 0-8 O: 0-8 Na: 0-1

Minimum:	15.00					-1.5	
Maximum:	100.00			20.0	5.0	50.0	
Mass	RA	Calc. Mass	mDa	PPM	DBE	i-FIT	Norm
Conf(%)	Formula						
284.1285	100.00	284.1287	-0.2	-0.7	9.5	162.6	0.171
84.28	12C17 H18 N O3						

HRMS of 3la

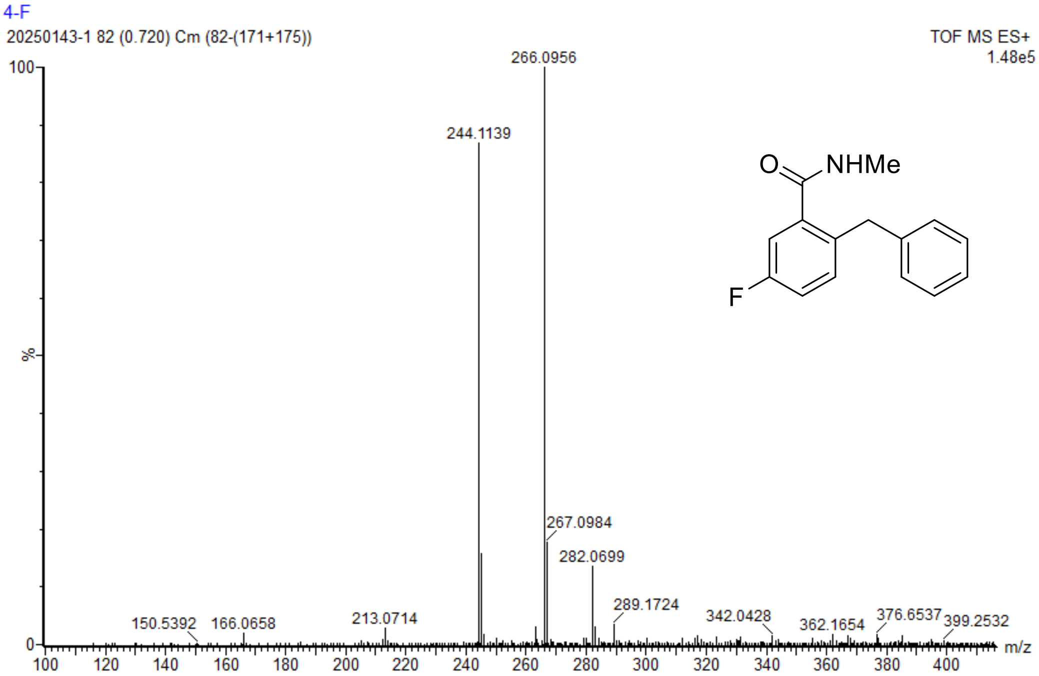

Elemental Composition Report

Multiple Mass Analysis: 2 mass(es) processed

Tolerance = 8.0 PPM / DBE: min = -1.5, max = 50.0

Element prediction: Off

Number of isotope peaks used for i-FIT = 3

Monoisotopic Mass, Even Electron Ions

4768 formula(e) evaluated with 8 results within limits (all results (up to 1000) for each mass)

Elements Used:

12C: 0-60 13C: 0-1 H: 0-60 N: 0-6 O: 0-6 F: 1-1 Na: 0-1

Minimum:		5.00				-1.5		
Maximum:		100.00		20.0	8.0	50.0		
Mass	RA	Calc. Mass	mDa	PPM	DBE	i-FIT	Norm	Conf(%) Formula
266.0956	100.00	266.0957	-0.1	-0.4	8.5	161.1	0.448	63.87 12C15 H14 N O F Na

HRMS of 3ma

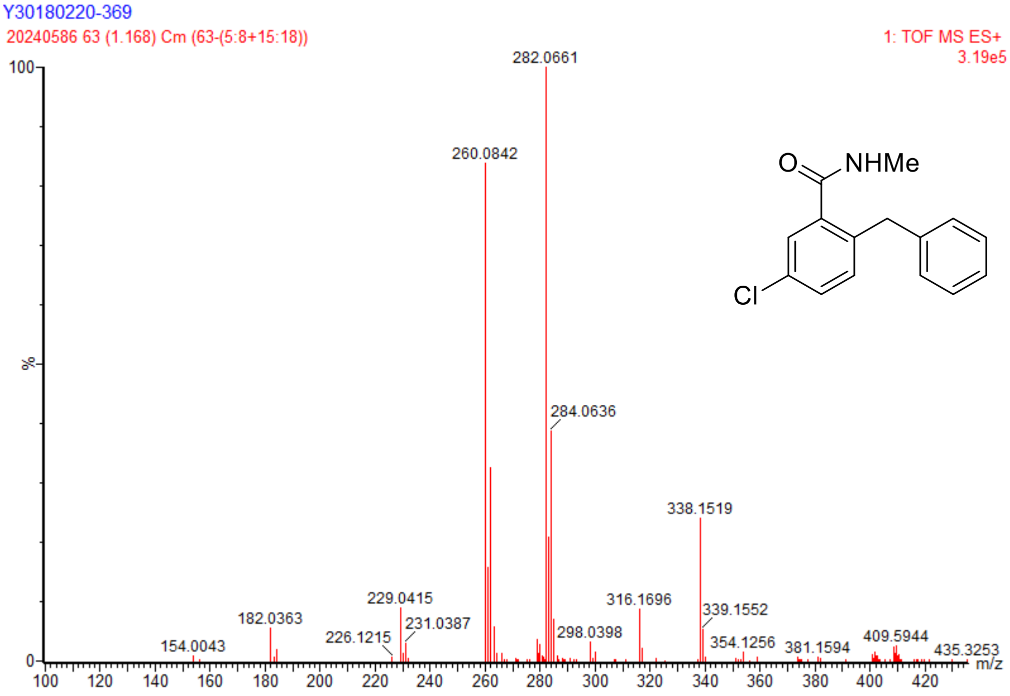

Elemental Composition Report
Multiple Mass Analysis: 4 mass(es) processed
Tolerance = 5.0 PPM / DBE: min = -1.5, max = 50.0
Element prediction: Off
Number of isotope peaks used for i-FIT = 3
Monoisotopic Mass, Even Electron Ions
9888 formula(e) evaluated with 12 results within limits (all results (up to 1000) for each mass)
Elements Used:
12C: 0-60 13C: 0-1 H: 0-60 N: 0-2 O: 0-2 Na: 0-1 35Cl: 0-1
37Cl: 0-1

Mass	RA	Calc. Mass	mDa	PPM	DBE	i-FIT	Norm	Conf(%)	Formula
Minimum:	2.00				-1.5				
Maximum:	100.00			20.0	5.0	50.0			
282.0661	100.00	282.0662	-0.1	-0.4	8.5	54.0	2.205	11.03	12C15 H14 N O Na 35Cl

HRMS of 3na

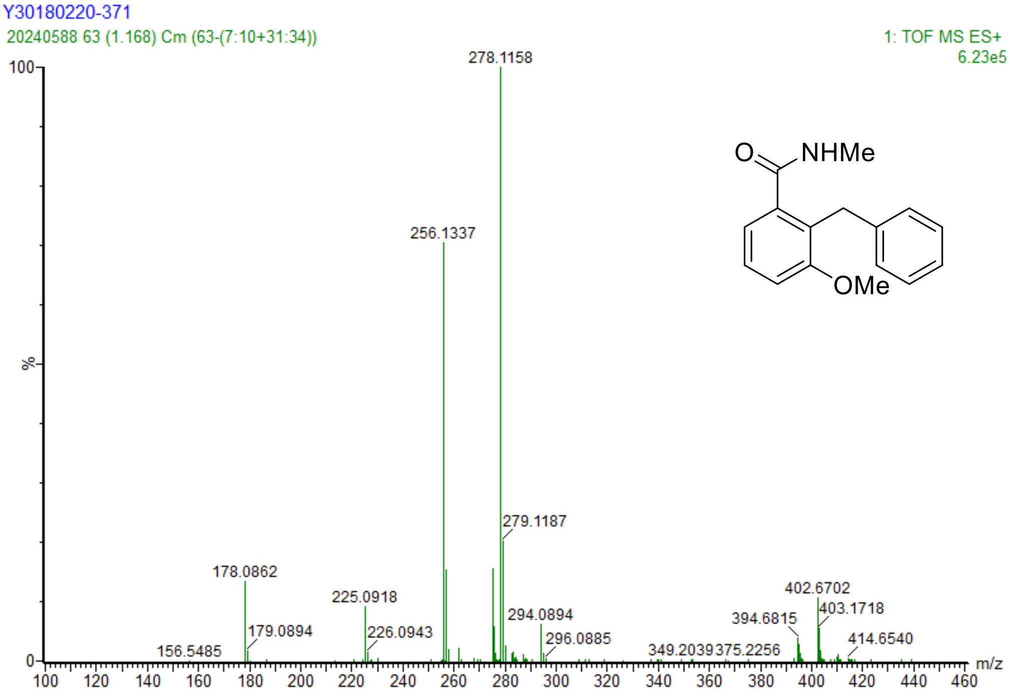

Elemental Composition Report

Multiple Mass Analysis: 2 mass(es) processed

Tolerance = 5.0 PPM / DBE: min = -1.5, max = 50.0

Element prediction: Off

Number of isotope peaks used for i-FIT = 3

Monoisotopic Mass, Even Electron Ions

7935 formula(e) evaluated with 10 results within limits (all results (up to 1000) for each mass)

Elements Used:

12C: 0-60 13C: 0-1 H: 0-60 N: 0-8 O: 0-8 Na: 0-1

Minimum:		15.00				-1.5			
Maximum:		100.00		20.0	5.0	50.0			
Mass	RA	Calc. Mass	mDa	PPM	DBE	i-FIT	Norm	Conf(%)	Formula
278.1158	100.00	278.1158	0.0	0.0	0.5	81.1	2.231	10.75	12C6 13C H18 N4 O6 Na

HRMS of 3oa

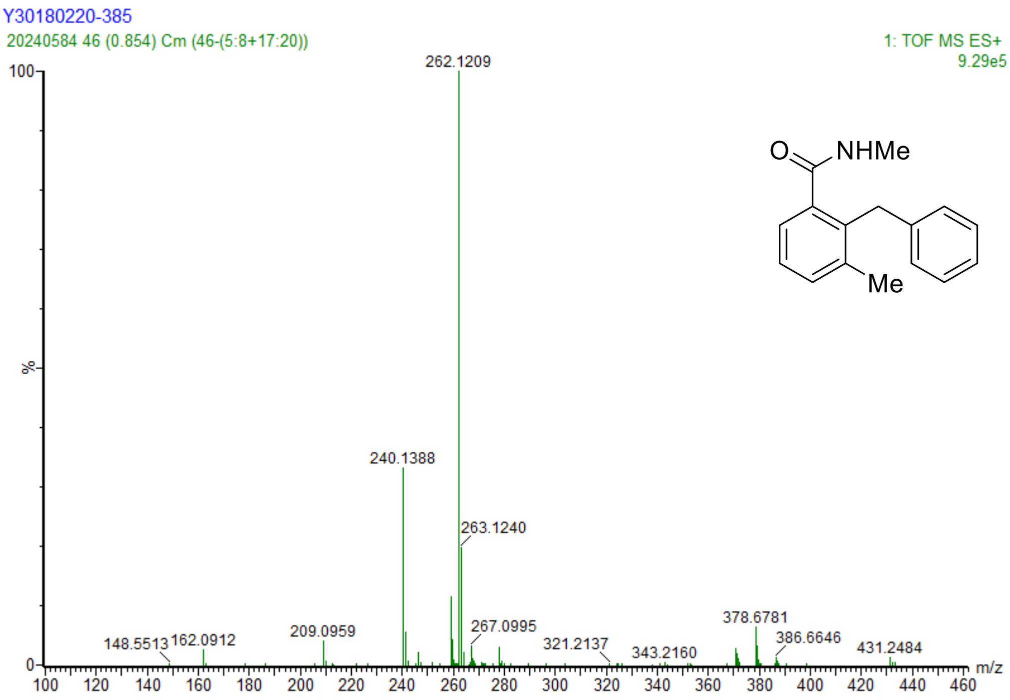

Elemental Composition Report

Multiple Mass Analysis: 2 mass(es) processed

Tolerance = 5.0 PPM / DBE: min = -1.5, max = 50.0

Element prediction: Off

Number of isotope peaks used for i-FIT = 3

Monoisotopic Mass, Even Electron Ions

7073 formula(e) evaluated with 6 results within limits (all results (up to 1000) for each mass)

Elements Used:

12C: 0-60 13C: 0-1 H: 0-60 N: 0-8 O: 0-8 Na: 0-1

Minimum:		15.00				-1.5		
Maximum:		100.00		20.0	5.0	50.0		
Mass	RA	Calc. Mass	mDa	PPM	DBE	i-FIT	Norm	Conf(%) Formula
262.1209	100.00	262.1208	0.1	0.4	0.5	132.7	1.797	16.58 12C6 13C H18 N4 O5 Na

HRMS of 3ab

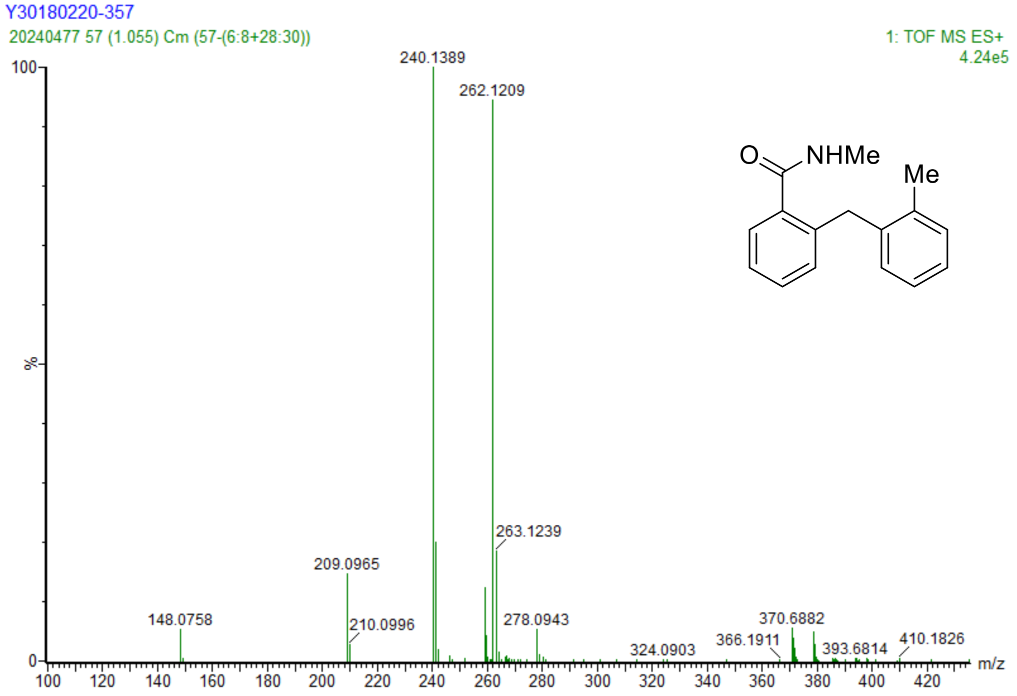

Elemental Composition Report

Multiple Mass Analysis: 2 mass(es) processed

Tolerance = 5.0 PPM / DBE: min = -1.5, max = 55.0

Element prediction: Off

Number of isotope peaks used for i-FIT = 3

Monoisotopic Mass, Even Electron Ions

5900 formula(e) evaluated with 7 results within limits (all results (up to 1000) for each mass)

Elements Used:

12C: 0-60 13C: 0-1 H: 0-60 N: 0-8 O: 0-8 Na: 0-1

Minimum:	10.00				-1.5		
Maximum:	100.00		20.0	5.0	55.0		
Mass	RA	Calc. Mass	mDa	PPM	DBE	i-FIT	Norm
Conf(%)	Formula						
240.1389	100.00	240.1389	0.0	0.0	0.5	48.2	2.025
13.20	12C6 13C H19 N4 O5						

HRMS of 3ac

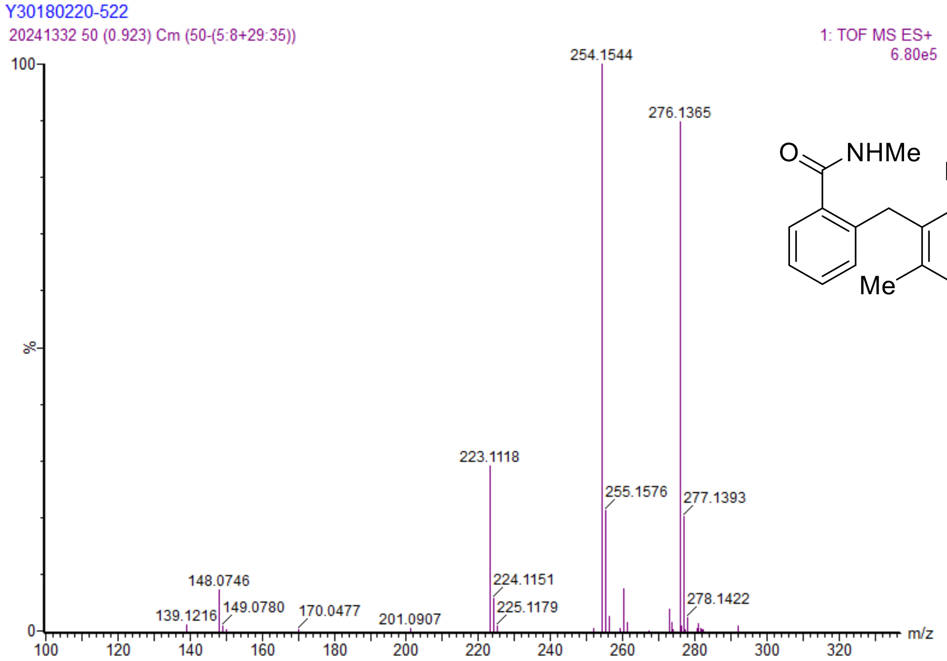

Elemental Composition Report

Multiple Mass Analysis: 2 mass(es) processed

Tolerance = 5.0 PPM / DBE: min = -1.5, max = 50.0

Element prediction: Off

Number of isotope peaks used for i-FIT = 3

Monoisotopic Mass, Even Electron Ions

6641 formula(e) evaluated with 7 results within limits (all results (up to 1000) for each mass)

Elements Used:

12C: 0-60 13C: 0-1 H: 0-60 N: 0-8 O: 0-8 Na: 0-1

Minimum:		15.00				-1.5		
Maximum:		100.00		20.0	5.0	50.0		
Mass	RA	Calc. Mass	mDa	PPM	DBE	i-FIT	Norm	Conf(%) Formula
254.1544	100.00	254.1545	-0.1	-0.4	8.5	127.6	0.169	84.43 12C17 H20 N O

HRMS of 3ad

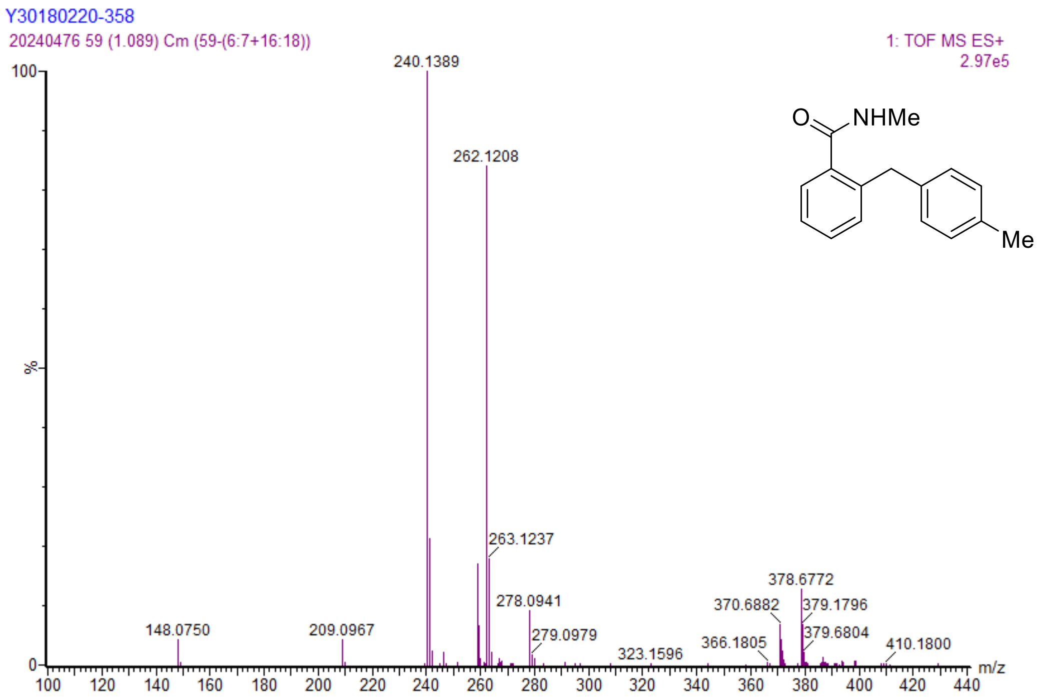

Elemental Composition Report

Multiple Mass Analysis: 2 mass(es) processed

Tolerance = 5.0 PPM / DBE: min = -1.5, max = 55.0

Element prediction: Off

Number of isotope peaks used for i-FIT = 3

Monoisotopic Mass, Even Electron Ions

5900 formula(e) evaluated with 7 results within limits (all results (up to 1000) for each mass)

Elements Used:

12C: 0-60 13C: 0-1 H: 0-60 N: 0-8 O: 0-8 Na: 0-1

Minimum:		10.00				-1.5	
Maximum:		100.00		20.0	5.0	55.0	
Mass	RA	Calc. Mass	mDa	PPM	DBE	i-FIT	Norm
Conf(%)	Formula						
240.1389	100.00	240.1389	0.0	0.0	0.5	47.5	1.998
13.56	12C6 13C H19 N4 O5						

HRMS of 3ae

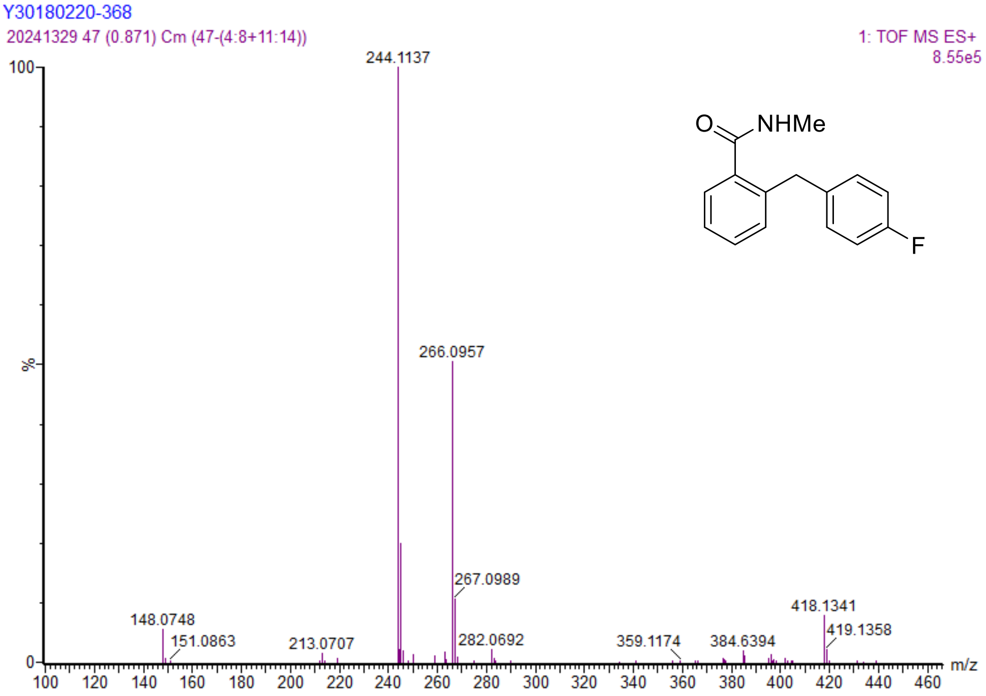

Elemental Composition Report

Multiple Mass Analysis: 2 mass(es) processed

Tolerance = 5.0 PPM / DBE: min = -1.5, max = 50.0

Element prediction: Off

Number of isotope peaks used for i-FIT = 3

Monoisotopic Mass, Even Electron Ions

5117 formula(e) evaluated with 7 results within limits (all results (up to 1000) for each mass)

Elements Used:

12C: 0-60 13C: 0-1 H: 0-60 N: 0-8 O: 0-8 F: 1-1 Na: 0-1

Minimum:		15.00			-1.5			
Maximum:		100.00		20.0	5.0	50.0		
Mass	RA	Calc. Mass	mDa	PPM	DBE	i-FIT	Norm	Conf(%) Formula
244.1137	100.00	244.1138	-0.1	-0.4	8.5	137.3	0.321	72.56 12C15 H15 N O F

HRMS of 3af

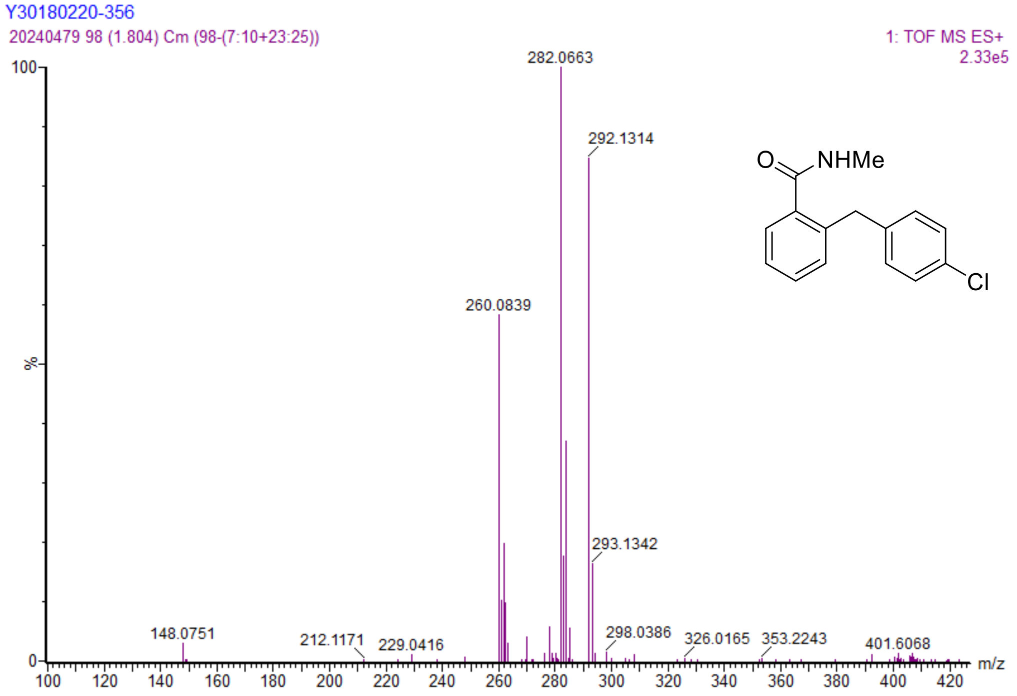

Elemental Composition Report

Multiple Mass Analysis: 4 mass(es) processed

Tolerance = 5.0 PPM / DBE: min = -1.5, max = 55.0

Element prediction: Off

Number of isotope peaks used for i-FIT = 3

Monoisotopic Mass, Even Electron Ions

9888 formula(e) evaluated with 12 results within limits (all results (up to 1000) for each mass)

Elements Used:

12C: 0-60 13C: 0-1 H: 0-60 N: 0-2 O: 0-2 Na: 0-1 35Cl: 0-1 37Cl: 0-1

Minimum:		5.00				-1.5			
Maximum:		100.00		20.0	5.0	55.0			
Mass	RA	Calc. Mass	mDa	PPM	DBE	i-FIT	Norm	Conf(%)	Formula
282.0663	100.00	282.0662	0.1	0.4	8.5	51.7	2.474	8.43	12C15 H14 N O Na 35Cl

HRMS of 3ag

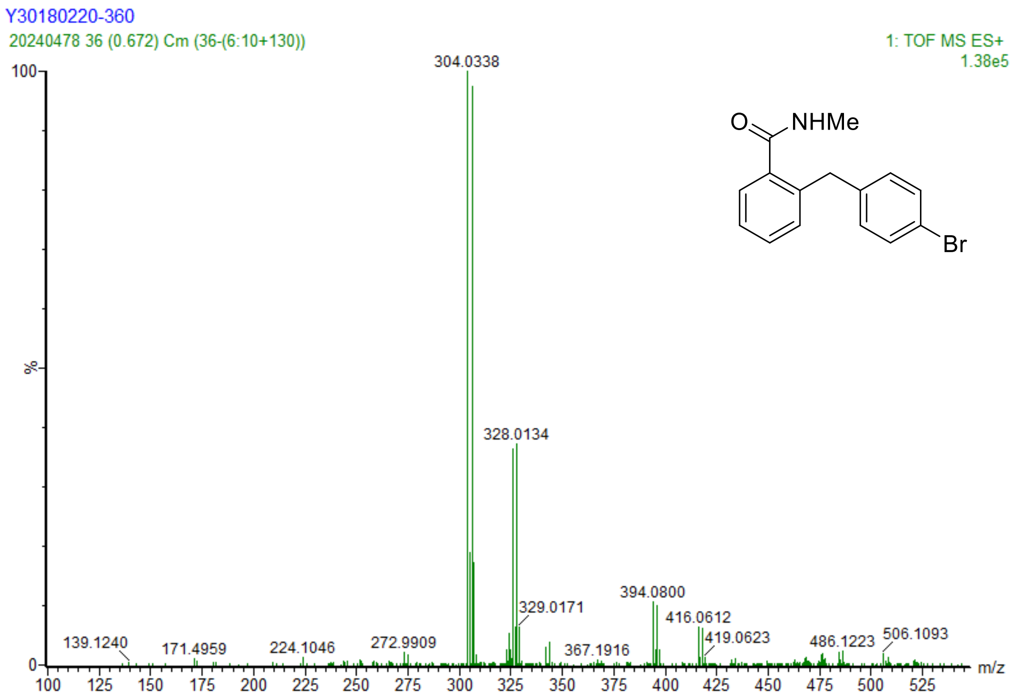

Elemental Composition Report

Multiple Mass Analysis: 4 mass(es) processed

Tolerance = 5.0 PPM / DBE: min = -1.5, max = 55.0

Element prediction: Off

Number of isotope peaks used for i-FIT = 3

Monoisotopic Mass, Even Electron Ions

8832 formula(e) evaluated with 11 results within limits (all results (up to 1000) for each mass)

Elements Used:

12C: 0-60 13C: 0-1 H: 0-60 N: 0-2 O: 0-2 Na: 0-1 79Br: 0-1 81Br: 0-1

Minimum: 10.00 -1.5

Maximum: 100.00 20.0 5.0 55.0

Mass RA Calc. Mass mDa PPM DBE i-FIT Norm Conf(%) Formula

304.0338 100.00 304.0337 0.1 0.3 8.5 63.4 2.144 11.72 12C15 H15 N O 79Br

HRMS of 3ah

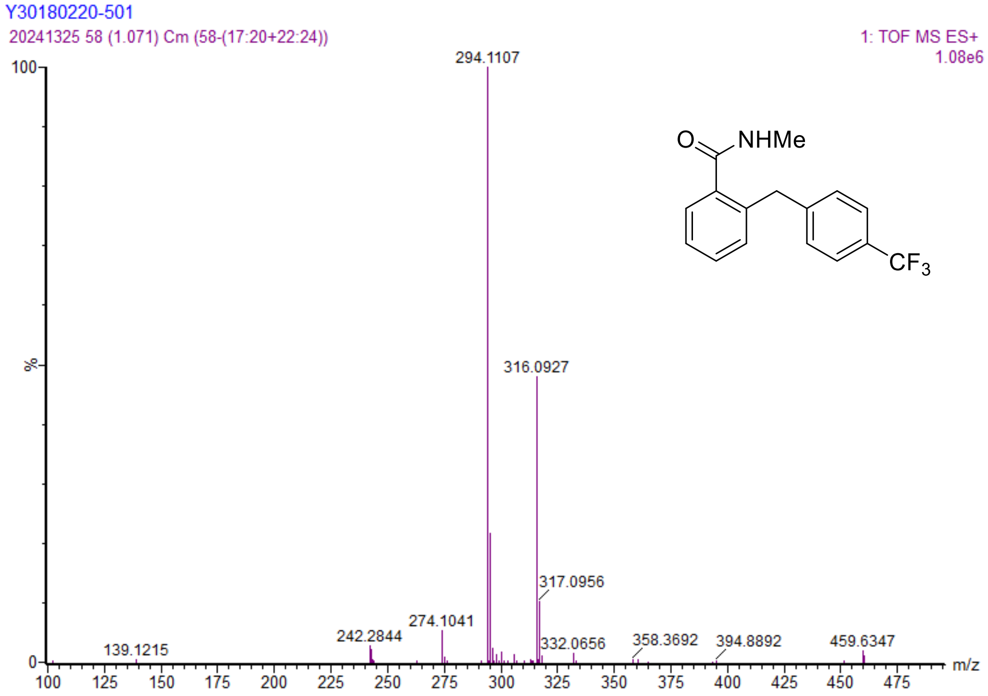

Elemental Composition Report

Multiple Mass Analysis: 2 mass(es) processed

Tolerance = 5.0 PPM / DBE: min = -1.5, max = 50.0

Element prediction: Off

Number of isotope peaks used for i-FIT = 3

Monoisotopic Mass, Even Electron Ions

5741 formula(e) evaluated with 8 results within limits (all results (up to 1000) for each mass)

Elements Used:

12C: 0-60 13C: 0-1 H: 0-60 N: 0-8 O: 0-8 F: 3-3 Na: 0-1

Minimum: 15.00 -1.5

Maximum: 100.00 20.0 5.0 50.0

Mass RA Calc. Mass mDa PPM DBE i-FIT Norm Conf(%) Formula

294.1107 100.00 294.1106 0.1 0.3 8.5 189.2 0.437 64.57 12C16 H15 N O F3

HRMS of 3ai

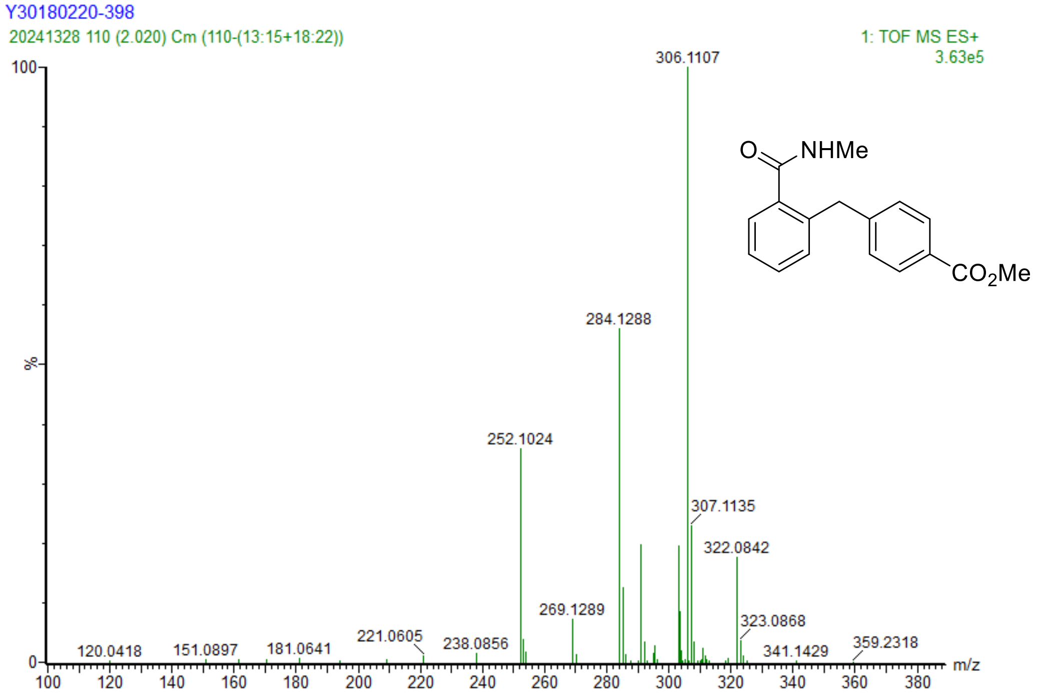

Elemental Composition Report

Multiple Mass Analysis: 2 mass(es) processed

Tolerance = 5.0 PPM / DBE: min = -1.5, max = 50.0

Element prediction: Off

Number of isotope peaks used for i-FIT = 3

Monoisotopic Mass, Even Electron Ions

9447 formula(e) evaluated with 11 results within limits (all results (up to 1000) for each mass)

Elements Used:

12C: 0-60 13C: 0-1 H: 0-60 N: 0-8 O: 0-8 Na: 0-1

Minimum: 15.00 -1.5

Maximum: 100.00 20.0 5.0 50.0

Mass RA Calc. Mass mDa PPM DBE i-FIT Norm Conf(%) Formula

306.1107 100.00 306.1107 0.0 0.0 1.5 76.0 2.683 6.84 12C7 13C H18 N4 O7 Na

HRMS of 3aj

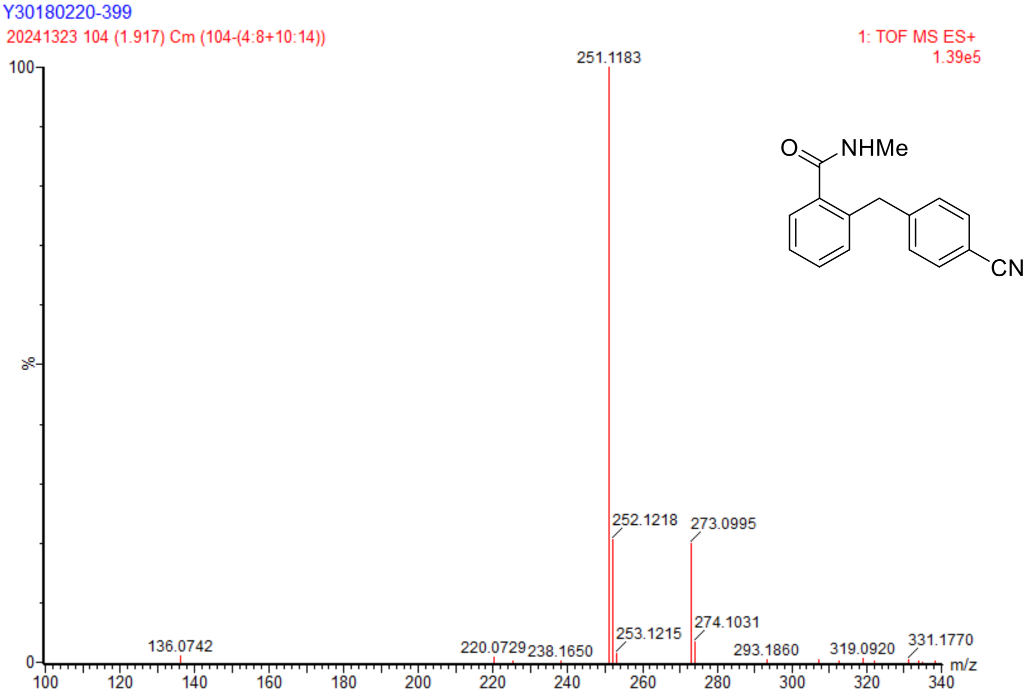

Elemental Composition Report

Multiple Mass Analysis: 2 mass(es) processed

Tolerance = 5.0 PPM / DBE: min = -1.5, max = 50.0

Element prediction: Off

Number of isotope peaks used for i-FIT = 3

Monoisotopic Mass, Even Electron Ions

6483 formula(e) evaluated with 8 results within limits (all results (up to 1000) for each mass)

Elements Used:

12C: 0-60 13C: 0-1 H: 0-60 N: 0-8 O: 0-8 Na: 0-1

Minimum:		15.00				-1.5			
Maximum:		100.00		20.0	5.0	50.0			
Mass	RA	Calc. Mass	mDa	PPM	DBE	i-FIT	Norm	Conf(%)	Formula
251.1183	100.00	251.1184	-0.1	-0.4	10.5	33.6	1.069	34.33	12C16 H15 N2 O
